# Population genomics shows no distinction between pathogenic *Candida krusei* and environmental *Pichia kudriavzevii*: One species, four names

**DOI:** 10.1371/journal.ppat.1007138

**Published:** 2018-07-19

**Authors:** Alexander P. Douglass, Benjamin Offei, Stephanie Braun-Galleani, Aisling Y. Coughlan, Alexandre A. R. Martos, Raúl A. Ortiz-Merino, Kevin P. Byrne, Kenneth H. Wolfe

**Affiliations:** UCD Conway Institute, School of Medicine, University College Dublin, Dublin, Ireland; Carnegie Mellon University, UNITED STATES

## Abstract

We investigated genomic diversity of a yeast species that is both an opportunistic pathogen and an important industrial yeast. Under the name *Candida krusei*, it is responsible for about 2% of yeast infections caused by *Candida* species in humans. Bloodstream infections with *C*. *krusei* are problematic because most isolates are fluconazole-resistant. Under the names *Pichia kudriavzevii*, *Issatchenkia orientalis* and *Candida glycerinogenes*, the same yeast, including genetically modified strains, is used for industrial-scale production of glycerol and succinate. It is also used to make some fermented foods. Here, we sequenced the type strains of *C*. *krusei* (CBS573^T^) and *P*. *kudriavzevii* (CBS5147^T^), as well as 30 other clinical and environmental isolates. Our results show conclusively that they are the same species, with collinear genomes 99.6% identical in DNA sequence. Phylogenetic analysis of SNPs does not segregate clinical and environmental isolates into separate clades, suggesting that *C*. *krusei* infections are frequently acquired from the environment. Reduced resistance of strains to fluconazole correlates with the presence of one gene instead of two at the *ABC11-ABC1* tandem locus. Most isolates are diploid, but one-quarter are triploid. Loss of heterozygosity is common, including at the mating-type locus. Our PacBio/Illumina assembly of the 10.8 Mb CBS573^T^ genome is resolved into 5 complete chromosomes, and was annotated using RNAseq support. Each of the 5 centromeres is a 35 kb gene desert containing a large inverted repeat. This species is a member of the genus *Pichia* and family Pichiaceae (the methylotrophic yeasts clade), and so is only distantly related to other pathogenic *Candida* species.

## Author summary

Infections with yeasts resistant to antifungal drugs are an increasing cause of concern. One species, *Candida krusei*, has innate resistance to the widely-used drug fluconazole. It is one of the five most prevalent causes of clinical yeast infections, and is responsible for significant levels of morbidity and mortality in immunocompromised patients. In this study, we show that *C*. *krusei* is the same species as *Pichia kudriavzevii*, a yeast that is regarded as non-pathogenic and has important applications in biotechnology and food industries. We examined the genomes of 20 clinical isolates (‘*C*. *krusei*’) and 12 environmental isolates (‘*P*. *kudriavzevii*’) and find that there is no genetic distinction between them. The environmental isolates have similar levels of drug resistance to the clinical isolates. As well as providing a resource for future studies of this yeast, our results indicate that caution may be needed in the use of drug-resistant *P*. *kudriavzevii* strains for biotechnology and food applications.

## Introduction

Pathogenic *Candida* species are ascomycete yeasts that cause over 46,000 invasive infections annually in the US alone, with a 30% mortality rate [1]. *C*. *albicans* is the most common and the most extensively studied, but non-*albicans* candidiasis infections are becoming increasingly common. The top five pathogenic *Candida* species in order of prevalence in invasive candidiasis worldwide are *C*. *albicans* (52% of infections), *C*. *glabrata* (21%), *C*. *tropicalis* (14%), *C*. *parapsilosis* (9%) and *C*. *krusei* (2%) (calculated from data in [2]). Among these, *C*. *krusei* is the least-well studied. Although uncommon in the normal human flora, *C*. *krusei* is sometimes carried intestinally by healthy individuals and in one remote Amerindian community it was found to be present in over 30% of the population, much higher than *C*. *albicans*, and was probably acquired from food or the environment [3]. As well as being associated with humans, *C*. *krusei* has been detected in feral pigeons and other wild animals [3, 4].

In 1980, Kurtzman and colleagues proposed that *C*. *krusei* is the asexual form (anamorph) of a species whose sexual form (teleomorph) is *Pichia kudriavzevii*, which would make the two names synonymous. This proposal was initially made on the basis of DNA reassociation and mating tests [5], and later confirmed when the sequences of the D1/D2 regions of the 26S ribosomal DNA of the type strains of *C*. *krusei* and *P*. *kudriavzevii* were discovered to be identical [6]. When *P*. *kudriavzevii* was first formally described in 1960 [7], it was reported to be able to sporulate, but cultures of the type strain were later described as being unable to conjugate or sporulate [8, 9]. A third name, *Issatchenkia orientalis*, is obsolete [6] but continues to be used in the literature by some laboratories [10, 11]. Strain CBS5147 was deposited as the type strain of *I*. *orientalis* [7], but this species was later renamed *P*. *kudriavzevii* [6]; *C*. *krusei* has a different type strain, CBS573.

*P*. *kudriavzevii* isolates are widely distributed in nature. They are often encountered in spontaneous fermentations and the species is used to produce several traditional fermented foods [9, 12]. Crucially, this yeast is not regarded as a pathogen. It has been given ‘generally recognized as safe’ status by the US Food and Drug Administration [13] because it has been used for centuries to make food products such as fermented cassava and cacao in Africa, fermented milk in Tibet and Sudan, and maize beverages in Colombia [13]. It is used in starter cultures for sourdough breads [14], and in starters (daqu) for Chinese vinegar production from wheat [15]. It also has potential as a probiotic [16]. *P*. *kudriavzevii* is exceptionally stress-tolerant and has a growing role in biotechnology, for production of bioethanol [17, 18] and succinic acid (a high-value platform chemical) [10]. It is also used for the industrial production of glycerol, under the name *Candida glycerinogenes* ([19]; see Discussion). Publications related to industrial applications generally use the species names *P*. *kudriavzevii*, *I*. *orientalis* or *C*. *glycerinogenes* in preference to *C*. *krusei*, possibly because of the negative safety connotations of using a pathogen in a biotechnological or food context.

To date, relatively little genetic or genomic investigation has been carried out on isolates of *C*. *krusei* and *P*. *kudriavzevii*. Genome sequences have been published for four *P*. *kudriavzevii* strains ([10, 20–22]) and one *C*. *krusei* clinical isolate [23], but none of these provides a chromosome-level assembly or transcriptome-based annotation. Estimates of the number of genes range from 4949 to 7107 [10, 23], and only Cuomo *et al*. [23] discussed the genome organization and content. Moreover, the type strain of *C*. *krusei* has not been sequenced, and the only available sequence for the type strain of *P*. *kudriavzevii* is highly fragmented (Y. Takada *et al*., NCBI accession number BBOI01000000). Although an extensive study of genetic diversity in clinical isolates was conducted using multilocus sequence typing (MLST) [24], there has never been an analysis that compares both clinical (‘*C*. *krusei*’) and environmental (‘*P*. *kudriavzevii*’) isolates. As a result we do not know whether the clinical and environmental isolates are genetically distinct. It is important to understand the relatedness of these two types of isolate, because if there is no difference between them it could mean that environmental and industrial strains are capable of causing disease. For instance, *Saccharomyces cerevisiae* used in food products is capable of causing opportunistic infections [25], so the same could be true of *P*. *kudriavzevii*. There is also uncertainty about the ploidy of the species. The MLST study indicated that isolates are diploid [24], as did two genome analyses [10, 23], but evidence of triploid and aneuploid strains has also been reported [26].

*C*. *krusei* is of particular concern as a pathogen because of its intrinsic resistance to fluconazole, a drug commonly used for long-term antifungal prophylactic treatment of immunocompromised individuals [27, 28]. Fluconazole resistance in *C*. *krusei* is not fully understood but appears to have two causes: its ergosterol synthesis enzyme Erg11 has unusually low affinity for fluconazole, and the drug efflux pumps Abc1 and Abc11 are constitutively expressed [26, 27]. Echinocandins such as micafungin are the current drugs of choice for treatment of *C*. *krusei* infections, but echinocandin-resistant strains with point mutations in the *FKS1* gene have been reported [29, 30]. Additionally, it is possible that drug resistance may differ between clinical and environmental strains, but this has not been investigated.

The very large evolutionary distance between *C*. *krusei* and other pathogenic *Candida* species is often not appreciated in clinical settings. By phylogenetic analysis of rDNA and other genes, systematists have determined that *P*. *kudriavzevii/C*. *krusei* is a species in the genus *Pichia* which is in the family Pichiaceae, often called the methylotrophic yeasts [9, 31]. It uses the universal genetic code (CUG = Leu) [32]. *C*. *krusei* (family Pichiaceae), *C*. *albicans* (family Debaryomycetaceae) and *C*. *glabrata* (family Saccharomycetaceae) are as distantly related to each other as humans are to sea-squirts [33] and it is rather misleading that they are all named *Candida* (which simply means that a sexual cycle has not been observed in any of them). Apart from *C*. *krusei*, the only other known pathogens in family Pichiaceae are the rare species *Pichia norvegensis* (also called *Candida norvegensis*) and *Pichia cactophila* (also called *Candida inconspicua*) [34].

To better understand their genetics, phylogeny, and drug resistance, we sequenced the type strains of both *C*. *krusei* and *P*. *kudriavzevii*, as well as 30 other clinical and environmental isolates. We generated a high-quality reference genome for *C*. *krusei* CBS573^T^, using a combined PacBio/Illumina strategy to assemble complete sequences of its 5 nuclear chromosomes and its mitochondrial genome, and annotated it using RNAseq data to detect introns. We investigated genetic diversity, ploidy, and loss of heterozygosity, as well as centromere and mating-type locus structure. Our results show unequivocally that *C*. *krusei* and *P*. *kudriavzevii* are the same species, that clinical and environmental strains are not distinct, and that high levels of drug resistance are common in environmental isolates. Our work provides a resource for future molecular biology research on this yeast species that has four names and is both an emerging pathogen and an emerging workhorse for biotechnology.

## Results

### Genome organization and content

In this section, we describe the construction of a PacBio/Illumina reference genome sequence for the type strain of *C*. *krusei* CBS573, and PacBio sequencing of the *P*. *kudriavzevii* type strain CBS5147. We then comment on the content of genes and mobile genetic elements, and on several other features: the centromeres, ribosomal DNA, telomeres, mitochondrial genome, introns, ribosomal protein genes, *MAT* locus and pheromone genes.

#### *C*. *krusei* CBS573^T^ reference genome sequencing and annotation

We sequenced the genome of the type strain of *C*. *krusei* (CBS573) using Pacific Biosciences (PacBio) technology, in combination with Illumina data for correction of insertion/deletion errors. We obtained five near-complete chromosome sequences (Table 1). The number of chromosomes and their sizes agree with a previous estimate for a clinical isolate studied by pulsed-field gel electrophoresis [23]. Genes were annotated using YGAP [35], which annotates protein-coding genes by virtue of their sequence similarity and synteny to genes in *S*. *cerevisiae* and other yeasts in family Saccharomycetaceae. We used RNA-seq transcriptome data from CBS573 cultures grown in YPD media to help annotate intron/exon structures manually. Objective evaluation of the quality of annotation using BUSCO [36] shows that our annotation of the CBS573 genome has more complete genes, and fewer missing or fragmented genes, than all previous annotations of *C*. *krusei* or *P*. *kudriavzevii* (S1 Table).

**Table 1:** Statistics for the *P*. *kudriavzevii* CBS573 genome.

Chromosome	Length	Protein-coding genes	Introns*	tRNA genes
1	2.852 Mb	1346	38	54
2	2.746 Mb	1322	46	31
3	2.542 Mb	1204	59	34
4	1.384 Mb	670	30	31
5	1.289 Mb	598	32	24
Nuclear genome	10.813 Mb	5140	205	174
mtDNA	51340 bp	15	0	25

*Excluding tRNA introns.

#### *P*. *kudriavzevii* CBS5147^T^ genome sequencing

We also sequenced the genome of the type strain of *P*. *kudriavzevii* (CBS5147) by PacBio. It assembled into 13 contigs but contained a higher level of indel errors than CBS573, so we used CBS573 as the reference genome sequence for annotation and downstream analyses. We merged overlaps between the CBS5147 PacBio contigs manually to make 5 chromosome sequences. Alignment to the CBS573 chromosomes using MUMmer [37] showed that the two type strains have 99.6% nucleotide sequence identity, and dot-matrix plots showed that the two genomes are completely collinear (S1 Fig). The only exception to collinearity is some retrotransposons (PkudTy3A elements, described below) that are in different locations in the two strains. Because the genomes of the type strains are so similar, in the remainder of this manuscript we use a single name, *P*. *kudriavzevii*, for the species represented by CBS573 and CBS5147, except in the particular context of clinical isolates.

#### Gene content

The CBS573 nuclear genome is 10.8 Mb in size, similar to other species in the family Pichiaceae. The annotated nuclear genome contains 5140 protein-coding genes (Table 1). 174 tRNA genes were detected using tRNAscan-SE [38]. Of the protein-coding genes discovered, 3847 have homologs in *S*. *cerevisiae* and a further 607 genes were annotated at the protein domain (Pfam) level. The protein-coding genes are densely packed and there is little repetitive DNA. We identified two families of Ty3-like (*gypsy*) elements, termed PkudTy3A and PkudTy3B. There are multiple pseudogenes of both families in the CBS573 genome, but there are only 6 intact PkudTy3A elements (*PKUD0B03720*, *PKUD0C00880*, *PKUD0C11510*, *PKUD0D01600*, *PKUD0D01620*, *PKUD0D02390*) and no intact copies of PkudTy3B. Translation of the PkudTy3A elements appears to require an unusual -1 ribosomal frameshifting event, in contrast to the +1 frameshifting that occurs in most other characterized Ty elements [39]. Although it is a member of the ‘methylotrophic yeasts’ clade (defined as family Pichiaceae and the genus *Komagataella*; [31]), neither the CBS573 nor CBS5147 genomes contains a methanol oxidase gene (*MOX1/AOX1*) and neither of them can grow on methanol as a sole carbon source [9].

#### Centromeres

**Fig 1:**
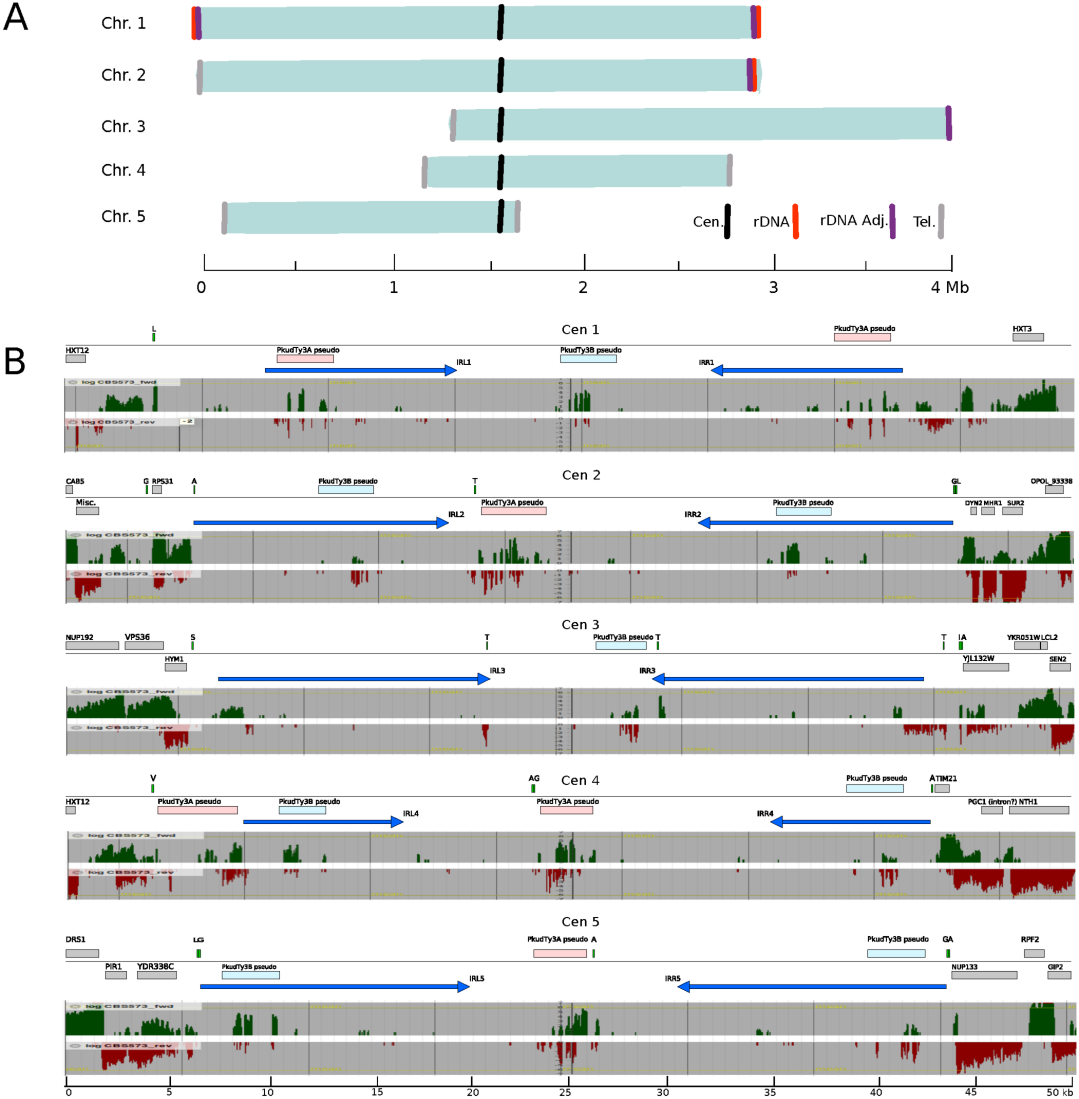
Chromosome and centromere structure in *P*. *kudriavzevii*. (A) Organization of the five chromosomes, aligned at their centromeres. Locations of centromeres (black), telomeres (gray), rDNA (red) and repeated sequences adjacent to rDNA (purple) are shown. (B) Centromere organization. For each chromosome, the upper panel shows a map of features in a 50 kb window spanning the centromere, and the lower panel shows RNAseq transcription data (green, forward strand; red, reverse strand; yellow lines show the mean level of transcription on the chromosome). Large blue arrows show the locations of the pairs of sequences that form an inverted repeat (IR) on each chromosome. Rectangles show protein-coding genes (gray), tRNA genes (green, with one-letter amino acid code), and pseudogenes of PkudTy3A (pink) and PkudTy3B (cyan) retroelements.

The CBS573 genome’s centromeres were immediately apparent because each of them contains a single large inverted repeat (IR), as well as being devoid of genes (Fig 1A and 1B; S1 Fig). These structures resemble the centromeres of *Komagataella phaffii* [40] and *Candida tropicalis* [41]. The IRs consist of pairs of sequences in opposite orientations, that range from 7.9 to 14.5 kb long, and have 99% DNA sequence identity in each case. The two parts of the IR are separated by a central region of 8.0–18.3 kb, making the total length of the centromeres 31.7–37.8 kb. There is a complex relationship of sequence similarity between the IRs of some centromeres and the central regions of other centromeres. For example, the IRs of chromosome 1 are similar to the central region of chromosome 2, and *vice versa* (S2 Fig). Centromeres 1 and 2 appear to form a similar pair, as do centromeres 4 and 5. The centromere regions contain no protein-coding genes, though 21 tRNA genes are present, as are some pseudogenes of PkudTy3 elements. tRNA genes are present about 5 times more frequently at centromeres than would be expected if they were randomly distributed in the genome (*P* = 0.0006, two-tailed Fisher’s exact test). The only evidence of transcription in centromeric regions was at PkudTy3 elements, which may be mismapped RNAseq reads derived from the intact PkudTy3 loci in the genome.

#### rDNA and telomeres

Each of the five chromosome sequences has either a telomere or a ribosomal DNA (rDNA) repeat unit at its ends (Fig 1A). rDNA is present at both ends of chromosome 1 (1L and 1R), and at the right end of chromosome 2 (2R). Each rDNA unit contains 18S, 5.8S, and 26S rRNA genes transcribed towards the end of the chromosome, and a 5S gene in the opposite orientation. At chromosome 3R, our sequence ends in a region that is highly similar to sequences upstream of the rDNA regions on chromosomes 1 and 2, so we infer that there is probably a fourth rDNA unit at chromosome 3R. There are no other rDNA loci in the genome. Telomere repeats with a 28 bp consensus sequence (TTACAATATGAACTAGGAGCGAGGTGTG), which is long relative to other yeasts [42], are found at the other six chromosome ends (2L, 3L, 4L, 4R, 5L, 5R). On chromosome 2R, a single protein-coding gene is located beyond the rDNA at the right end and we do not know if there is also a telomere at this end.

#### Mitochondrial DNA

The mitochondrial genome is a 51 kb circle with very high A+T content (84%). It contains orthologs of all the *S*. *cerevisiae* mitochondrial genes including the ribosomal protein gene *RPS3* (*VAR1*). It includes genes for 7 NADH dehydrogenase subunits, which are also present in *C*. *albicans* but have been lost in *S*. *cerevisiae* and other Saccharomycetaceae species. All the mitochondrial genes (15 proteins, 25 tRNAs and 2 rRNAs) are on the same DNA strand, and there are no introns in the mitochondrial genome.

#### Introns and ribosomal protein genes

**Fig 2:**
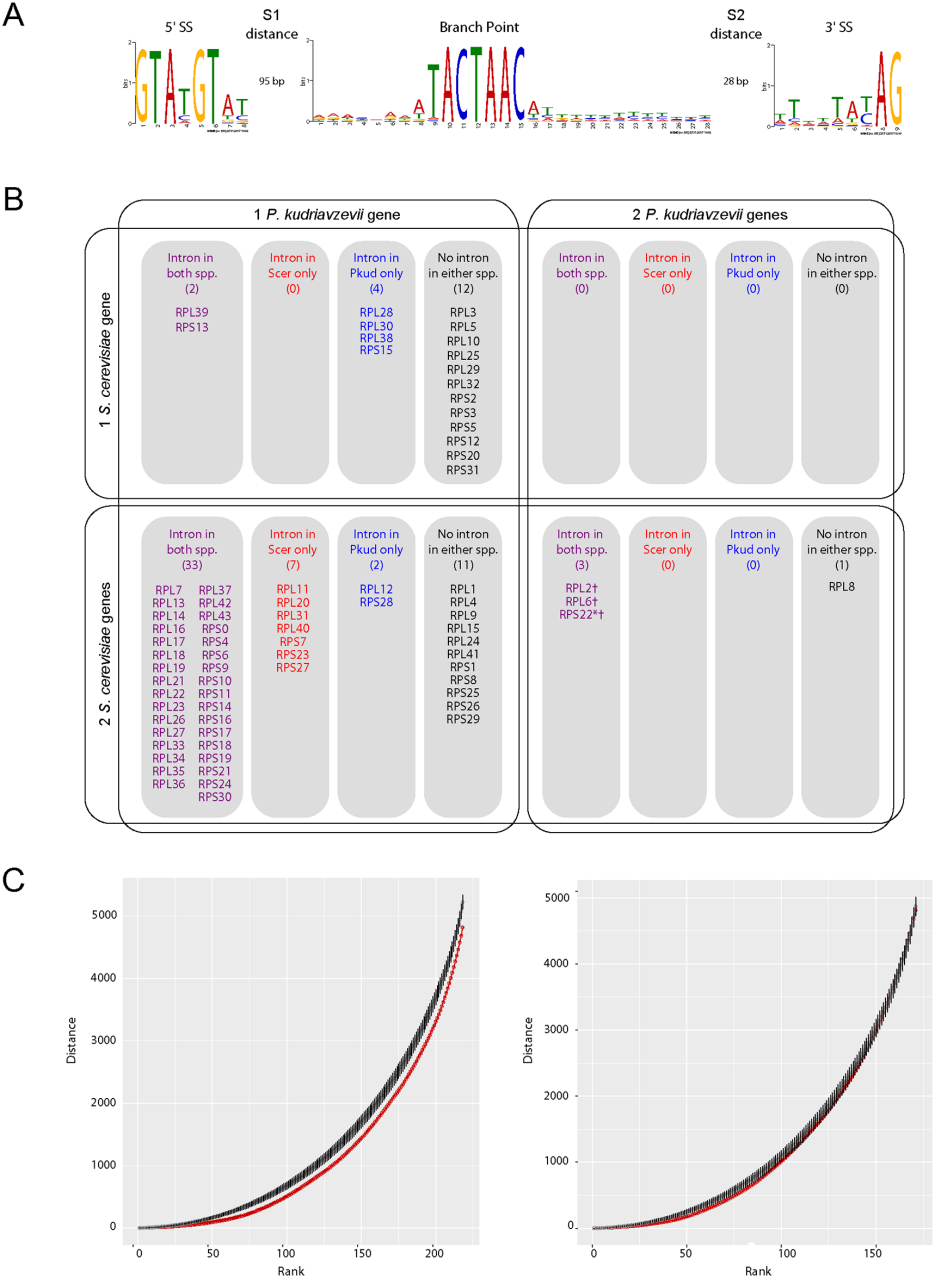
Intron and ribosomal protein gene content. (A) Intron consensus sequence for *P*. *kudriavzevii* CBS573 genes, generated using MEME [75]. Consensus sequences for the 5’ and 3’ splice sites (SS), and the branch point are shown, with the median distances (S1 and S2) between them, for all 205 introns in the nuclear genome. (B) Duplication and intron status of cytosolic ribosomal protein genes in *P*. *kudriavzevii* (Pkud) and *S*. *cerevisiae* (Scer). The four quadrants show the number of copies of each RP gene in the two species. Within each quadrant, the four columns show whether the gene contains an intron in both species (purple), in *S*. *cerevisiae* only (red), in *P*. *kudriavzevii* only (blue), or in neither species (black). Daggers (†) show cases where only 1 of the 2 copies of a gene in *P*. *kudriavzevii* contains an intron, and the asterisk (*) shows a case where only 1 of the 2 *S*. *cerevisiae* gene contains an intron. (C) Analysis of intron clustering. The distance, measured in genes, between each intron-containing gene and the next one (to its right in the genome) was calculated. The set of distances was then sorted so that introns close to other introns have low ranks. The plots show the running total of all distances up to a particular rank, for real introns (red points), and for 1000 simulated datasets in which introns were randomly assigned to genes (black points, with error bars ±1 s.d.). The plot on the left shows the result for all introns in the genome, and the plot on the right shows the result with ribosomal protein genes omitted.

We annotated 205 spliceosomal introns in nuclear protein-coding genes. Introns are present in 4% of genes, a similar level to *S*. *cerevisiae* [43]. The consensus splice site and branch site sequences are highly similar to those in *S*. *cerevisiae* (Fig 2A), though the distance (S2) from the branch site to the 3’ splice site is longer than in *S*. *cerevisiae*. Of the 205 spliceosomal introns, a quarter (43) are found in ribosomal protein (RP) genes. In *S*. *cerevisiae*, most RP genes are duplicated as a result of the whole-genome duplication, whereas their *P*. *kudriavzevii* orthologs are single-copy genes (only 4 of 74 *P*. *kudriavzevii* RP genes are duplicated). Intron content is generally conserved in RP genes between the two species, so that the genes either have an intron in both species, or there is no intron in either species (Fig 2B). Only 13 genes are discordant between the species. As previously noted [44], introns are rare in single-copy RP genes in *S*. *cerevisiae*.

When annotating introns, we noticed that intron-containing genes tended to occur in clusters in the *P*. *kudriavzevii* genome. To test whether this apparent clustering is statistically significant, we calculated the distance from each intron-containing gene to the next one in the genome, and compared this set of distances to sets obtained by simulation. The results show that greater clustering of introns occurs in the real genome than in 1000 simulations (Fig 2C, left panel). The two distributions are significantly different (*P* = 0.028 by Kolmogorov-Smirnov test). Most of this effect is due to clustering of RP genes in the genome. If the RP genes and their introns are ignored in the analysis, the introns in non-RP genes show no significant clustering (Fig 2C, right panel; *P* = 0.50).

#### *MAT* locus and pheromone genes

**Fig 3:**
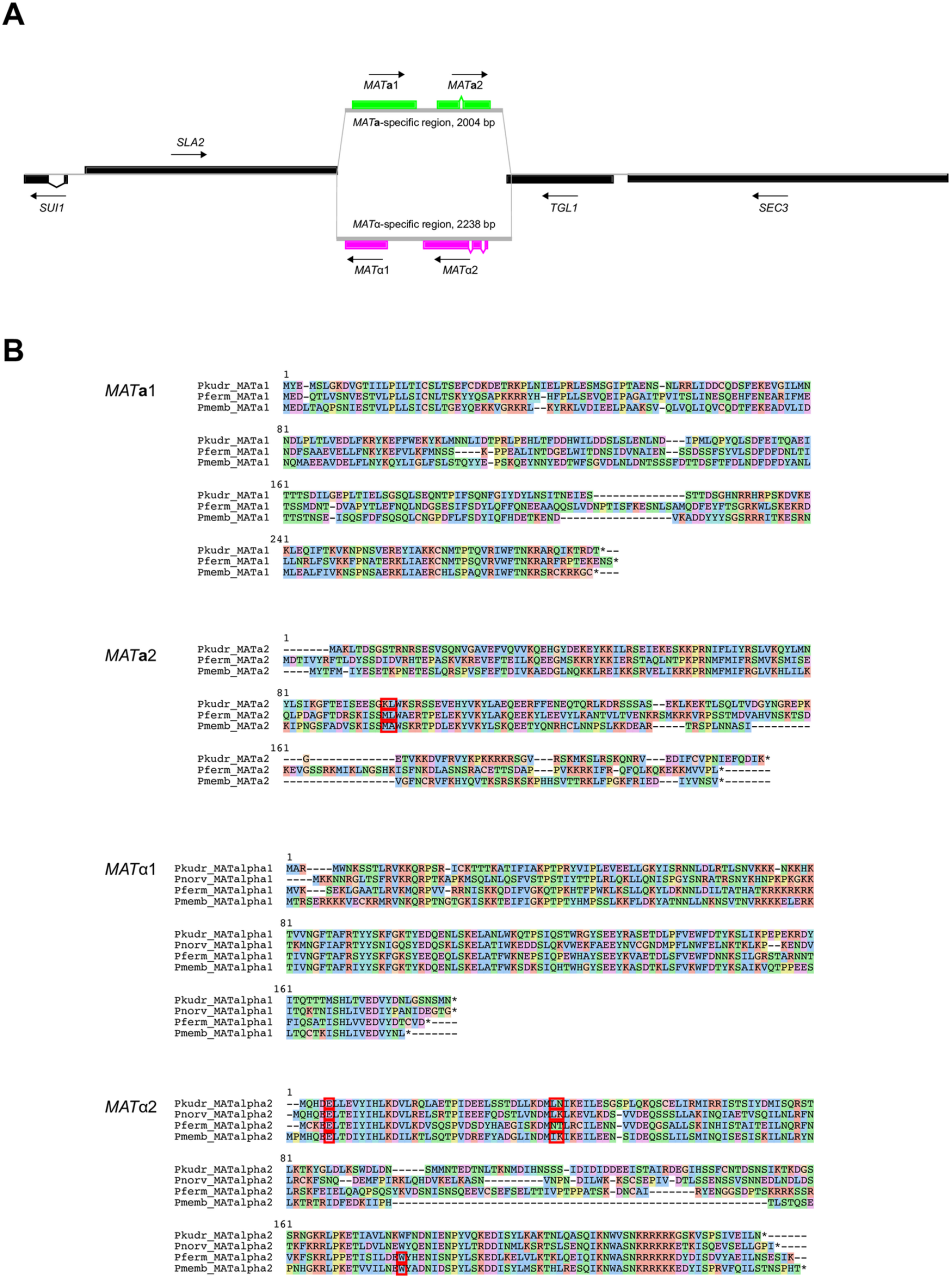
*P*. *kudriavzevii MAT* locus. (A) Organization of the *MAT* genes on CBS573 chromosome 2. (B) Sequence alignments of *MAT* proteins from *Pichia* species. Red boxes indicate intron locations. Colored backgrounds indicate conservative amino acid groups. Data for *P*. *kudriavzevii* (Pkudr), *P*. *fermentans* (Pferm), and *P*. *norvegensis* (Pnorv, *MAT*α genes only) is from this study, and *P*. *membranifaciens* data (Pmemb) is from Riley *et al*. [76].

Strain CBS573 is heterozygous at the mating-type (*MAT*) locus, which is located ~310 kb from the left end of chromosome 2 (Fig 3A). The assembled sequence of this chromosome contains *MAT***a**1 and *MAT***a**2 genes, and we identified a short PacBio contig containing the alternative allele with *MAT*α1 and *MAT*α2. The *MAT* genes neighbor *SUI1-SLA2* on one side and *TGL1-SEC3* on the other. *SUI1* and *SLA2* are commonly found beside *MAT* loci in other species, whereas *TGL1* and *SEC3* are not. The genome contains no silent loci, indicating this species is incapable of mating type switching and therefore heterothallic. Strain CBS5147 is also heterozygous at the *MAT* locus. The *MAT* proteins are quite poorly conserved among species in the genus *Pichia* (Fig 3B). In *MAT*α2, *P*. *kudriavzevii* and *P*. *norvegensis* have two introns whereas *P*. *fermentans* and *P*. *membranifaciens* have three.

We identified genes for both the α-factor and **a**-factor mating pheromones. The α-factor gene (*MF*α, *PKUD0A00810*) contains five repeats of a pheromone sequence WRWHKWFRNQAIY. The **a**-factor gene (*MF***a**, *PKUD0C04775*) codes for a 41-residue precursor ending in a putative farnesylation sequence CTIA. There is a pseudogene of *MF***a** upstream of *MF***a** itself. There is only one copy of the *MF*α gene.

### Phylogenetic position within the genus *Pichia*

Previous phylogenetic and phylogenomic analyses have established that *P*. *kudriavzevii* is a member of the genus *Pichia* and family Pichiaceae, often called the methylotrophic yeasts clade [6, 31]. Within the genus *Pichia*, one of the closest known relatives of *P*. *kudriavzevii* is *P*. *norvegensis*, also called *Candida norvegensis* [6]. Clinical infections with *P*. *norvegensis* have been reported [34]. We used Illumina sequencing to sequence the genomes of the type strain of *P*. *norvegensis* (originally isolated from sputum), and a strain of *P*. *fermentans* (from pickled cucumber [45]) that had been misidentified as *P*. *kudriavzevii* (Table 2).

**Table 2:** Summary of strains analyzed. Magenta and green highlighting indicates strains designated relatively resistant (RR) or relatively sensitive (RS) to each drug, respectively.

Species	Code	Original Designation	Source	Country	Ploidy	*MAT* genotype	Illumina Coverage	Fluconazole MIC (mg/L)	Flucytosine MIC (mg/L)	Amphotericin B MIC (mg/L)	Micafungin MIC (mg/L)	*ABC11-ABC1* region coverage	*ERG11* variants*	FKS1 variants*	Source of strain
Pkud	C-AR1	CBS 2046	Fingernail pus	Argentina	**3n**	MATa/alpha	23	64	0.5	2	0.25	extra copy of chr. 4	A15V (het) R293K (het) S393L (het)	Q23H (het) S274N (hom) L701M (hom)	Westerdijk Institute
Pkud	C-BR1	CBS 5146	Faeces	Brazil	**3n**	MATa/alpha	44	16	2	2	0.125	low	—	S274N (het) L701M (hom)	Westerdijk Institute
Pkud	C-CN1	CK1	Secretions	China	2n	**MATa**	43	32	2	2	0.03125	—	—	L701M (hom)	Mingfeng Zhao [57]
Pkud	C-CN2	CK2	Sputum	China	2n	MATa/alpha	43	32	2	2	0.0625	—	A15V (hom)	S274N (het) L701M (het)	Mingfeng Zhao [57]
Pkud	C-CN3	CK4	Urine	China	**3n**	MATa/alpha	49	64	1	2	0.03125	extra copy of chr. 4	—	S274N (het) L701M (hom)	Mingfeng Zhao [57]
Pkud	C-CN4	CK16	Urine	China	**3n**	MATa/alpha	47	32	1	2	0.03125	—	—	S274N (het) L701M (hom)	Mingfeng Zhao [57]
Pkud	C-FI1	05BV00323	Blood	Finland	2n	MATa/alpha	43	64	2	1	0.03125	—	—	S274N (het) L701M (hom)	Timo Hautala [55]
Pkud	C-FI2	05BV00341	Pharynx	Finland	2n	MATa/alpha	52	64	2	1	0.25	—	—	S274N (het) L701M (hom)	Timo Hautala [55]
Pkud	C-FI3	05BV00147	Rectum	Finland	2n	MATa/alpha	48	64	2	1	0.25	—	—	S274N (het) L701M (hom)	Timo Hautala [55]
Pkud	C-FR2	CNRMA12.1278	Blood	France	2n	MATa/alpha	22	32	2	1	0.03125	—	—	S274N (het) L701M (het)	Francoise Dromer [29]
Pkud	C-IE1	60155	Blood	Ireland	2n	**MATa**	47	32	4	2	0.03125	high	—	L701M (hom)	Tom Rogers (TCD)
Pkud	C-IE2	17950	Blood	Ireland	2n	MATa/alpha	46	32	4	2	0.03125	low	A15V (het)	L701M (hom)	Tom Rogers (TCD)
Pkud	C-IE3	32050	Blood	Ireland	2n	MATa/alpha	51	64	2	1	0.125	—	—	S274N (hom) V319I (het) L701M (hom)	Tom Rogers (TCD)
Pkud	C-IE4	33769	Blood	Ireland	2n	MATa/alpha	44	32	8	1	0.125	low	A15V (het)	L701M (hom)	Tom Rogers (TCD)
Pkud	C-IE5	91020	Urine	Ireland	2n	MATa/alpha	44	16	1	1	0.03125	—	—	S274N (hom) L701M (hom)	Tom Rogers (TCD)
Pkud	C-IE6	100672	Urine	Ireland	2n	MATa/alpha	50	128	2	2	0.03125	—	—	S274N (het) L701M (hom)	Tom Rogers (TCD)
Pkud	C-IT1	CBS 2052	Sputum	Italy	2n	MATa/alpha	25	32	1	2	0.0625	low	A15V (het) R293K (het) S393L (het)	S274N (het) L701M (hom)	Westerdijk Institute
Pkud	C-IT2	CK-2	Vaginal	Italy	2n	MATa/alpha	50	32	1	1	0.03125	—	—	S274N (het) L701M (hom)	Barbara Skerlavaj [56]
Pkud	C-IT3	CK-3	Vaginal	Italy	2n	MATa/alpha	49	32	1	2	0.03125	—	—	L701M (hom)	Barbara Skerlavaj [56]
Pkud	C-LK1	CBS 573 T	Sputum	Sri Lanka	2n	MATa/alpha	34†	32	2	2	0.125	—	—	—	Westerdijk Institute
Pkud	E-FI4	CBS 5590	Baking	Finland	2n	MATa/alpha	45	16	0.5	1	0.03125	low	A15V (het) G187S (het)	L701M (het)	Westerdijk Institute
Pkud	E-GH1	CBS 2048	Fermenting cacao	Ghana	2n	MATa/alpha	48	32	4	2	0.25	—	—	S274N (het) L701M (hom)	Westerdijk Institute
Pkud	E-HU1	CBS 5687	Baking	Hungary	2n	MATa/alpha	10	64	1	2	0.03125	—	—	S274N (het) L701M (hom)	Westerdijk Institute
Pkud	E-JP1	CBS 6520	Domestic sewage	Japan	2n	**MATalpha**	45	32	4	4	0.03125	—	—	S274N (het) L701M (hom)	Westerdijk Institute
Pkud	E-JP2	CBS 2069	Homare miso	Japan	2n	**MATa**	21	32	1	2	0.03125	—	A15V (hom)	Q23H (hom) S274N (hom) L701M (hom)	Westerdijk Institute
Pkud	E-JP3	CBS 6799	Cabbage refuse	Japan	2n	MATa/alpha	19	32	1	4	0.0625	—	—	S274N (het) L701M (hom)	Westerdijk Institute
Pkud	E-JP4	CBS 7322	Soil	Japan	2n	MATa/alpha	44	32	8	4	0.03125	—	—	S274N (het) L701M (hom)	Westerdijk Institute
Pkud	E-PL1	CBS 8249	Fermentation vat in citric acid factory	Poland	**3n**	MATa/alpha	48	32	2	4	0.03125	low	—	S274N (het) L701M (hom) I1405V (het)	Westerdijk Institute
Pkud	E-RU1	CBS 5147 T	Fruit juice	Russia	**3n**	MATa/alpha	35†	8	1	4	0.0625	low	D226N (het)	S274N (het) L701M (hom)	Westerdijk Institute
Pkud	E-UK1	CBS 2062	Silage	United Kingdom	2n	MATa/alpha	52	32	2	2	0.125	—	—	S274N (het) L701M (hom)	Westerdijk Institute
Pkud	E-US1	CBS 2065	Pickles	USA	2n	MATa/alpha	46	64	4	4	0.0625	—	A15V (hom)	S274N (het) L701M (hom)	Westerdijk Institute
Pkud	E-WI1	CBS 2054	Fermenting cacao	West Indies	**3n**	MATa/alpha	48	16	1	1	0.125	low	—	S274N (het) L701M (hom)	Westerdijk Institute
Pferm	Pferm-PL1	fo/MP/02	Pickled cucumber	Poland	N.D.	MATa/alpha	17	64	0.5	0.5	0.125	—	—	—	Katarzyna Rajkowska [45]
Pferm	Pferm-PL2	fo/BM/02	Pickled cucumber	Poland	N.D.	N.D.	n/a	64	0.5	2	0.03125	—	—	—	Katarzyna Rajkowska [45]
Pnorv	Pnorv-NO1	CBS 1922 T	Sputum	Norway	N.D.	MATalpha	16	N.D.	N.D.	N.D.	N.D.	—	—	—	Westerdijk Institute
Cpara	CLIB214	CLIB214	n/a	n/a	2n	n/a	n/a	1	0.125	2	0.5	—	—	—	Geraldine Butler (UCD)

* Only nonsynonymous variants are listed. Hom and Het indicate homozygous and heterozygous genotypes relative to the CBS573 reference sequence.

^†^ Genomes also sequenced by PacBio.

**Fig 4:**
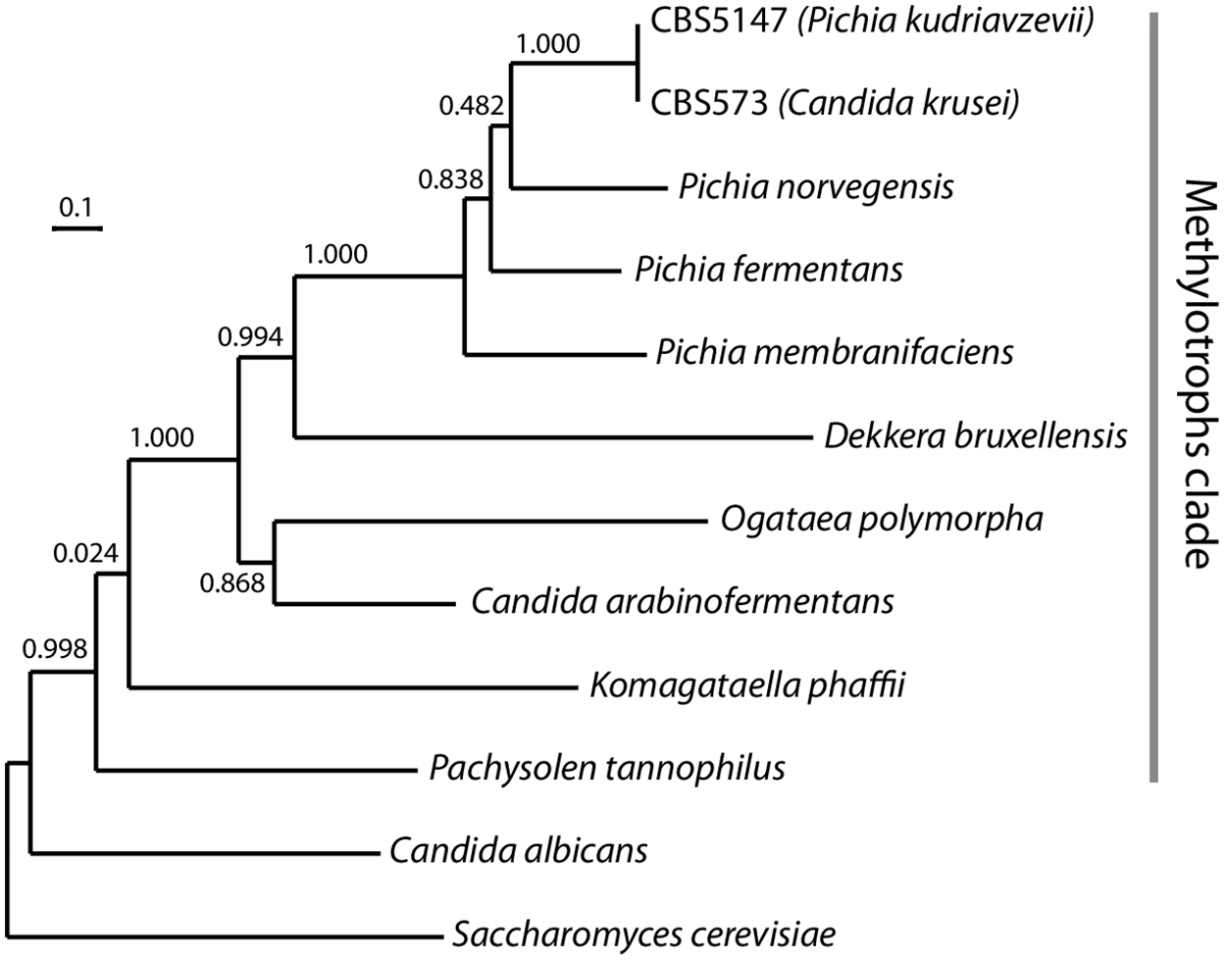
Phylogenetic position of *P*. *kudriavzevii*. The tree was constructed from Mdn1 protein sequences (aligned with MAFFT v7.0 [77]) using PhyML v3.1 [78]. Branch supports represent bootstrap values.

We constructed a phylogenetic tree using the Mdn1 protein. *MDN1* is the largest gene in budding yeast genomes and codes for a protein of almost 5000 amino acids that functions as a ribosome assembly factor. It is a convenient phylogenetic marker because the protein is large, non-repetitive and has a low rate of insertions/deletions. The tree (Fig 4) confirms that *P*. *norvegensis* is close to *P*. *kudriavzevii/C*. *krusei* (the Mdn1 proteins of CBS573 and CBS5147 are identical), with *P*. *fermentans* and *P*. *membranifaciens* more distantly related. It also confirms that *P*. *kudriavzevii/C*. *krusei* lies in the methylotrophic yeasts clade and is only distantly related to *C*. *albicans*.

### Genetic diversity in clinical and environmental strains

In this section, we describe analysis of genetic diversity in a set of 32 strains. We show that all strains are diploid or triploid, that losses of heterozygosity are common, and we examine the phylogenetic relationships among strains.

#### Illumina sequence data

To survey genetic diversity, and in particular to address the question of whether clinical (‘*C*. *krusei*’) and environmental (‘*P*. *kudriavzevii*’) isolates form separate clades, we chose an additional 30 strains for Illumina sequencing. The strains came from a wide range of countries and isolation sources (Table 2). Strains were named using a prefix C- or E- to identify clinical or environmental origin respectively, followed by a two-letter country code and a digit. We refer to the non-clinical strains as environmental isolates, but this category is heterogeneous and includes strains used for the production of fermented foods, strains that were isolated as food contaminants, and strains isolated from organic sources such as soil, silage and sewage (Table 2). Including the two type strains, our dataset consists of 20 clinical isolates and 12 environmental isolates.

#### Loss of heterozygosity

We mapped the Illumina reads from each strain to the CBS573 reference genome using BWA [46] and identified variant sites using the GATK SNP (single nucleotide polymorphism) calling pipeline. Relative to the CBS573 reference, the mean density of SNPs (after filtering, see Methods) was 3.95 SNPs/kb, and the range among strains was 2.28 to 5.08 SNPs/kb (S2 Table). This level of SNP diversity is comparable to *C*. *albicans* and slightly below that seen in *C*. *glabrata* [47, 48].

**Fig 5:**
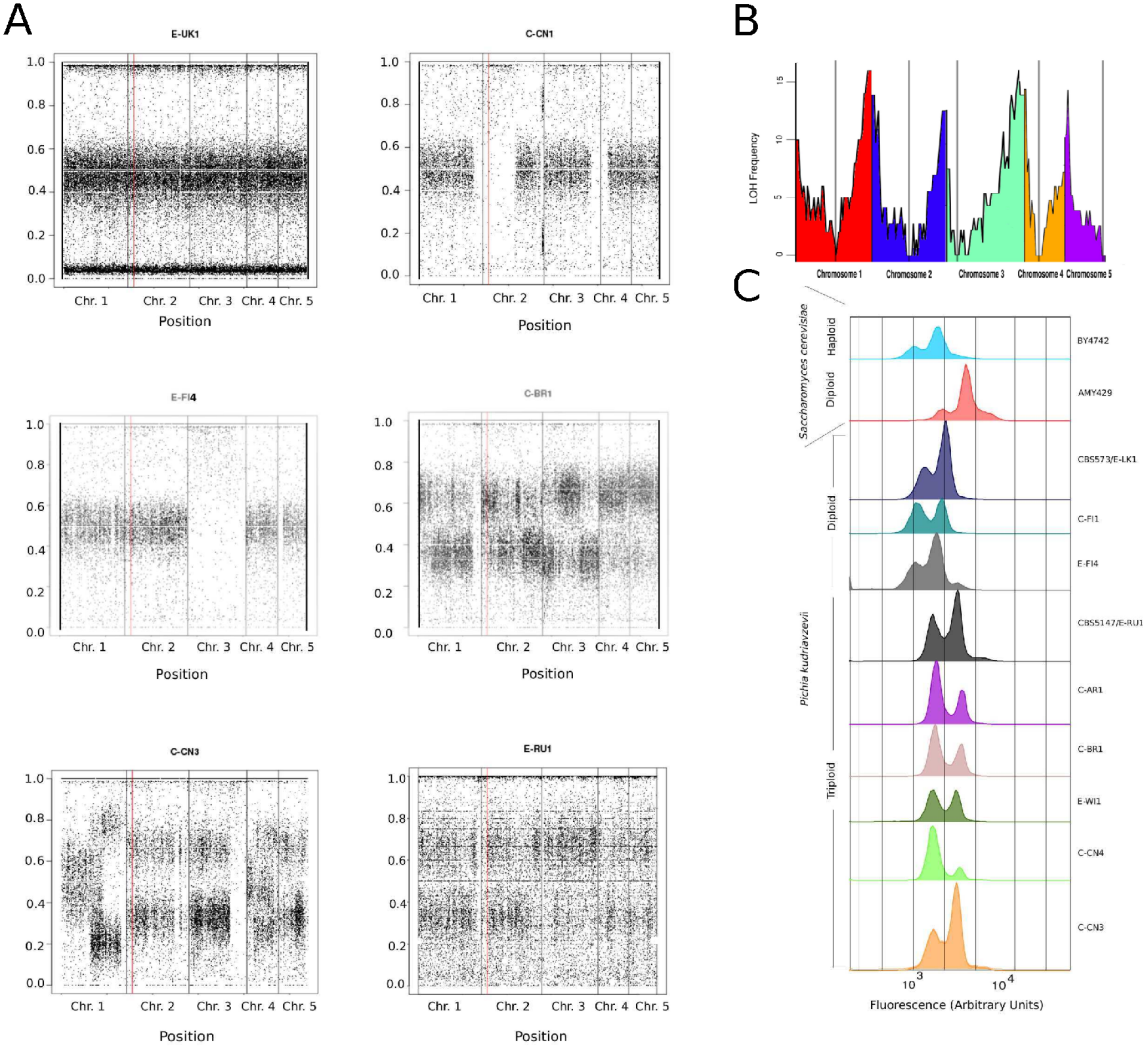
Ploidy variation and losses of heterozygosity in *P*. *kudriavzevii*. (A) Examples of allele frequency plots for non-reference alleles in six strains, showing a heterozygous diploid strain (E-UK1), diploid strains with heterozygosity and LOH (C-CN1, E-FI4), triploid strains (C-BR1, E-RU1), and a strain with partial aneuploidy (C-CN3). The X-axis in each plot is chromosomal coordinates through the genome, and the Y-axis is the frequency of the non-reference base at each polymorphic site. Red vertical lines show the position of the *MAT* locus. (B) Distribution of loss-of-heterozygosity regions. For each 50-kb window in the genome, the Y-axis shows the number of strains (out of 32) that were scored as showing LOH in the window. Colors indicate different chromosomes, and vertical lines mark centromere positions. (C) DNA content measured by flow cytometry of propidium iodide stained cells, in 9 *P*. *kudriavzevii* strains, with *S*. *cerevisiae* haploid (BY4742) and diploid (AMY429) controls. Approximate genome sizes (excluding rDNA) are 12 and 24 Mb for haploid and diploid *S*. *cerevisiae*, and 22 and 33 Mb for diploid and triploid *P*. *kudriavzevii*.

For each strain, we plotted the frequency of the non-reference allele at each variable site along the genome (Fig 5A; plots and allele frequency histograms for all 32 strains are shown in S1 File). In most strains, for example strain E-UK1, allele frequencies cluster around 0.5. This pattern indicates that E-UK1 is diploid and heterozygous throughout its genome. Some strains show losses of heterozygosity (LOH) in parts of the genome, for example strains C-CN1 and E-FI4 (Fig 5A). LOH was previously reported in another clinical isolate [23]. We defined LOH regions as 50-kb genomic windows containing fewer than 30 heterozygous SNPs (see Methods). LOH is quite frequent: 30 of the 32 strains contained at least one 50-kb LOH region. LOH can span a whole chromosome, such as chromosome 3 of E-FI4, but more commonly it affects a section of a chromosome extending to the telomere (chr. 1R, 2L, 3R, 4L in C-CN1, and chr. 5L in E-FI4). We plotted the number of strains that have lost heterozygosity at each region across the whole genome, and found that LOH is most frequent towards the telomeres, and least frequent at the centromeres (Fig 5B). This pattern is similar to the pattern seen in an analysis of 1,011 *S*. *cerevisiae* genomes [49], and strongly suggests that LOH in *P*. *kudriavzevii* is caused by break induced replication [50].

Most of the strains are *MAT***a**/α heterozygotes at the *MAT* locus (marked by the red line in Fig 5A), but in four strains including C-CN1 the *MAT* locus was affected by LOH, leaving only one allele type (Table 2). These losses of *MAT* alleles were confirmed by BLAST searches against *de novo* assemblies of the Illumina data for each strain: most strains contained both *MAT***a** and *MAT*α gene sequences, but three contained only *MAT***a**, and one contained only *MAT*α (Table 2).

#### Ploidy variation

Most of the 32 strains show SNP allele frequencies centered on 0.5 indicating that they are diploid, but for seven strains the allele frequencies cluster around 0.33 and 0.66 which suggests triploidy. None of the strains are haploid. The triploid strains include C-BR1 and E-RU1 (CBS5147), the type strain of *P*. *kudriavzevii* (Fig 5A). In C-BR1, the allele frequencies occur in a pattern of alternating 2:1 and 1:2 ratios between non-reference and reference alleles, which is possibly the result of homogenization between different pairs of chromosomes in different regions of the triploid genome (some regions of the genome have genotype AAB, and other regions ABB, where A denotes reference alleles and B denotes non-reference alleles). To validate our ploidy assignments, we used flow cytometry of cells stained with propidium iodide, with haploid and diploid *S*. *cerevisiae* strains as controls. This experiment confirmed the ploidy assignments based on SNP allele frequencies, for all the strains that we tested—six triploids and three diploids (Fig 5C). Ploidy data is summarized in Table 2. The proportion of triploid strains (22%) is double the proportion reported in wild isolates and clinical isolates of *S*. *cerevisiae* [51, 52].

Some strains show evidence of aneuploidy. The SNP allele frequency patterns indicate that strain C-CN3 is triploid in most regions of its genome (Fig 5A), in agreement with its flow cytometry profile (Fig 5B). However, some parts of its genome show allele frequencies consistent with two or four copies (chrs. 1L, 4L) or five copies (chr. 1R). Similarly, strain C-IT2 is diploid in most of its genome but appears to have 3 copies of chromosome 1L (S1 File). For strain C-AR1, which is triploid for most of the genome, the right-hand three-quarters of chromosome 4 has coverage 1.32 times the other chromosomes, indicating that there are four copies of this region (S1 File). Because these regions with altered copy number are not complete chromosomes, these strains may therefore contain extra copies of rearranged chromosomes.

#### Phylogenetic relationship among strains

**Fig 6:**
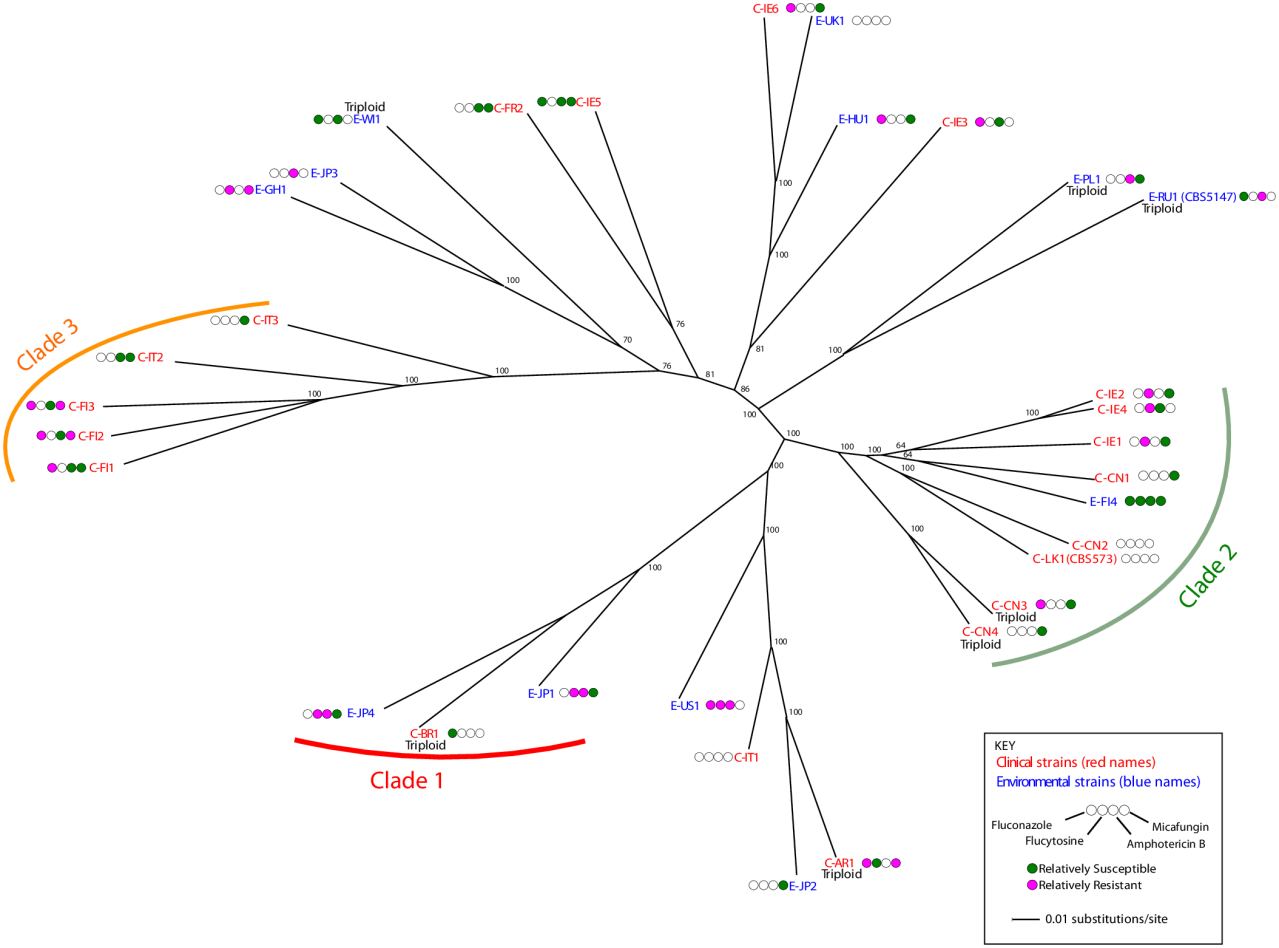
Population structure of clinical and environmental strains. A phylogenetic tree of strains was constructed from data from a filtered set of 150,306 SNP sites, using RRHS and Maximum Likelihood (see Methods). Branch supports represent pseudo-bootstrap values. Strains named in red are clinical isolates, and strains named in blue are environmental. For each strain, four circles indicate relative resistance (magenta) or relative sensitivity (green) to four drugs as shown in the key.

To investigate the relationship among strains, we constructed a phylogenetic tree from whole-genome SNP data using IQ-TREE [53]. The tree (Fig 6) shows no clear separation between clinical and environmental isolates, and relatively little phylogenetic structure of any kind. It shows a continuum of relationships, without any groups of very closely related strains, and without deep divisions between clades. Analysis using the program STRUCTURE [54] (S3 Fig) suggested that the optimal subdivision of the strains is into four populations, three of which form monophyletic clades (Clades 1–3 in Fig 6). Clade 3 contains only clinical isolates, and all but one of the isolates in Clade 2 is clinical. However, many clinical isolates such as C-AR1 and C-IE6 have environmental strains as their closest relatives. The simplest explanation of the phylogeny is that there have been multiple transmissions between environmental and clinical habitats.

Nevertheless, the tree does show some evidence of possible human-to-human transmission. Three strains isolated from three patients in a hospital in Finland in the same year cluster together (C-FI1, C-FI2, C-FI3) [55]. Two isolates from outpatients at a hospital in Italy also lie close together (C-IT2, C-IT3) [56]. Two strains from a hospital in Ireland form the closest pair in the tree (C-IE2, C-IE4) and group weakly with a third (C-IE1), but three other strains from the same hospital are scattered across the tree. All four clinical isolates from Tianjin, China [57] lie in Clade 2, and among these, two strains form a close pair (C-CN3, C-CN4) and are both triploid. The only sample from soil in our dataset, E-JP4, groups with samples from faeces (C-BR1) and sewage (E-JP1) to form Clade 1, indicating possible clustering by habitat.

### Variation in drug resistance

We assayed the *in vitro* sensitivity of all the sequenced strains to four antifungal drugs—fluconazole, flucytosine, amphotericin B, and micafungin—using the EUCAST protocol [58]. We also included two *P*. *fermentans* strains in the drug assays, one of which (Pferm-PL1) was sequenced and the other (Pferm-PL2) was not. The observed MICs for each strain in each drug are presented in Table 2. The distribution of MICs for our *P*. *kudriavzevii* strains in fluconazole and micafungin were in line with previous distributions reported for *C*. *krusei*, while those for amphotericin B were about 2 dilution points higher (www.eucast.org, Rationale documents for clinical breakpoints). No EUCAST ranges exist for flucytosine, but our values were similar to ranges reported in previous studies [59].

We wanted to focus on variation within *P*. *kudriavzevii* in its relative levels of drug resistance or sensitivity, so we plotted the distribution of MIC values among strains (S4 Fig), and chose cutoffs that define groups that are ‘relatively resistant’ (RR) or ‘relatively sensitive’ (RS) to each drug, within the observed distribution. The strains designated as RR and RS for each drug are highlighted in magenta and green, respectively, in Table 2. The phylogenetic distribution of these RR and RS strains is shown on the tree in Fig 6.

#### Fluconazole

As expected, all strains of *P*. *kudriavzevii* were resistant to fluconazole (MIC ≥ 8 mg/L), whereas the *C*. *parapsilosis* reference strain was sensitive (MIC 1 mg/L). Within the range of resistance shown by *P*. *kudriavzevii*, we defined RS strains as those with MIC ≤ 16 mg/L fluconazole, and RR strains as those with MIC ≥ 64 mg/L (Table 2; S4A Fig). The RR group included seven clinical isolates, confirming previous reports of fluconazole resistance for some of these isolates (C-FI1, C-FI2, C-FI3, C-CN3) [55, 57]. However, the RR group also included two environmental isolates (baking strain E-HU1, and E-US1 from pickles) with fluconazole MICs of 64 mg/L. The two *P*. *fermentans* strains, isolated from pickled cucumber brine [45], both also had high MICs (64 mg/L). Many of the RR strains of *P*. *kudriavzevii* showed slower growth rates than RS strains at low fluconazole concentrations (S4A Fig).

Previous studies on azole resistance in *P*. *kudriavzevii* have identified roles for two loci, *ERG11* and *ABC11-ABC1*, that are only 60 kb apart on chromosome 4 [26, 27, 57]. Erg11 is an enzyme in the ergosterol synthesis pathway (lanosterol 14-α-demethylase) that is the target of azole drugs. The Erg11 enzyme of *P*. *kudriavzevii* has unusually low affinity for fluconazole, providing a partial explanation for the drug resistance of this species [27]. In our data, we noted five nonsynonymous polymorphisms in Erg11 (Table 2), but none of them correlated with variation in fluconazole phenotypes among strains.

*ABC11* and *ABC1* are genes for drug efflux pumps in the ABC transporter family. *ABC11* is located immediately upstream of *ABC1*, and they have 98% DNA sequence identity as a result of recurrent gene conversion [26]. While most strains of *P*. *kudriavzevii* contain genes *ABC11* and *ABC1* in tandem, ectopic recombination between *ABC11* and *ABC1* can produce arrays that have contracted to one gene (an *ABC11-1* chimera) or expanded to three genes [26]. Our PacBio assembly of the CBS573 genome contains a tandem *ABC11-ABC1* gene pair, but CBS5147 was heterozygous at this locus and yielded two PacBio contigs: one with a contracted *ABC11-1* chimera, and the other with a tandem *ABC11-ABC1* pair. In our Illumina data, we identified seven other strains that had noticeably lower sequence coverage at the *ABC11-ABC1* locus than in neighboring regions of chromosome 4 (Table 2). Because these seven strains all retain a copy of the 662 bp intergenic region between *ABC11* and *ABC1* (it is present in *de novo* assemblies of these genomes), which is absent in *ABC11-1* chimeras [26], our interpretation of these data is that, like CBS5147, the seven strains are heterozygotes with an *ABC11-1* chimeric gene on one chromosome, and a normal tandem *ABC11-ABC1* gene pair on another.

Importantly, the group of eight strains with *ABC11-1* chimeric genes includes 4 of the 5 strains that are RS for fluconazole, and none of the RR strains (Table 2). This distribution is significantly different from the null expectation (*P* = 0.0007 by two-tailed Fisher’s exact test of the hypothesis that the two coverage categories have the same distribution of RR, intermediate, and RS strains). Thus, collapse of the *ABC11-ABC1* tandem gene pair to form a single *ABC11-1* chimeric gene correlates with a reduced level of fluconazole resistance in *P*. *kudriavzevii*.

#### Flucytosine

For flucytosine, we defined RS strains as those with MIC ≤ 0.5 mg/L flucytosine, and RR strains as those with MIC ≥ 4 mg/L (Table 2; S4B Fig). All *P*. *kudriavzevii* were again more resistant than the *C*. *parapsilosis* CLIB214^T^ reference strain. The two *P*. *fermentans* strains were somewhat less resistant than *P*. *kudriavzevii*. Among the seven relatively resistant strains, four were environmental isolates (E-GH1, E-JP1, E-JP4, E-US1).

#### Amphotericin B

Our assays in amphotericin B gave consistently higher MIC values than expected under the EUCAST protocol, for unknown reasons (Table 2; S4C Fig). Although this difference means that the absolute values of our MICs for amphotericin B cannot be compared to the EUCAST breakpoints, it is still valid to compare relative values within our study, to identify RR and RS strains. We defined RS strains as those with MIC ≤ 1 mg/L, and RR strains as those with MIC ≥ 4 mg/L, in our assays. Surprisingly, all six RR strains were environmental isolates. All but one of the clinical isolates in Clade 3 were RS for amphotericin B, while those in Clades 1 and 2 were not.

#### Micafungin

Micafungin is the recommended most effective existing treatment for *P*. *kudriavzevii* [60]. All *P*. *kudriavzevii* strains were more sensitive to micafungin than the *C*. *parapsilosis* reference strain. We defined RS strains as those with MIC ≤ 0.03 mg/L micafungin, and RR strains as those with MIC ≥ 0.25 mg/L (Table 2; S4D Fig). One of the four RR strains was an environmental isolate (E-GH1). None of the 32 strains contained mutations at amino acid positions 655–662 of the glucan synthase gene *FKS1*, which have previously been implicated in echinocandin resistance in this species [28, 29]. Alleles with S274N and L701M substitutions relative to the CBS573 reference sequence of *FKS1* occur at high frequency (Table 2).

Across all four drugs, environmental isolates were as likely as clinical isolates to show relative resistance (RR) to a drug. Of the 32 RR designations (magenta circles in Fig 6), 14 are in the 12 environmental isolates, and 18 are in the 20 clinical isolates (not significantly different; *P* = 0.41 by two-tailed Fisher’s exact test). One environmental strain, E-US1, was RR to three drugs (fluconazole, flucytosine, and amphotericin B).

## Discussion

Our results confirm that *P*. *kudriavzevii* and *C*. *krusei* are the same species and demonstrate that their genomes are collinear. The discovery that clinical and environmental isolates are interspersed in a phylogenetic tree of strains and do not form distinct clades indicates that there is no justification for continuing to use both names for this species. A third name, *I*. *orientalis*, is obsolete, having been formally replaced by the name *P*. *kudriavzevii* [6]. Furthermore, we found that the species has a fourth name, *Candida glycerinogenes*. Since its discovery by Zhuge in 1973, ‘*C*. *glycerinogenes*’ has been used in China for the industrial-scale production of glycerol by fermentation of plant carbohydrates [19]. Extensive research has been carried out into its osmotolerance, and genetic manipulation methods have been developed (e.g., [61, 62]). We find that 37 of the 38 *C*. *glycerinogenes* gene sequences available in NCBI are virtually identical to *P*. *kudriavzevii* sequences, including the 18S rDNA. The existence of multiple names for this species has almost certainly impeded research into it. In keeping with the One Fungus One Name principle [63], we suggest that *P*. *kudriavzevii* should be the only name used in future.

One of the most unexpected features of the genome is the structure of its centromeres, which consist of a simple but large IR. The 99% DNA sequence identity of the 8–14 kb units that form the IRs means that centromere organization would have been difficult to deduce without long-read PacBio data. The structure of the centromeres most closely resembles those of *Komagataella phaffii*, another yeast in the methylotrophs clade. However, the *K*. *phaffii* centromeres are much smaller, consisting of just a 2-kb IR on each chromosome with a 1-kb central region [40]. The only other yeasts in this clade whose centromeres have been characterized are *Ogataea polymorpha*, whose centromeres contain clusters of Ty5-like retrotransposons and do not seem to have an IR structure, and *Kuraishia capsulata* which has been reported to have point centromeres [40, 64]. The *P*. *kudriavzevii* genome does not contain any Ty5-like elements. Its centromeres do contain pseudogenes of Ty3-like elements, and these are more abundant at the centromeres than elsewhere in the genome, but the only intact Ty3-like elements are not centromeric. Centromeres with similar IR structures also occur outside the family Pichiaceae, in *C*. *tropicalis* (family Debaryomycetaceae) [41] and *Schizosaccharomyces pombe* (subphylum Taphrinomycotina) [65]. The centromeres of *Sch*. *pombe* chromosomes 1 and 2 are similar in size and organization to those of *P*. *kudriavzevii*, whereas its third centromere is larger and more complex [40].

An important remaining question concerns the sexual cycle. When *P*. *kudriavzevii* was first described, it was reported to be able to sporulate, forming one spore per ascus [7]. Later studies by Kurtzman and colleagues reported that the type strain of *P*. *kudriavzevii* does not mate or sporulate [8, 9]. Our discovery that this strain is triploid provides a possible explanation for its failure to sporulate, or at least its failure to produce viable spores. It will be of interest to re-investigate the question of sporulation using strains that are diploid *MAT***a**/α heterozygotes. Of the 32 strains we studied, 20 have this status (Table 2). It will also be of interest to test if mating can be induced between strains with *MAT*α/α and *MAT***a**/**a** genotypes. The genome of CBS573 appears to contain a complete repertoire of sexual cycle genes, including pheromone genes (*MF***a**, *MF*α) and orthologs of most of the genes in the MAPK kinase pathway that controls mating in *S*. *cerevisiae*. It also contains orthologs of many genes involved in meiosis, although *IME1*, the master inducer of meiosis, has not been found in *P*. *kudriavzevii* nor any other species outside the family Saccharomycetaceae [66]. A possible explanation for the triploid isolates is that, for example, a *MAT***a**/α diploid underwent loss of heterozygosity to become *MAT*α/α, and then mated with a *MAT***a** haploid spore to form a *MAT***a**/α/α triploid.

Because clinical isolates were found to be closely related to environmental isolates, either infections are being acquired opportunistically from the environment, or yeast strains from infected humans are colonizing the environment. In view of the range of sources, the former possibility is more likely. The use of *P*. *kudriavzevii* in biotechnology therefore presents a potential hazard to the health of immunocompromised workers, and potentially also to consumers [67]. Moreover, high resistance to fluconazole is common in environmental isolates. The resistance to fluconazole is shared with *P*. *fermentans* (Table 2), *P*. *norvegensis* and other *P*. *cactophila* clade species [28, 34] and therefore seems to be a trait of the whole genus *Pichia*. *C*. *krusei* and *P*. *kudriavzevii* are both categorized as Biosafety Level 1 (BSL-1), which is the lowest level of precaution. In another case of a pathogen with a major biotechnological role, it was suggested that a harmless closely related species should be used as a replacement [68]. Similarly, it may be advisable to consider non-pathogenic *Pichia* species as possible alternatives for some industrial applications. It would also be advisable to set limits on the levels of drug resistance permissible in *P*. *kudriavzevii* strains that are used in industry, particularly the food industry.

## Methods

### Yeast strains, DNA and RNA preparation, and sequencing

Yeast strains were obtained from the laboratories and culture collections listed in Table 2. For clinical isolates obtained from hospital laboratories, all isolates came from different patients and where possible we obtained isolates that were taken prior to drug treatment. High molecular weight DNA for PacBio sequencing was purified using Qiagen Puregene Yeast/Bacterial Kit B. DNA for Illumina sequencing was harvested from stationary-phase cultures by homogenization with glass beads followed by phenol-chloroform extraction and ethanol precipitation. Purified DNA was concentrated with the Genomic DNA Clean & Concentrator-10 (Zymo Research, catalog D4010). Isolation of mRNA for RNAseq was done using the MasterPure Yeast RNA Purification kit (Epicentre, Madison, WI, USA) from CBS573 cultures grown to early log phase (OD_600_ ~ 1) in YPD media at 30°C. PacBio DNA sequencing of strains CBS573 and CBS5147 was done at the Earlham Institute, UK, with 4 SMRT cells per strain. Illumina sequencing of these two strains was also done at the Earlham Institute using Low Input Transposon Enabled (LITE) libraries. Illumina sequencing of all other strains was done by the core facility of the University of Missouri, USA, using TruSeq libraries (coverage details are given in Table 2). Illumina RNA-seq (30 million reads) of CBS573 was done in-house at University College Dublin.

### Sequence assembly

Assembly of the CBS573 PacBio data using HGAP3 software initially produced seven nuclear contigs (115x coverage) and the mitochondrial genome. Overlaps between the ends of two pairs of contigs were merged manually to obtain five near-complete chromosome sequences. Illumina sequencing of the same strain (35x coverage) was then used to error-correct the chromosome sequences, in particular to remove insertion/deletion errors in homopolymer tracts. Error correction was done using Pilon [69] and manual comparison to *de novo* Illumina contigs assembled by SPAdes version 3.10 [70]. Assembly of the CBS5147 PacBio data using HGAP3 yielded 13 contigs (94x coverage). This assembly appeared to have a higher level of indel errors than the CBS573 assembly, so we used CBS573 as the reference genome sequence for annotation and downstream analyses. *De novo* assemblies of the other 30 strains were made using SPAdes version 3.10 [70]. Nucleotide sequence identity of 99.6% between the reference chromosome sequences of CBS573 and CBS5147 was calculated from a MUMmer (v3.23) alignment of the whole genome [37].

### Ploidy estimation by propidium iodide staining

Ploidy was estimated with a modified version of the method of Popolo *et al*. [71]. Briefly, aliquots of exponentially growing cells in YM medium (3 g/L yeast extract, 3 g/L malt extract, 5 g/L peptone and 10g/L dextrose), were adjusted to OD_600_ = 1 in 1 mL sterile ice-cold water, centrifuged (5 min, 5000 rpm) and fixed in 1 mL cold 70% ethanol for 24 hours at 4 °C. Fixed cells were next treated with 100 μL 1 mg/mL RNAse A for 90 min at 37 °C after centrifugation to remove the ethanol. RNase A treated cells were centrifuged and the pellet stained with 100 μL 0.05 mg/mL propidium iodide at 4 °C for 24 hours. Fifty μL of stained cells was diluted to 500 μL with ice-cold water, filtered with 50 μm celltrics filters (Sysmex, UK) and run on a BD Accuri C6 flow cytometer. Data were analysed using Flowjo software (Flowjo, LLC).

### SNP analysis

BAM alignments of Illumina reads from each strain to the CBS573 reference genome were generated using the Burrows-Wheeler Aligner (BWA) with default parameters [46]. Unmapped reads were removed using SAMtools [72] and headers were added using the AddOrReplaceReadGroups program in Picard Tools [http://picard.sourceforge.net]. Variants against the reference were called with the GATK HaplotypeCaller tool in DISCOVERY genotyping mode with the following parameters: “-stand_emit_conf 10 -stand_call_conf 30—emitRefConfidence GVCF” [73]. The resulting set of 32 GVCF files defined an initial set of 169,789 SNP sites that were variable among the 32 strains, and this set was used for ploidy analyses (Fig 5A).

To analyze patterns of LOH, we divided the genome into consecutive 50-kb windows and calculated the number of heterozygous SNPs in each window (with allele frequencies between 0.15 and 0.85, from the initial set of 169,789 sites). Windows containing <30 heterozygous sites were categorized as showing LOH (Fig 5B; S2 Table). The threshold of 30 heterozygous sites was chosen because it is a local minimum in the distribution of heterozygous SNP numbers among all windows in all strains.

For phylogenetic and STRUCTURE analyses (Fig 6; S3 Fig), we first filtered the 32 GVCF files to remove low allele frequency sites (allele frequency <0.15). The filtered GVCFs were then jointly genotyped with the GenotypeGVCFs function of GATK to produce a single multisample SNP file containing data on every strain. This filtered dataset contained 150,306 variable sites. For phylogenetic analysis of SNP data, we used the program RRHS [74] to preserve the impact of heterozygous SNPs. This program generated 100 datasets in which, for each heterozygous site in each strain, one allele was chosen randomly. Each of the 100 datasets was used to build a phylogenetic tree by maximum likelihood using IQ-TREE v1.6.5 [53], with option “-m GTR+ASC” to account for ascertainment bias. A single unrooted consensus tree was then constructed from these trees (Fig 6). STRUCTURE [54] uses SNP data to infer the population structure of the genomes in the dataset, assuming that the individuals are drawn from *k* populations. It also provides a value of estimated log likelihood (*ln Pr*) for the model used. We ran STRUCTURE (v2.3.4) for values of *k* between 2 and 8, and present the results for the value that gave the highest log likelihood, *k* = 4.

### Drug assays

Minimum Inhibitory Concentrations (MICs) of the four antifungal agents were determined using the EUCAST broth dilution method [58] with slight modifications. In brief, stock antifungal agents prepared with EUCAST recommended solvents were diluted to appropriate working concentrations in double strength RPMI-1640 2% G (RPMI-1640 supplemented with 2% w/v glucose). The working concentrations used were 128 mg/L for fluconazole and flucytosine, and 32 mg/L for amphotericin B and micafungin. A ten-series two-fold dilution starting with 200 mL working concentration of each agent was made row-wise in flat-bottomed 96-well plates using double strength RPMI-1640 2% G as diluent. Consequently, the wells of each dilution series yielded 100 mL of twice the recommended series of drug concentrations required for MIC determinations. The last two wells of each row containing an antifungal drug serial dilution were filled with 100 mL of drug-free RPMI-1640 2% G. Yeast inocula were prepared by growing three distinct colonies of each strain overnight on Sabouraud agar at 37°C and suspending them in sterile distilled water. To achieve final cell densities of 0.5–2.5 x 10^6^ cfu/ml in the microtitre wells as recommended, these suspensions were adjusted to OD_600_ 0.1 and then further diluted 1/10 in sterile water. Cell densities were confirmed by plate counting. Wells of each dilution series as well as the 11^th^ well containing drug-free RPMI-1640 2% G were inoculated with 100 mL of the prepared yeast suspensions. The last well was filled with 100 mL of sterile distilled water to serve as contaminant control. Inoculated plates were incubated without shaking at 37°C for 24 hours. Plates were read for OD_600_ values using a Spectramax 190 microplate reader (Molecular Devices, Sunnyvale, California, USA). As recommended in the EUCAST protocol [58], we calculated MIC_90_ for amphotericin B, and MIC_50_ for the other three drugs. One of the recommended control strains for yeasts in the EUCAST protocol is the type strain of *C*. *krusei* (CBS573^T^, synonymous with ATCC6258^T^), and we used the type strain of *C*. *parapsilosis* (CLIB214^T^) as a second control. MICs for the two control strains were within EUCAST guideline ranges, except for *C*. *krusei* CBS573^T^ in amphotericin B, which was 1 dilution point more resistant than the guideline.

### Accession numbers

The sequence data reported in this manuscript has been submitted to the NCBI nucleotide database with the following accession numbers: *P*. *kudriavzevii* CBS573 PacBio/Illumina annotated reference genome sequence (CP028773-CP028778); *P*. *kudriavzevii* CBS573 RNAseq Illumina reads (SRA accession SRP139056); *P*. *kudriavzevii* CBS573 *MAT*α allele region (MH260578); *P*. *kudriavzevii* CBS5147 PacBio genome sequence (CP028531-CP028535); Illumina genomic sequencing reads from 32 *P*. *kudriavzevii* strains (SRA accession SRP139299); *P*. *fermentans* strain fo/MP/02 (Pferm-PL1) Illumina WGS assembly (QAWB00000000); *P*. *norvegensis* strain CBS1922 (Pnorv-NO1) Illumina WGS assembly (QAWC00000000).

## Supporting information

S1 Fig Collinearity of CBS5147 and CBS573 chromosomes. Dot matrix plots compare PacBio assemblies of CBS5147 chromosomes (Y-axis) versus CBS573 chromosomes (X-axis). Black diagonals indicate matches in the same orientation, and red diagonals indicate matches in opposite orientations. Plots were constructed using DNAMAN (www.lynnon.com), with a criterion of 50 matches per 50-bp window. Bars at the top of the plots show the locations of annotated protein-coding genes in the CBS573 genome, with an absence of genes at the centromeres.
(TIF)

S2 Fig Complex relationship among *P*. *kudriavzevii* centromeres. 50-kb regions around the centromeres of the 5 chromosomes of CBS573 were concatenated and compared in a dot matrix plot. Black diagonals indicate matches in the same orientation, and red diagonals indicate matches in opposite orientations. Dashed lines mark the ends of the 50-kb section from each chromosome. The cyan grid marks the ends of the three sections of each centromere (*IRL*, left part of the IR; *MID*, middle region; *IRR*, right part of the IR). Locations of PkudTy3A pseudogenes (pink triangles) and PkudTy3B pseudogenes (blue triangles) are shown. The plot was constructed using DNAMAN (www.lynnon.com), with a criterion of 50 matches per 50-bp window.
(TIF)

S3 Fig Population structure analysis of *P*. *kudriavzevii* isolates. The diagram was built from a filtered dataset of 150,306 SNP sites using STRUCTURE [54] with *k* = 4. Each column represents a strain, and the colors represent the proportion of sites belonging to each of the 4 inferred populations. Populations 1–3 form monophyletic clades in the tree in Fig 6, but population 4 does not.
(TIF)

S4 Fig Drug resistance assays. Growth of strains after 24 hours (OD_600_) is plotted versus drug concentration for four drugs: (A) fluconazole, (B) flucytosine, (C) amphotericin B, and (D) micafungin. Histograms (insets) show the distribution of Minimum Inhibitory Concentration (MIC) values for all strains. For *P*. *kudriavzevii*, only strains designated as relatively resistant (red) or relatively sensitive (green) are identified in the keys; other strains are plotted as gray lines. Two strains of *P*. *fermentans* (blue) and the EUCAST control strains of *C*. *krusei* (CBS573^T^; black circles) and *C*. *parapsilosis* (CLIB214; black triangles) are also plotted. MIC is defined as the concentration required to inhibit 50% of growth in fluconazole, flucytosine and micafungin, and 90% in amphotericin B [58]. Each data point is the average of three biological replicates.
(TIF)

S1 Table Evaluation of genome annotation quality using BUSCO. Annotated protein datasets for *C*. *krusei* strain 81-B-5 [23], *I*. *orientalis* strain SD108 [10], and *P*. *kudriavzevii* strain 129 [21] were downloaded from the NCBI database. BUSCO version 3.0.2 (busco.ezlab.org) [36] was used to compare these annotations and our CBS573 annotation to two reference datasets of single-copy genes that are universally conserved in the Ascomycota lineage, or in the Saccharomycetales lineage. The BUSCO reports show the percentages of proteins in the reference datasets whose orthologs are complete (C), fragmented (F), or missing (M) in each annotation. Complete proteins are subdivided into those that are single-copy (S) or duplicated (D) in the annotations.
(DOCX)

S2 Table SNP densities and extent of loss-of-heterozygosity (LOH) regions in sequenced strains. (DOCX)

S1 File Allele frequencies and sequencing coverage in all 32 strains. For each strain, the plots show: (Top left) Allele frequency of non-reference alleles at polymorphic sites along the genome, as in Fig 5A. (Top right) Sequencing coverage along each chromosome. The red points are segmental means. (Lower 6 panels) Histograms of non-reference allele frequencies in the whole genome, and separately for each chromosome.
(PDF)

## References

[1] Cleveland AA, Harrison LH, Farley MM, Hollick R, Stein B, Chiller TM, et al Declining incidence of candidemia and the shifting epidemiology of *Candida* resistance in two US metropolitan areas, 2008–2013: results from population-based surveillance. PLoS One. 2015;10: e0120452 10.1371/journal.pone.0120452 25822249 PMC4378850

[2] Pfaller MA, Messer SA, Rhomberg PR, Castanheira M. CD101, a long-acting echinocandin, and comparator antifungal agents tested against a global collection of invasive fungal isolates in the SENTRY 2015 Antifungal Surveillance Program. Int J Antimicrob Agents. 2017;50: 352–358. 10.1016/j.ijantimicag.2017.03.028 28689871

[3] Angebault C, Djossou F, Abelanet S, Permal E, Ben Soltana M, Diancourt L, et al *Candida albicans* is not always the preferential yeast colonizing humans: a study in Wayampi Amerindians. J Infect Dis. 2013;208: 1705–1716. 10.1093/infdis/jit389 23904289

[4] Marenzoni ML, Morganti G, Moretta I, Crotti S, Agnetti F, Moretti A, et al Microbiological and parasitological survey of zoonotic agents in apparently healthy feral pigeons. Pol J Vet Sci. 2016;19: 309–315. 10.1515/pjvs-2016-0038 27487504

[5] Kurtzman CP, Smiley MJ, Johnson CJ. Emendation of the genus *Issatchenkia* Kudriavzev and comparison of species by deoxyribonucleic acid reassociation, mating reaction, and ascospore ultrastructure. Int J Syst Bacteriol. 1980;30: 503–513.

[6] Kurtzman CP, Robnett CJ, Basehoar-Powers E. Phylogenetic relationships among species of *Pichia*, *Issatchenkia* and *Williopsis* determined from multigene sequence analysis, and the proposal of *Barnettozyma* gen. nov., *Lindnera* gen. nov. and *Wickerhamomyces* gen. nov. FEMS Yeast Res. 2008;8: 939–954. 10.1111/j.1567-1364.2008.00419.x 18671746

[7] Kudrjawzew WI. Die Systematik der Hefen. Berlin: Akademie Verlag; 1960.

[8] Kurtzman CP, Smiley MJ. Heterothallism in *Pichia kudriavzevii* and *Pichia terricola*. Antonie Van Leeuwenhoek. 1976;42: 355–363. 1086649 10.1007/BF00394135

[9] Kurtzman CP. *Pichia* E.C. Hansen (1904) In: Kurtzman CP, Fell JW, Boekhout T, editors. The Yeasts, A Taxonomic Study. 2. Amsterdam: Elsevier; 2011 p. 685–707.

[10] Xiao H, Shao Z, Jiang Y, Dole S, Zhao H. Exploiting *Issatchenkia orientalis* SD108 for succinic acid production. Microb Cell Fact. 2014;13: 121 10.1186/s12934-014-0121-4 25159171 PMC4244060

[11] Miao Y, Xiong G, Li R, Wu Z, Zhang X, Weng P. Transcriptome profiling of *Issatchenkia orientalis* under ethanol stress. AMB Express. 2018;8: 39 10.1186/s13568-018-0568-5 29536208 PMC5849708

[12] Smukowski Heil C, Burton JN, Liachko I, Friedrich A, Hanson NA, Morris CL, et al Identification of a novel interspecific hybrid yeast from a metagenomic spontaneously inoculated beer sample using Hi-C. Yeast. 2018;35: 71–84. 10.1002/yea.3280 28892574 PMC5771821

[13] Bourdichon F, Casaregola S, Farrokh C, Frisvad JC, Gerds ML, Hammes WP, et al Food fermentations: microorganisms with technological beneficial use. Int J Food Microbiol. 2012;154: 87–97. 10.1016/j.ijfoodmicro.2011.12.030 22257932

[14] De Vuyst L, Harth H, Van Kerrebroeck S, Leroy F. Yeast diversity of sourdoughs and associated metabolic properties and functionalities. Int J Food Microbiol. 2016;239: 26–34. 10.1016/j.ijfoodmicro.2016.07.018 27470533

[15] Li P, Li S, Cheng L, Luo L. Analyzing the relation between the microbial diversity of DaQu and the turbidity spoilage of traditional Chinese vinegar. Appl Microbiol Biotechnol. 2014;98: 6073–6084. 10.1007/s00253-014-5697-4 24691870

[16] Chelliah R, Ramakrishnan SR, Prabhu PR, Antony U. Evaluation of antimicrobial activity and probiotic properties of wild-strain *Pichia kudriavzevii* isolated from frozen idli batter. Yeast. 2016;33: 385–401. 10.1002/yea.3181 27370793

[17] Radecka D, Mukherjee V, Mateo RQ, Stojiljkovic M, Foulquie-Moreno MR, Thevelein JM. Looking beyond *Saccharomyces*: the potential of non-conventional yeast species for desirable traits in bioethanol fermentation. FEMS Yeast Res. 2015; 10.1093/femsyr/fov053 26126524

[18] Mukherjee V, Radecka D, Aerts G, Verstrepen KJ, Lievens B, Thevelein JM. Phenotypic landscape of non-conventional yeast species for different stress tolerance traits desirable in bioethanol fermentation. Biotechnol Biofuels. 2017;10: 216 10.1186/s13068-017-0899-5 28924451 PMC5597992

[19] Wang ZX, Zhuge J, Fang H, Prior BA. Glycerol production by microbial fermentation: a review. Biotechnol Adv. 2001;19: 201–223. 14538083 10.1016/s0734-9750(01)00060-x

[20] Chan GF, Gan HM, Ling HL, Rashid NA. Genome sequence of *Pichia kudriavzevii* M12, a potential producer of bioethanol and phytase. Eukaryot Cell. 2012;11: 1300–1301. 10.1128/EC.00229-12 23027839 PMC3485917

[21] van Rijswijck IM, Derks MF, Abee T, de Ridder D, Smid EJ. Genome sequences of *Cyberlindnera fabianii* 65, *Pichia kudriavzevii* 129, and *Saccharomyces cerevisiae* 131 isolated from fermented masau fruits in Zimbabwe. Genome Announc. 2017;5. 10.1128/genomeA.00064-17 PMC5383881 28385833

[22] Park HJ, Ko HJ, Jeong H, Lee SH, Ko HJ, Bae JH, et al Draft genome sequence of a multistress-tolerant yeast, *Pichia kudriavzevii* NG7. Genome Announc. 2018;6. 10.1128/genomeA.01515-17 PMC5773747 29348362

[23] Cuomo CA, Shea T, Yang B, Rao R, Forche A. Whole genome sequence of the heterozygous clinical isolate *Candida krusei* 81-B-5. G3 (Bethesda). 2017;7: 2883–2889. 28696923 10.1534/g3.117.043547 PMC5592916

[24] Jacobsen MD, Gow NA, Maiden MC, Shaw DJ, Odds FC. Strain typing and determination of population structure of *Candida krusei* by multilocus sequence typing. J Clin Microbiol. 2007;45: 317–323. 10.1128/JCM.01549-06 17122025 PMC1829042

[25] Perez-Torrado R, Querol A. Opportunistic strains of *Saccharomyces cerevisiae*: A potential risk sold in food products. Front Microbiol. 2015;6: 1522 10.3389/fmicb.2015.01522 26779173 PMC4705302

[26] Lamping E, Zhu JY, Niimi M, Cannon RD. Role of ectopic gene conversion in the evolution of a *Candida krusei* pleiotropic drug resistance transporter family. Genetics. 2017;205: 1619–1639. 10.1534/genetics.116.194811 28159755 PMC5378117

[27] Lamping E, Ranchod A, Nakamura K, Tyndall JD, Niimi K, Holmes AR, et al Abc1p is a multidrug efflux transporter that tips the balance in favor of innate azole resistance in *Candida krusei*. Antimicrob Agents Chemother. 2009;53: 354–369. 10.1128/AAC.01095-08 19015352 PMC2630665

[28] Morio F, Jensen RH, Le Pape P, Arendrup MC. Molecular basis of antifungal drug resistance in yeasts. Int J Antimicrob Agents. 2017;50: 599–606. 10.1016/j.ijantimicag.2017.05.012 28669835

[29] Tavernier E, Desnos-Ollivier M, Honeyman F, Srour M, Fayard A, Cornillon J, et al Development of echinocandin resistance in *Candida krusei* isolates following exposure to micafungin and caspofungin in a BM transplant unit. Bone Marrow Transplant. 2015;50: 158–160. 10.1038/bmt.2014.230 25402414

[30] Jensen RH, Justesen US, Rewes A, Perlin DS, Arendrup MC. Echinocandin failure case due to a previously unreported *FKS1* mutation in *Candida krusei*. Antimicrob Agents Chemother. 2014;58: 3550–3552. 10.1128/AAC.02367-14 24687511 PMC4068455

[31] Shen XX, Zhou X, Kominek J, Kurtzman CP, Hittinger CT, Rokas A. Reconstructing the backbone of the Saccharomycotina yeast phylogeny using genome-scale data. G3 (Bethesda). 2016;6: 3927–3939. 27672114 10.1534/g3.116.034744 PMC5144963

[32] Krassowski T, Coughlan AY, Shen XX, Zhou X, Kominek J, Opulente DA, et al Evolutionary instability of CUG-Leu in the genetic code of budding yeasts. Nat Commun. 2018;9: 1887 10.1038/s41467-018-04374-7 29760453 PMC5951914

[33] Dujon B. Yeasts illustrate the molecular mechanisms of eukaryotic genome evolution. Trends Genet. 2006;22: 375–387. 10.1016/j.tig.2006.05.007 16730849

[34] Guitard J, Atanasova R, Brossas JY, Meyer I, Gits M, Marinach C, et al *Candida inconspicua* and *Candida norvegensis*: new insights into identification in relation to sexual reproduction and genome organization. J Clin Microbiol. 2015;53: 1655–1661. 10.1128/JCM.02913-14 25762773 PMC4400776

[35] Proux-Wéra E, Armisén D, Byrne KP, Wolfe KH. A pipeline for automated annotation of yeast genome sequences by a conserved-synteny approach. BMC Bioinformatics. 2012;13: 237 10.1186/1471-2105-13-237 22984983 PMC3507789

[36] Simao FA, Waterhouse RM, Ioannidis P, Kriventseva EV, Zdobnov EM. BUSCO: assessing genome assembly and annotation completeness with single-copy orthologs. Bioinformatics. 2015;31: 3210–3212. 10.1093/bioinformatics/btv351 26059717

[37] Delcher AL, Kasif S, Fleischmann RD, Peterson J, White O, Salzberg SL. Alignment of whole genomes. Nucleic Acids Res. 1999;27: 2369–2376. 10325427 10.1093/nar/27.11.2369 PMC148804

[38] Lowe TM, Eddy SR. tRNAscan-SE: a program for improved detection of transfer RNA genes in genomic sequence. Nucleic Acids Res. 1997;25: 955–964. 9023104 10.1093/nar/25.5.955 PMC146525

[39] Neuveglise C, Feldmann H, Bon E, Gaillardin C, Casaregola S. Genomic evolution of the long terminal repeat retrotransposons in hemiascomycetous yeasts. Genome Res. 2002;12: 930–943. 10.1101/gr.219202 12045146 PMC1383729

[40] Coughlan AY, Hanson SJ, Byrne KP, Wolfe KH. Centromeres of the yeast *Komagataella phaffii (Pichia pastoris)* have a simple inverted-repeat structure. Genome Biol Evol. 2016;8: 2482–2492. 10.1093/gbe/evw178 27497317 PMC5010909

[41] Chatterjee G, Sankaranarayanan SR, Guin K, Thattikota Y, Padmanabhan S, Siddharthan R, et al Repeat-associated fission yeast-like regional centromeres in the ascomycetous budding yeast *Candida tropicalis*. PLoS Genet. 2016;12: e1005839 10.1371/journal.pgen.1005839 26845548 PMC4741521

[42] Cohn M, McEachern MJ, Blackburn EH. Telomeric sequence diversity within the genus *Saccharomyces*. Curr Genet. 1998;33: 83–91. 9506895 10.1007/s002940050312

[43] Neuveglise C, Marck C, Gaillardin C. The intronome of budding yeasts. C R Biol. 2011;334: 662–670. 10.1016/j.crvi.2011.05.015 21819948

[44] Parenteau J, Durand M, Morin G, Gagnon J, Lucier JF, Wellinger RJ, et al Introns within ribosomal protein genes regulate the production and function of yeast ribosomes. Cell. 2011;147: 320–331. 10.1016/j.cell.2011.08.044 22000012

[45] Rajkowska K, Kunicka-Styczynska A, Peczek M. Hydrophobic properties of *Candida* spp. under the influence of selected essential oils. Acta Biochim Pol. 2015;62: 663–668. 10.18388/abp.2015_1096 26601324

[46] Li H, Durbin R. Fast and accurate short read alignment with Burrows-Wheeler transform. Bioinformatics. 2009;25: 1754–1760. 10.1093/bioinformatics/btp324 19451168 PMC2705234

[47] Carreté L, Ksiezopolska E, Pegueroles C, Gomez-Molero E, Saus E, Iraola-Guzman S, et al Patterns of genomic variation in the opportunistic pathogen *Candida glabrata* suggest the existence of mating and a secondary association with humans. Curr Biol. 2018;28: 15–27.e17. 10.1016/j.cub.2017.11.027 29249661 PMC5772174

[48] Hirakawa MP, Martinez DA, Sakthikumar S, Anderson MZ, Berlin A, Gujja S, et al Genetic and phenotypic intra-species variation in *Candida albicans*. Genome Res. 2015;25: 413–425. 10.1101/gr.174623.114 25504520 PMC4352881

[49] Peter J, De Chiara M, Friedrich A, Yue JX, Pflieger D, Bergstrom A, et al Genome evolution across 1,011 *Saccharomyces cerevisiae* isolates. Nature. 2018; 10.1038/s41586-018-0030-5 29643504 PMC6784862

[50] Skoneczna A, Kaniak A, Skoneczny M. Genetic instability in budding and fission yeast-sources and mechanisms. FEMS Microbiol Rev. 2015;39: 917–967. 10.1093/femsre/fuv028 26109598 PMC4608483

[51] Katz Ezov T, Boger-Nadjar E, Frenkel Z, Katsperovski I, Kemeny S, Nevo E, et al Molecular-genetic biodiversity in a natural population of the yeast *Saccharomyces cerevisiae* from "Evolution Canyon": microsatellite polymorphism, ploidy and controversial sexual status. Genetics. 2006;174: 1455–1468. 10.1534/genetics.106.062745 16980391 PMC1667085

[52] Zhu YO, Sherlock G, Petrov DA. Whole genome analysis of 132 clinical *Saccharomyces cerevisiae* strains reveals extensive ploidy variation. G3 (Bethesda). 2016;6: 2421–2434. 27317778 10.1534/g3.116.029397 PMC4978896

[53] Nguyen LT, Schmidt HA, von Haeseler A, Minh BQ. IQ-TREE: a fast and effective stochastic algorithm for estimating maximum-likelihood phylogenies. Mol Biol Evol. 2015;32: 268–274. 10.1093/molbev/msu300 25371430 PMC4271533

[54] Pritchard JK, Stephens M, Donnelly P. Inference of population structure using multilocus genotype data. Genetics. 2000;155: 945–959. 10835412 10.1093/genetics/155.2.945 PMC1461096

[55] Hautala T, Kakko S, Siitonen T, Saily M, Koistinen P, Koskela M. Clinical *Candida krusei* isolates remain susceptible during extensive exposure to antifungal drugs. Med Mycol. 2010;48: 79–84. 10.3109/13693780902725276 19194820

[56] Scarsini M, Tomasinsig L, Arzese A, D’Este F, Oro D, Skerlavaj B. Antifungal activity of cathelicidin peptides against planktonic and biofilm cultures of *Candida* species isolated from vaginal infections. Peptides. 2015;71: 211–221. 10.1016/j.peptides.2015.07.023 26238597

[57] He X, Zhao M, Chen J, Wu R, Zhang J, Cui R, et al Overexpression of both *ERG11* and *ABC2* genes might be responsible for itraconazole resistance in clinical isolates of *Candida krusei*. PLoS One. 2015;10: e0136185 10.1371/journal.pone.0136185 26308936 PMC4550294

[58] Rodriguez-Tudela JL, Arendrup MC, Barchiesi F, Bille J, Chryssanthou E, Cuenca-Estrella M, et al EUCAST definitive document EDef 7.1: method for the determination of broth dilution MICs of antifungal agents for fermentative yeasts. Clin Microbiol Infect. 2008;14: 398–405. 10.1111/j.1469-0691.2007.01935.x 18190574

[59] Pfaller MA, Castanheira M, Messer SA, Rhomberg PR, Jones RN. Comparison of EUCAST and CLSI broth microdilution methods for the susceptibility testing of 10 systemically active antifungal agents when tested against *Candida* spp. Diagn Microbiol Infect Dis. 2014;79: 198–204. 10.1016/j.diagmicrobio.2014.03.004 24736096

[60] Pappas PG, Kauffman CA, Andes DR, Clancy CJ, Marr KA, Ostrosky-Zeichner L, et al Executive Summary: Clinical practice guideline for the management of Candidiasis: 2016 update by the Infectious Diseases Society of America. Clin Infect Dis. 2016;62: 409–417. 10.1093/cid/civ1194 26810419

[61] Gao X, Zhuge B, Fang H, Zhuge J. The construction of a new integrative vector with a new selective marker of copper resistance for glycerol producer *Candida glycerinogenes*. Curr Microbiol. 2012;64: 357–364. 10.1007/s00284-011-0075-2 22237983

[62] Ji H, Zhuge B, Zong H, Lu X, Fang H, Zhuge J. Role of *CgHOG1* in stress responses and glycerol overproduction of *Candida glycerinogenes*. Curr Microbiol. 2016;73: 827–833. 10.1007/s00284-016-1132-7 27620385

[63] Taylor JW. One Fungus = One Name: DNA and fungal nomenclature twenty years after PCR. IMA Fungus. 2011;2: 113–120. 10.5598/imafungus.2011.02.02.01 22679595 PMC3359808

[64] Morales L, Noel B, Porcel B, Marcet-Houben M, Hullo MF, Sacerdot C, et al Complete DNA sequence of *Kuraishia capsulata* illustrates novel genomic features among budding yeasts (Saccharomycotina). Genome Biol Evol. 2013;5: 2524–2539. 10.1093/gbe/evt201 24317973 PMC3879985

[65] Allshire RC. Centromere and kinetochore structure and function In: Egel R, editor. The molecular biology of *Schizosaccharomyces pombe*. Berlin: Springer; 2004 p. 149–169.

[66] Hanson SJ, Wolfe KH. An evolutionary perspective on yeast mating-type switching. Genetics. 2017;206: 9–32. 10.1534/genetics.117.202036 28476860 PMC5419495

[67] Coelho AC, Garcia Diez J. Biological risks and laboratory-acquired infections: A reality that cannot be ignored in health biotechnology. Front Bioeng Biotechnol. 2015;3: 56 10.3389/fbioe.2015.00056 25973418 PMC4412124

[68] Berg G, Martinez JL. Friends or foes: can we make a distinction between beneficial and harmful strains of the *Stenotrophomonas maltophilia* complex? Front Microbiol. 2015;6: 241 10.3389/fmicb.2015.00241 25873912 PMC4379930

[69] Walker BJ, Abeel T, Shea T, Priest M, Abouelliel A, Sakthikumar S, et al Pilon: an integrated tool for comprehensive microbial variant detection and genome assembly improvement. PLoS One. 2014;9: e112963 10.1371/journal.pone.0112963 25409509 PMC4237348

[70] Bankevich A, Nurk S, Antipov D, Gurevich AA, Dvorkin M, Kulikov AS, et al SPAdes: a new genome assembly algorithm and its applications to single-cell sequencing. J Comput Biol. 2012;19: 455–477. 10.1089/cmb.2012.0021 22506599 PMC3342519

[71] Popolo L, Vanoni M, Alberghina L. Control of the yeast cell cycle by protein synthesis. Exp Cell Res. 1982;142: 69–78. 6754401 10.1016/0014-4827(82)90410-4

[72] Li H, Handsaker B, Wysoker A, Fennell T, Ruan J, Homer N, et al The Sequence Alignment/Map format and SAMtools. Bioinformatics. 2009;25: 2078–2079. 10.1093/bioinformatics/btp352 19505943 PMC2723002

[73] Van der Auwera GA, Carneiro MO, Hartl C, Poplin R, Del Angel G, Levy-Moonshine A, et al From FastQ data to high confidence variant calls: the Genome Analysis Toolkit best practices pipeline. Curr Protoc Bioinformatics. 2013;43: 11.10.11–33. 10.1002/0471250953.bi1110s43 25431634 PMC4243306

[74] Lischer HE, Excoffier L, Heckel G. Ignoring heterozygous sites biases phylogenomic estimates of divergence times: implications for the evolutionary history of microtus voles. Mol Biol Evol. 2014;31: 817–831. 10.1093/molbev/mst271 24371090

[75] Bailey TL, Boden M, Buske FA, Frith M, Grant CE, Clementi L, et al MEME SUITE: tools for motif discovery and searching. Nucleic Acids Res. 2009;37: W202–208. 10.1093/nar/gkp335 19458158 PMC2703892

[76] Riley R, Haridas S, Wolfe KH, Lopes MR, Hittinger CT, Goker M, et al Comparative genomics of biotechnologically important yeasts. Proc Natl Acad Sci USA. 2016;113: 9882–9887. 10.1073/pnas.1603941113 27535936 PMC5024638

[77] Katoh K, Standley DM. MAFFT multiple sequence alignment software version 7: improvements in performance and usability. Mol Biol Evol. 2013;30: 772–780. 10.1093/molbev/mst010 23329690 PMC3603318

[78] Guindon S, Dufayard JF, Lefort V, Anisimova M, Hordijk W, Gascuel O. New algorithms and methods to estimate maximum-likelihood phylogenies: assessing the performance of PhyML 3.0. Syst Biol. 2010;59: 307–321. 10.1093/sysbio/syq010 20525638

