# Supplementary figures and images for: Population genomics shows no distinction between pathogenic Candida krusei and environmental Pichia kudriavzevii: One species, four names
Source: PLoS Pathog. 2018 Jul 19;14(7):e1007138. doi: 10.1371/journal.ppat.1007138 (PMC6053246; doi:10.1371/journal.ppat.1007138)

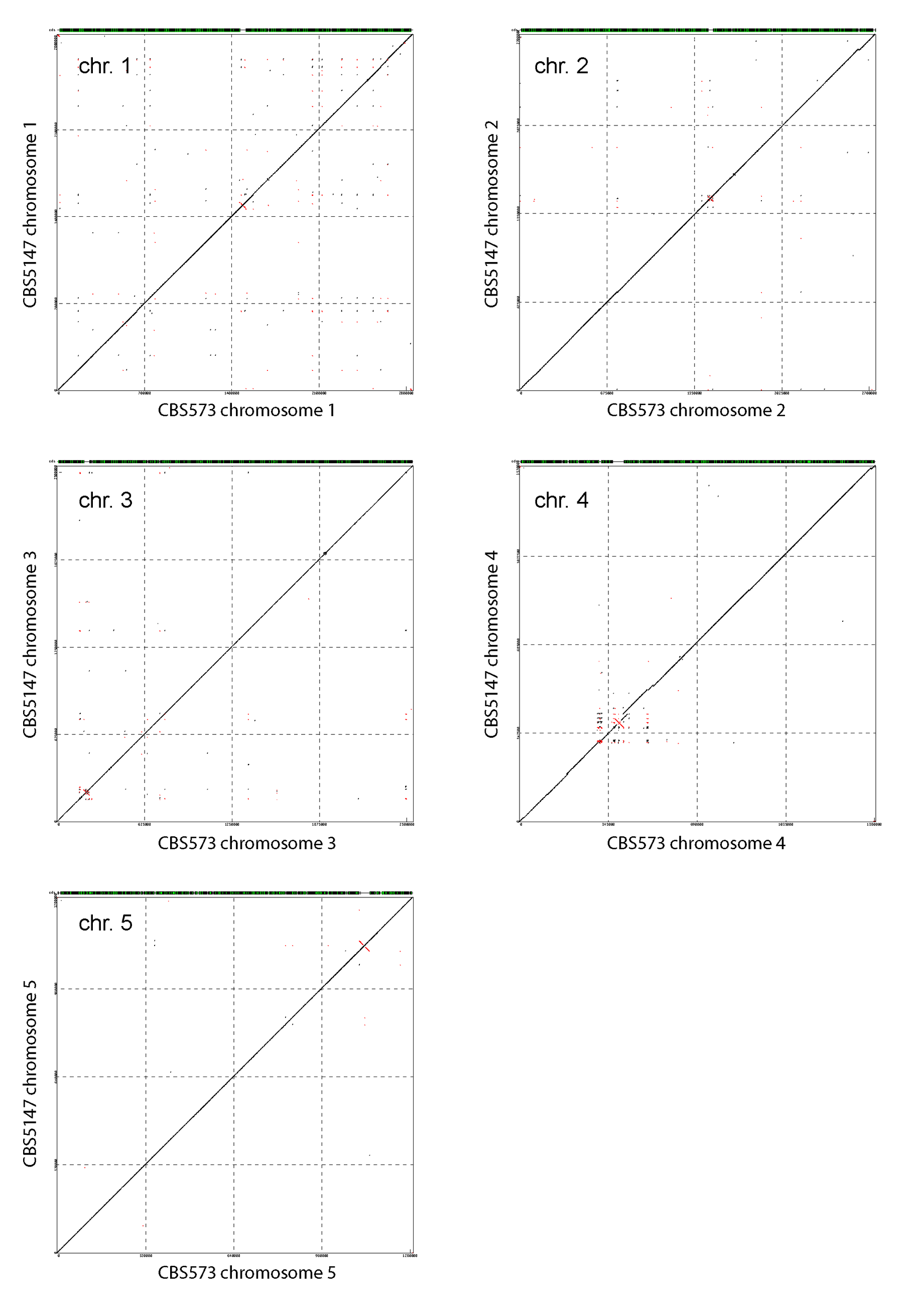

Supplement: S1 Fig — Dot matrix plots compare PacBio assemblies of CBS5147 chromosomes (Y-axis) versus CBS573 chromosomes (X-axis). Black diagonals indicate matches in the same orientation, and red diagonals indicate matches in opposite orientations. Plots were constructed using DNAMAN (www.lynnon.com), with a criterion of 50 matches per 50-bp window. Bars at the top of the plots show the locations of annotated protein-coding genes in the CBS573 genome, with an absence of genes at the centromeres. (TIF) [file ppat.1007138.s001.tif]

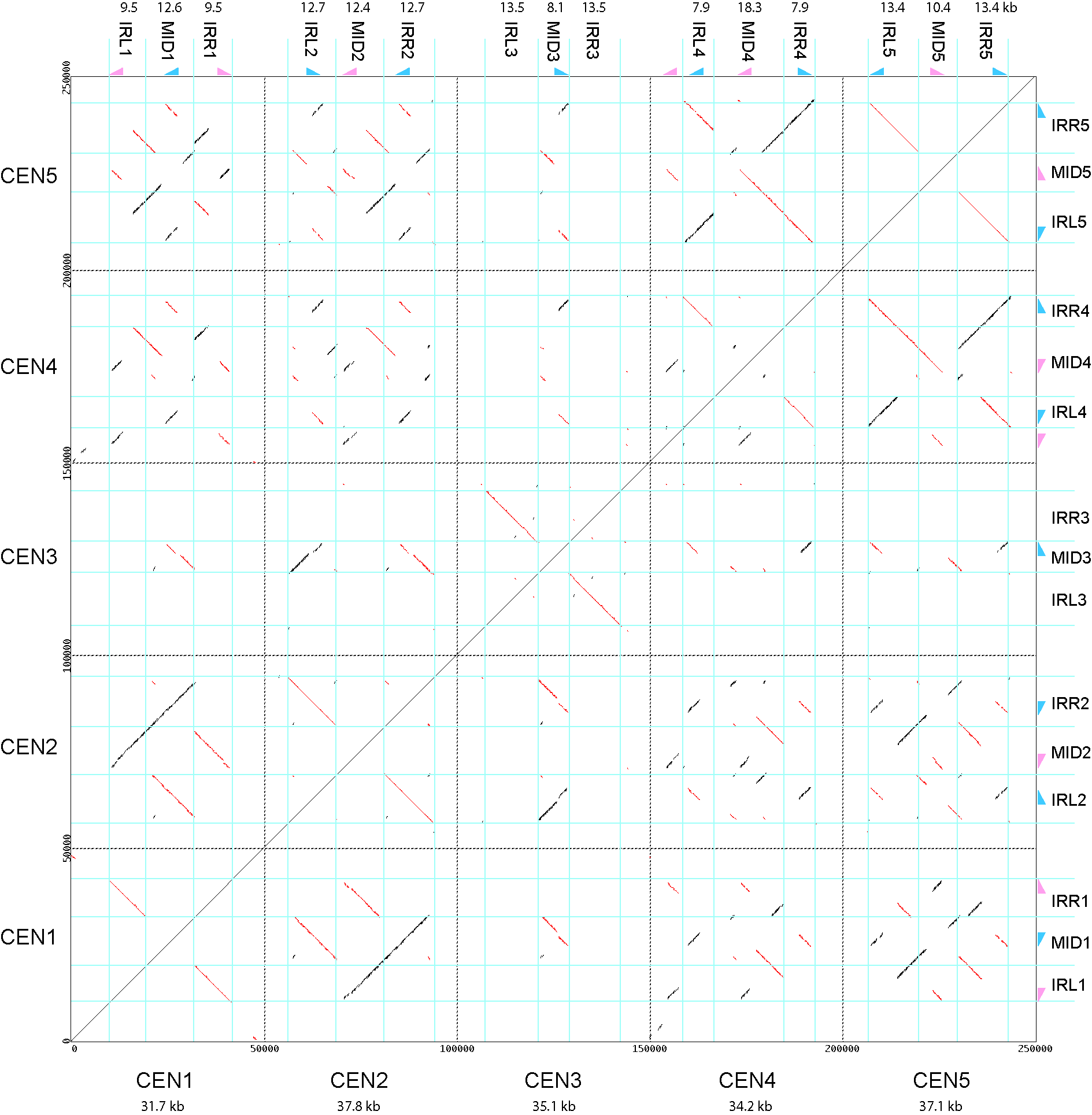

Supplement: S2 Fig — 50-kb regions around the centromeres of the 5 chromosomes of CBS573 were concatenated and compared in a dot matrix plot. Black diagonals indicate matches in the same orientation, and red diagonals indicate matches in opposite orientations. Dashed lines mark the ends of the 50-kb section from each chromosome. The cyan grid marks the ends of the three sections of each centromere (IRL, left part of the IR; MID, middle region; IRR, right part of the IR). Locations of PkudTy3A pseudogenes (pink triangles) and PkudTy3B pseudogenes (blue triangles) are shown. The plot was constructed using DNAMAN (www.lynnon.com), with a criterion of 50 matches per 50-bp window. (TIF) [file ppat.1007138.s002.tif]

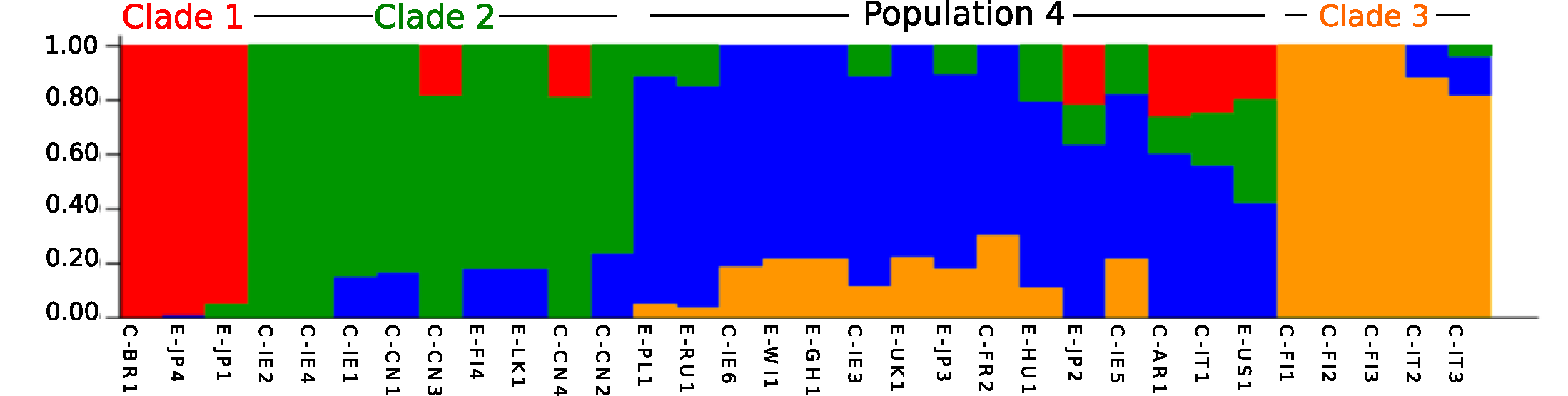

Supplement: S3 Fig — The diagram was built from a filtered dataset of 150,306 SNP sites using STRUCTURE [54] with k = 4. Each column represents a strain, and the colors represent the proportion of sites belonging to each of the 4 inferred populations. Populations 1–3 form monophyletic clades in the tree in Fig 6, but population 4 does not. (TIF) [file ppat.1007138.s003.tif]

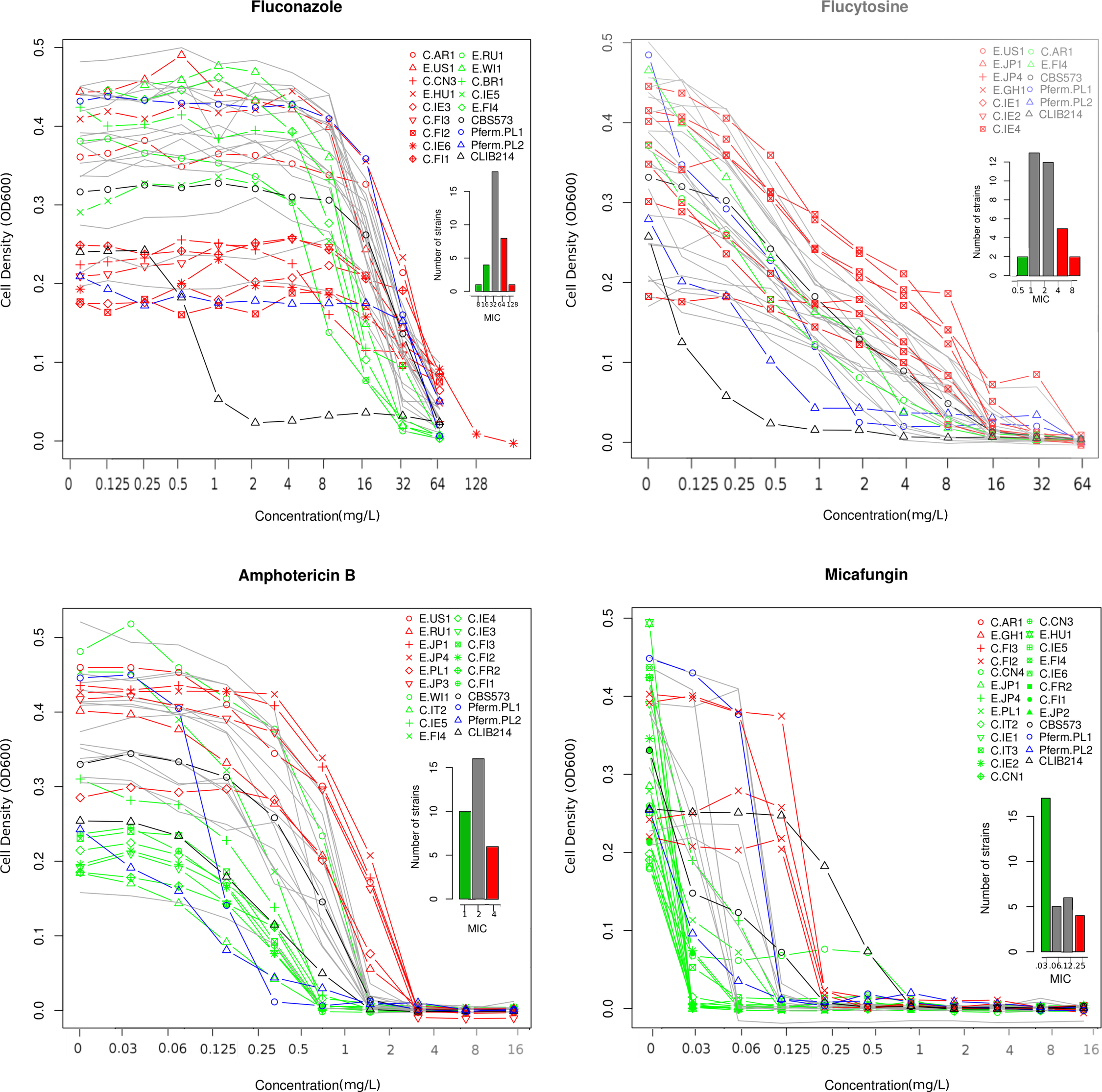

Supplement: S4 Fig — Growth of strains after 24 hours (OD600) is plotted versus drug concentration for four drugs: (A) fluconazole, (B) flucytosine, (C) amphotericin B, and (D) micafungin. Histograms (insets) show the distribution of Minimum Inhibitory Concentration (MIC) values for all strains. For P. kudriavzevii, only strains designated as relatively resistant (red) or relatively sensitive (green) are identified in the keys; other strains are plotted as gray lines. Two strains of P. fermentans (blue) and the EUCAST control strains of C. krusei (CBS573T; black circles) and C. parapsilosis (CLIB214; black triangles) are also plotted. MIC is defined as the concentration required to inhibit 50% of growth in fluconazole, flucytosine and micafungin, and 90% in amphotericin B [58]. Each data point is the average of three biological replicates. (TIF) [file ppat.1007138.s004.tif]
