# Supplementary material for: Population genomics shows no distinction between pathogenic Candida krusei and environmental Pichia kudriavzevii: One species, four names
Source: PLoS Pathog. 2018 Jul 19;14(7):e1007138. doi: 10.1371/journal.ppat.1007138 (PMC6053246; doi:10.1371/journal.ppat.1007138)
Supplement: S1 Table — Annotated protein datasets for C. krusei strain 81-B-5 [23], I. orientalis strain SD108 [10], and P. kudriavzevii strain 129 [21] were downloaded from the NCBI database. BUSCO version 3.0.2 (busco.ezlab.org) [36] was used to compare these annotations and our CBS573 annotation to two reference datasets of single-copy genes that are universally conserved in the Ascomycota lineage, or in the Saccharomycetales lineage. The BUSCO reports show the percentages of proteins in the reference datasets whose orthologs are complete (C), fragmented (F), or missing (M) in each annotation. Complete proteins are subdivided into those that are single-copy (S) or duplicated (D) in the annotations. (DOCX) [file ppat.1007138.s005.docx]

**Table S1.** Evaluation of genome annotation quality using BUSCO.

| Protein dataset | NCBI BioProject | Number of proteins | BUSCO Report  (Ascomycota lineage dataset) | BUSCO Report  (Saccharomycetales lineage dataset) |
| --- | --- | --- | --- | --- |
| CBS573 (this study) | PRJNA434433 | 5140 | C:91.7%[S:91.2%,D:0.5%],F:4.3%,M:4.0%,n:1315 | C:80.0%[S:79.4%,D:0.6%],F:9.4%,M:10.6%,n:1711 |
| 81-B-5 (Cuomo *et al.*) | PRJNA381554 | 4949 | C:86.3%[S:85.6%,D:0.7%],F:7.1%,M:6.6%,n:1315 | C:74.7%[S:74.1%,D:0.6%],F:11.9%,M:13.4%,n:1711 |
| SD108 (Xiao *et al.*) | PRJNA257499 | 7107 | C:82.4%[S:79.3%,D:3.1%],F:9.8%,M:7.8%,n:1315 | C:71.0%[S:67.7%,D:3.3%],F:12.8%,M:16.2%,n:1711 |
| 129 (van Rijswijck *et al.*) | PRJNA381597 | 5385 | C:79.6%[S:76.9%,D:2.7%],F:11.3%,M:9.1%,n:1315 | C:66.6%[S:64.0%,D:2.6%],F:14.2%,M:19.2%,n:1711 |
