# Supplementary material for: Population genomics shows no distinction between pathogenic Candida krusei and environmental Pichia kudriavzevii: One species, four names
Source: PLoS Pathog. 2018 Jul 19;14(7):e1007138. doi: 10.1371/journal.ppat.1007138 (PMC6053246; doi:10.1371/journal.ppat.1007138)
Supplement: S2 Table — (DOCX) [file ppat.1007138.s006.docx]

**Table S2.** SNP densities and extent of loss-of-heterozygosity (LOH) regions in sequenced strains.

| **Strain** | **Number of SNPs*** | **SNP density (SNPs/kb)** | **Heterozygosities** | **% Heterozygous** | **Homozygosities** | **% Homozygous** | **LOH Regions†** |
| --- | --- | --- | --- | --- | --- | --- | --- |
| C-AR1 | 44751 | 4.14 | 27990 | 62.5 | 16761 | 37.5 | 57 |
| C-BR1 | 51072 | 4.73 | 40255 | 78.8 | 10817 | 21.2 | 5 |
| C-CN1 | 26790 | 2.48 | 14952 | 55.8 | 11838 | 44.2 | 51 |
| C-CN2 | 26544 | 2.46 | 14890 | 56.1 | 11654 | 43.9 | 59 |
| C-CN3 | 35652 | 3.30 | 26049 | 73.1 | 9603 | 26.9 | 25 |
| C-CN4 | 36627 | 3.39 | 27980 | 76.4 | 8647 | 23.6 | 11 |
| C-FI1 | 54701 | 5.06 | 44500 | 81.4 | 10201 | 18.6 | 2 |
| C-FI2 | 54820 | 5.08 | 44585 | 81.3 | 10235 | 18.7 | 2 |
| C-FI3 | 54751 | 5.07 | 44539 | 81.3 | 10212 | 18.7 | 2 |
| C-FR2 | 52803 | 4.89 | 41652 | 78.9 | 11151 | 21.1 | 9 |
| C-IE1 | 26117 | 2.42 | 13853 | 53.0 | 12264 | 47.0 | 56 |
| C-IE2 | 25590 | 2.37 | 12578 | 49.2 | 13012 | 50.8 | 75 |
| C-IE3 | 47156 | 4.37 | 30624 | 64.9 | 16532 | 35.1 | 40 |
| C-IE4 | 24640 | 2.28 | 11017 | 44.7 | 13623 | 55.3 | 94 |
| C-IE5 | 47303 | 4.38 | 36638 | 77.5 | 10665 | 22.5 | 4 |
| C-IE6 | 52412 | 4.85 | 40899 | 78.0 | 11513 | 22.0 | 13 |
| C-IT1 | 43329 | 4.01 | 27829 | 64.2 | 15500 | 35.8 | 62 |
| C-IT2 | 53069 | 4.91 | 41317 | 77.9 | 11752 | 22.1 | 5 |
| C-IT3 | 50830 | 4.71 | 38268 | 75.3 | 12562 | 24.7 | 28 |
| C-LK1 ** | 5196 | 0.48 | 4813 | 92.6 | 383 | 7.4 | 118 |
| E-FI4 | 25872 | 2.40 | 13588 | 52.5 | 12284 | 47.5 | 61 |
| E-GH1 | 49303 | 4.57 | 33811 | 68.6 | 15492 | 31.4 | 40 |
| E-HU1 | 42980 | 3.98 | 29303 | 68.2 | 13677 | 31.8 | 40 |
| E-JP1 | 48904 | 4.53 | 38089 | 77.9 | 10815 | 22.1 | 23 |
| E-JP2 | 44075 | 4.08 | 22654 | 51.4 | 21421 | 48.6 | 95 |
| E-JP3 | 45344 | 4.20 | 30441 | 67.1 | 14903 | 32.9 | 59 |
| E-JP4 | 50945 | 4.72 | 39497 | 77.5 | 11448 | 22.5 | 12 |
| E-PL1 | 51751 | 4.79 | 41663 | 80.5 | 10088 | 19.5 | 5 |
| E-RU1 | 41347 | 3.83 | 27413 | 66.3 | 13934 | 33.7 | 4 |
| E-UK1 | 54134 | 5.01 | 44123 | 81.5 | 10011 | 18.5 | 0 |
| E-US1 | 41667 | 3.86 | 29330 | 70.4 | 12337 | 29.6 | 30 |
| E-WI1 | 53579 | 4.96 | 43644 | 81.5 | 9935 | 18.5 | 0 |

* Number of SNPs after filtering to remove SNPs with frequency <0.15.

† Number of 50-kb windows scored as showing loss of heterozygosity, i.e. with less than 30 heterozygous sites in the 50 kb.

** Reference genome strain (CBS573).
