# Supplementary material for: Population genomics shows no distinction between pathogenic Candida krusei and environmental Pichia kudriavzevii: One species, four names
Source: PLoS Pathog. 2018 Jul 19;14(7):e1007138. doi: 10.1371/journal.ppat.1007138 (PMC6053246; doi:10.1371/journal.ppat.1007138)
Supplement: S1 File — For each strain, the plots show: (Top left) Allele frequency of non-reference alleles at polymorphic sites along the genome, as in Fig 5A. (Top right) Sequencing coverage along each chromosome. The red points are segmental means. (Lower 6 panels) Histograms of non-reference allele frequencies in the whole genome, and separately for each chromosome. (PDF) [file ppat.1007138.s007.pdf]

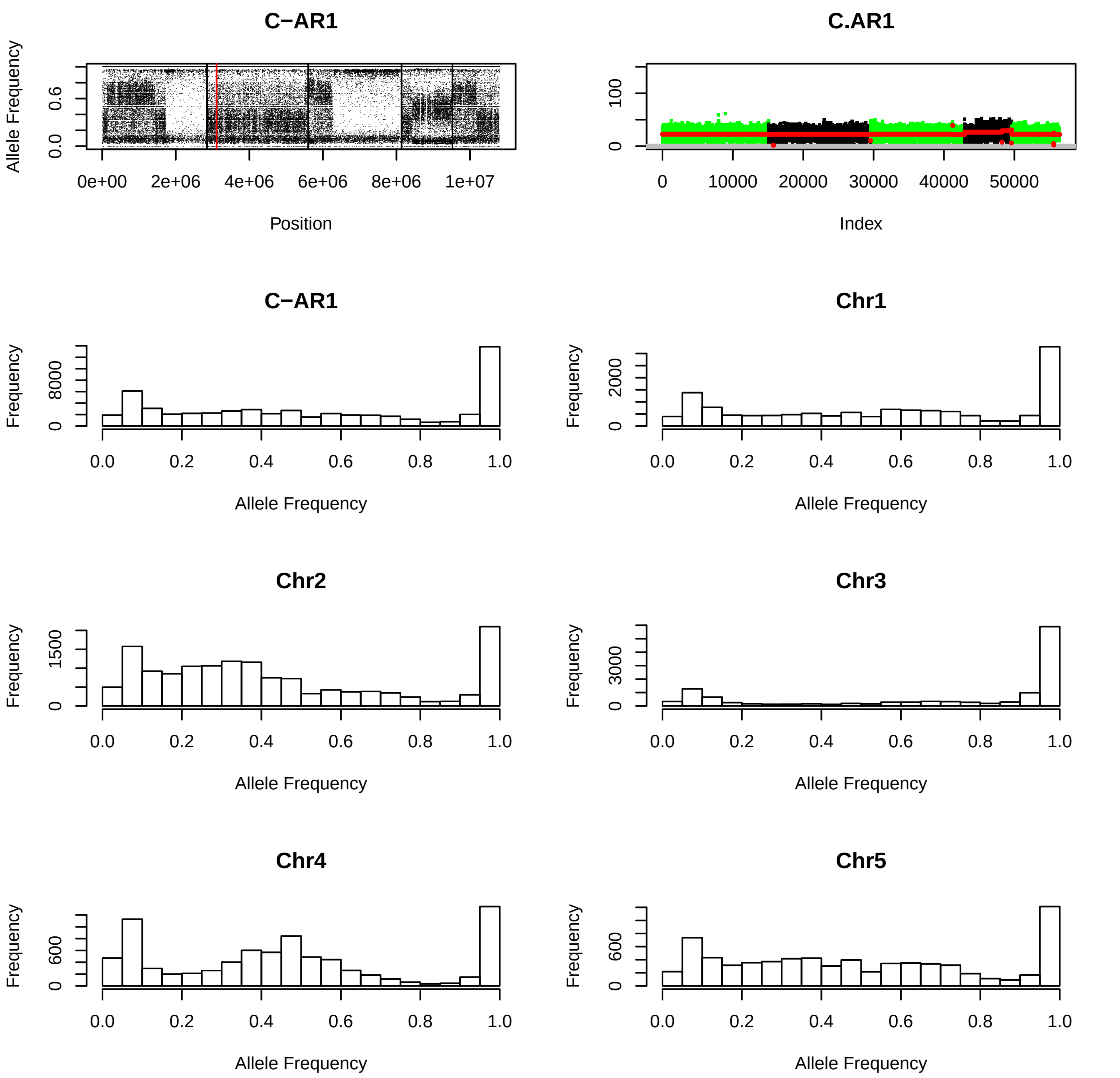
C−AR1
Allele Frequency
Position
0e+00
2e+06
4e+06
6e+06
8e+06
1e+07
0.0
0.6
C.AR1
Index
0
10000
20000
30000
40000
50000
100
C−AR1
Frequency
Allele Frequency
0
8000
Chr1
2000
Chr2
1500
Chr3
3000
Chr4
600
Chr5
600
0.0
0.2
0.4
0.6
0.8
1.0

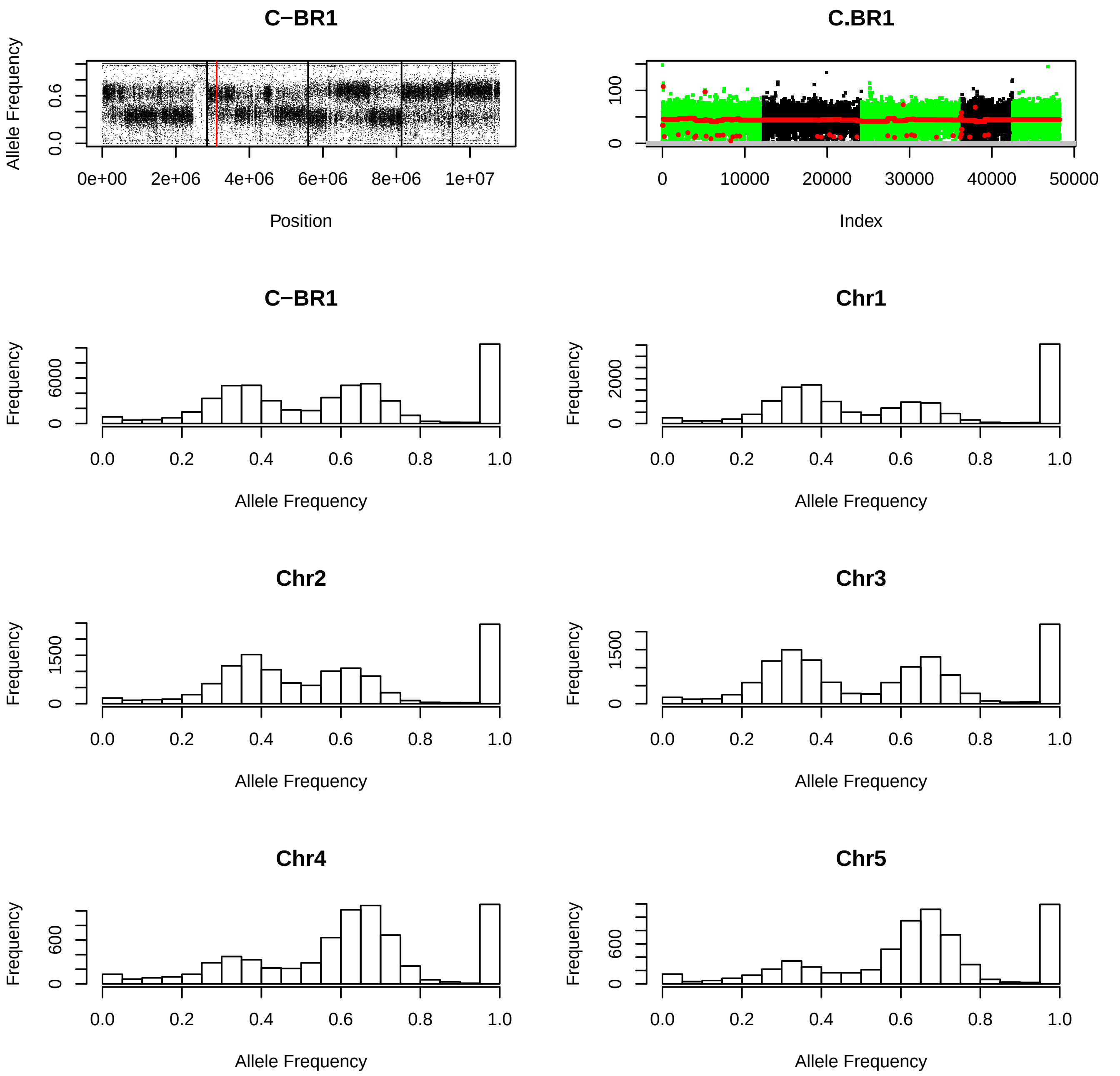
C−BR1
Allele Frequency
Position
C.BR1
Index
C−BR1
Chr1
Chr2
Chr3
Chr4
Chr5
Frequency
Allele Frequency

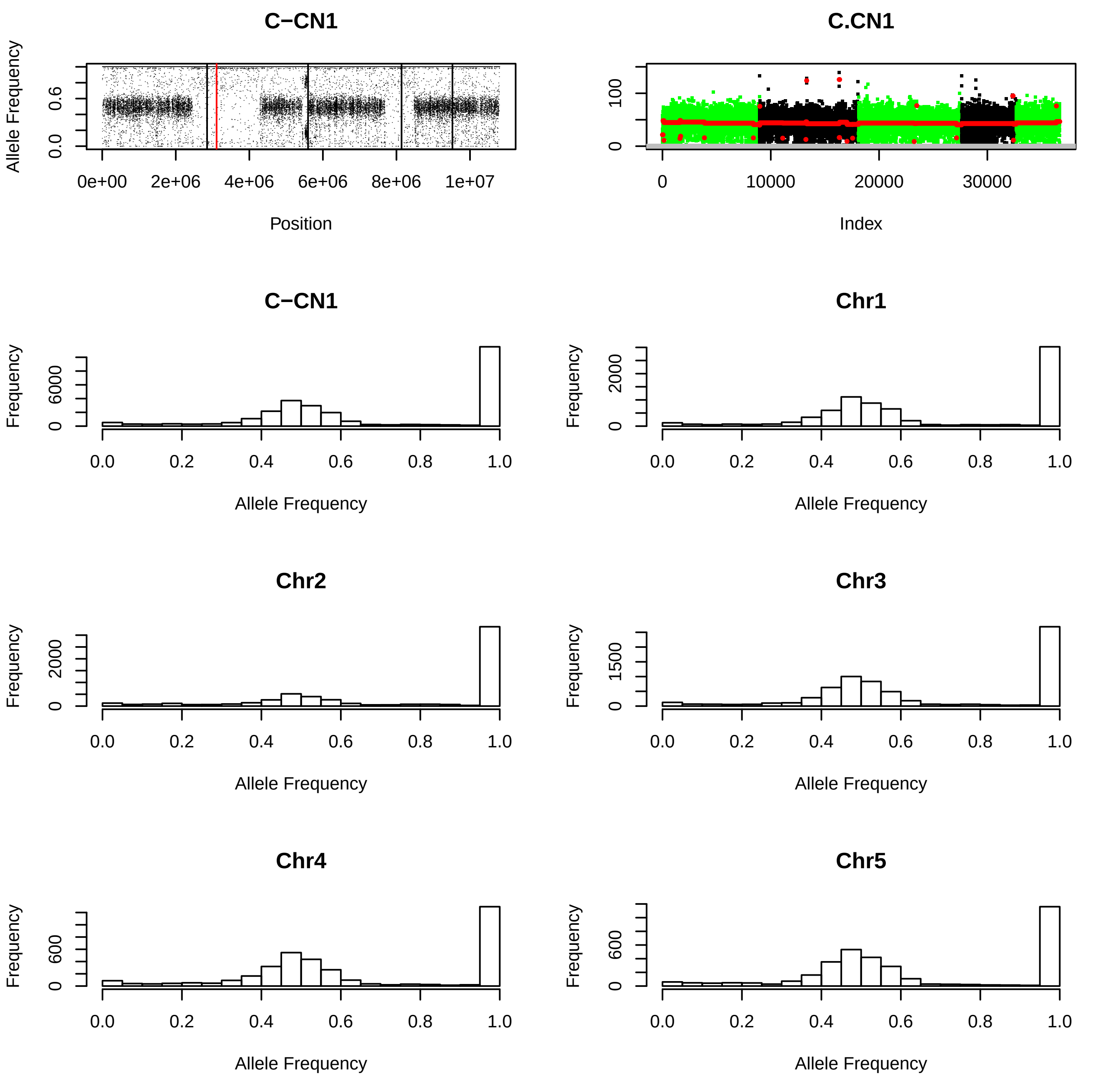
C−CN1
Allele Frequency
0.0
0.6
0e+00
2e+06
4e+06
6e+06
8e+06
1e+07
Position
C.CN1
0
100
0
10000
20000
30000
Index
C−CN1
Frequency
0
6000
0.0
0.2
0.4
0.6
0.8
1.0
Allele Frequency
Chr1
Frequency
0
2000
0.0
0.2
0.4
0.6
0.8
1.0
Allele Frequency
Chr2
Frequency
0
2000
0.0
0.2
0.4
0.6
0.8
1.0
Allele Frequency
Chr3
Frequency
0
1500
0.0
0.2
0.4
0.6
0.8
1.0
Allele Frequency
Chr4
Frequency
0
600
0.0
0.2
0.4
0.6
0.8
1.0
Allele Frequency
Chr5
Frequency
0
600
0.0
0.2
0.4
0.6
0.8
1.0
Allele Frequency

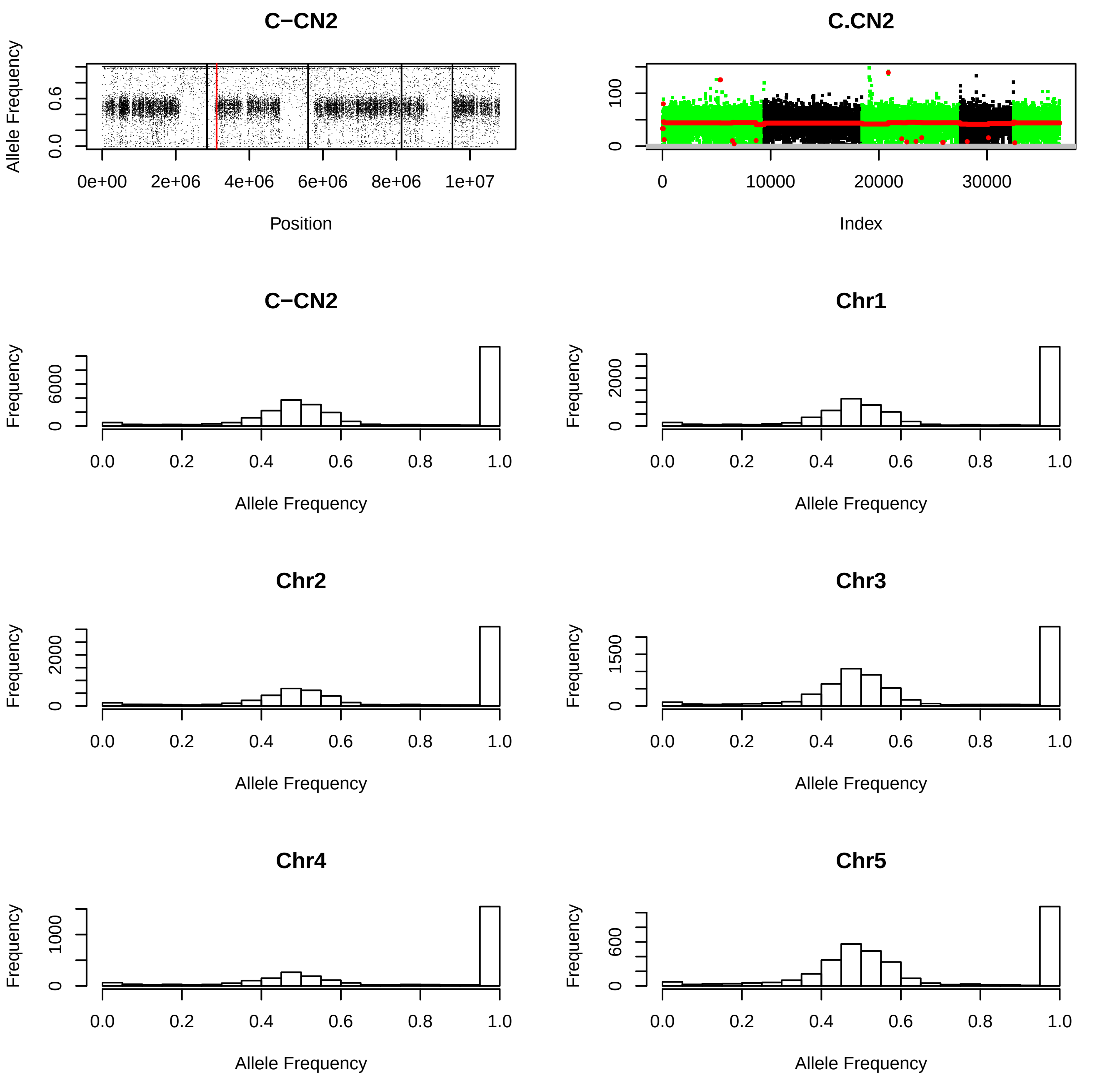
C−CN2
Allele Frequency
Position
C.CN2
Index
C−CN2
Chr1
Chr2
Chr3
Chr4
Chr5
Frequency
Allele Frequency

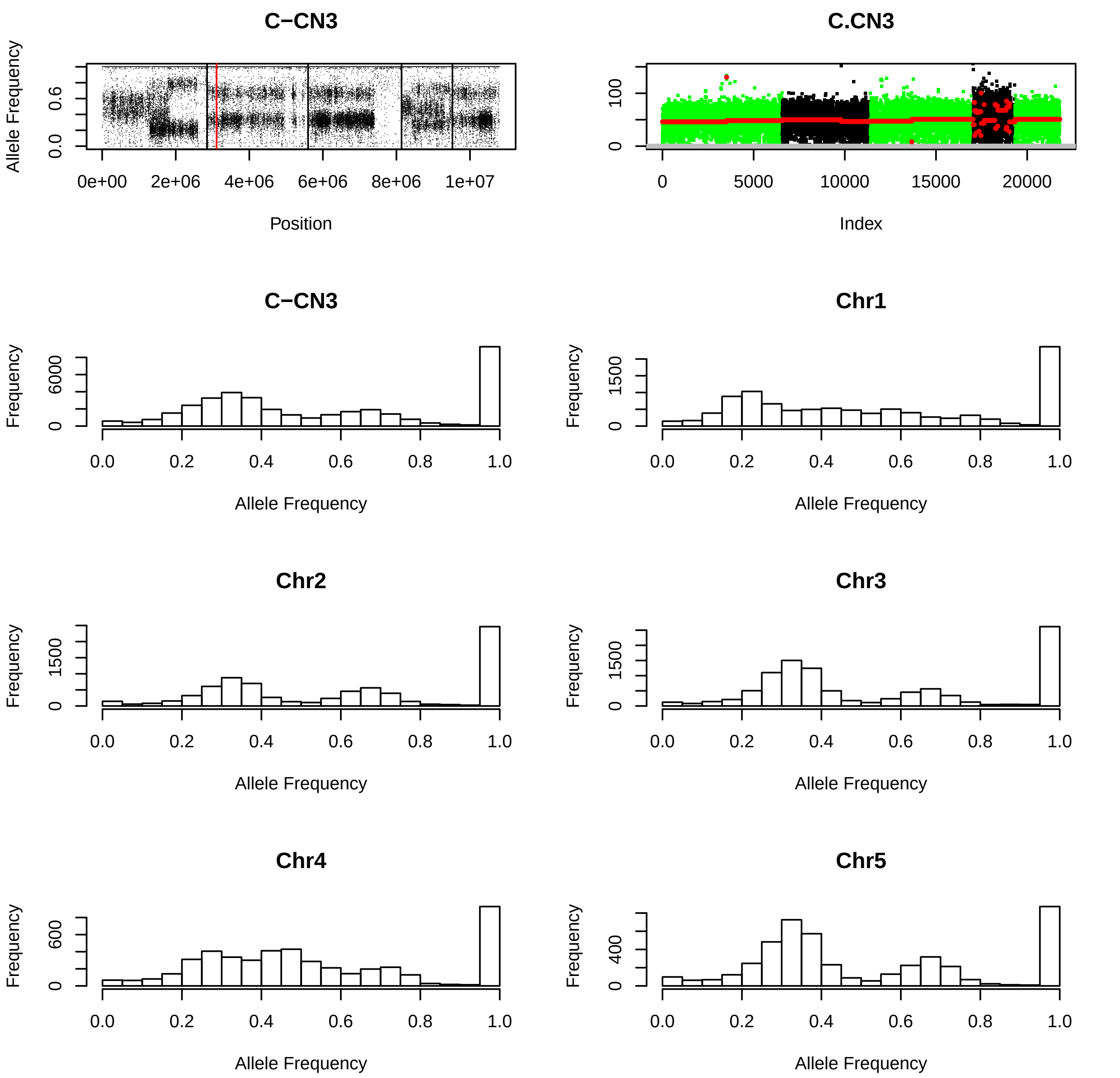
C−CN3
Allele Frequency
0.0
0.6
0e+00
2e+06
4e+06
6e+06
8e+06
1e+07
Position
C.CN3
0
100
0
5000
10000
15000
20000
Index
C−CN3
Frequency
0
6000
0.0
0.2
0.4
0.6
0.8
1.0
Allele Frequency
Chr1
Frequency
0
1500
Allele Frequency
Chr2
Frequency
0
1500
Allele Frequency
Chr3
Frequency
0
1500
Allele Frequency
Chr4
Frequency
0
600
Allele Frequency
Chr5
Frequency
0
400
Allele Frequency

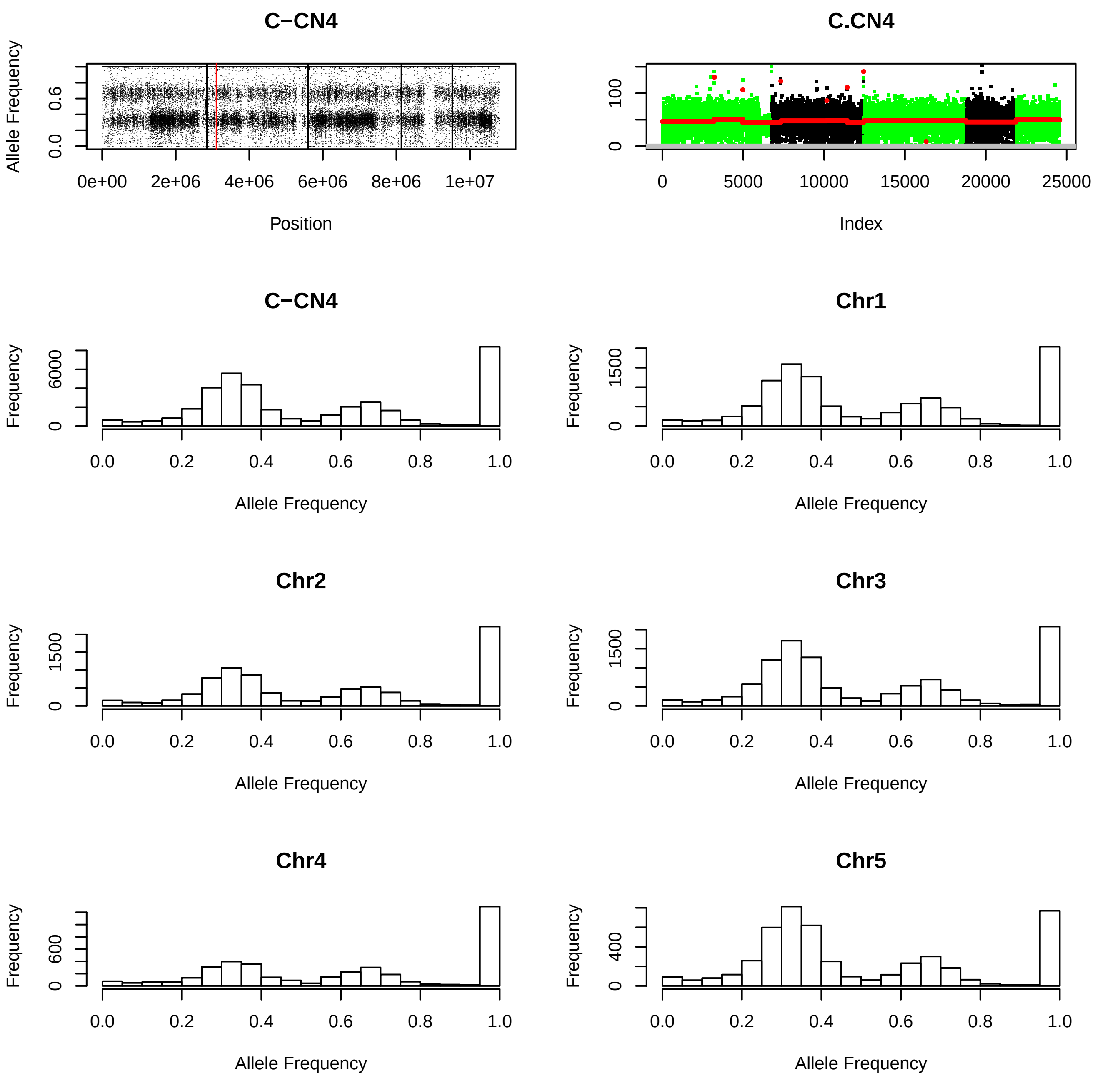
C−CN4
Allele Frequency
0.0
0.6
0e+00
2e+06
4e+06
6e+06
8e+06
1e+07
Position
C.CN4
0
100
0
5000
10000
15000
20000
25000
Index
C−CN4
Frequency
0
6000
0.0
0.2
0.4
0.6
0.8
1.0
Allele Frequency
Chr1
Frequency
0
1500
0.0
0.2
0.4
0.6
0.8
1.0
Allele Frequency
Chr2
Frequency
0
1500
0.0
0.2
0.4
0.6
0.8
1.0
Allele Frequency
Chr3
Frequency
0
1500
0.0
0.2
0.4
0.6
0.8
1.0
Allele Frequency
Chr4
Frequency
0
600
0.0
0.2
0.4
0.6
0.8
1.0
Allele Frequency
Chr5
Frequency
0
400
0.0
0.2
0.4
0.6
0.8
1.0
Allele Frequency

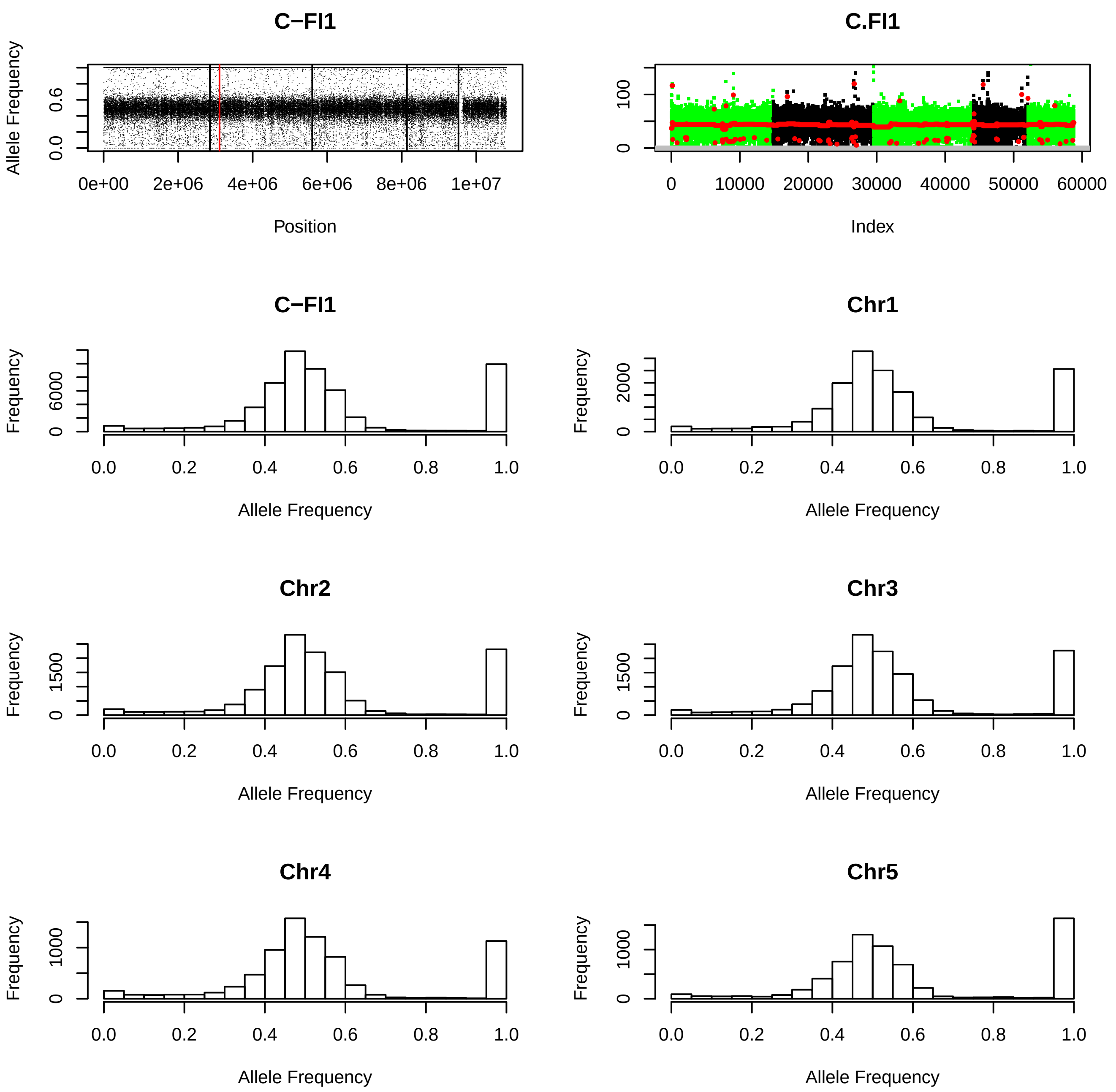
C−FI1
Allele Frequency
0.0
0.6
0e+00
2e+06
4e+06
6e+06
8e+06
1e+07
Position
C.FI1
0
100
0
10000
20000
30000
40000
50000
60000
Index
C−FI1
Frequency
0
6000
0.0
0.2
0.4
0.6
0.8
1.0
Allele Frequency
Chr1
Frequency
0
2000
0.0
0.2
0.4
0.6
0.8
1.0
Allele Frequency
Chr2
Frequency
0
1500
0.0
0.2
0.4
0.6
0.8
1.0
Allele Frequency
Chr3
Frequency
0
1500
0.0
0.2
0.4
0.6
0.8
1.0
Allele Frequency
Chr4
Frequency
0
1000
0.0
0.2
0.4
0.6
0.8
1.0
Allele Frequency
Chr5
Frequency
0
1000
0.0
0.2
0.4
0.6
0.8
1.0
Allele Frequency

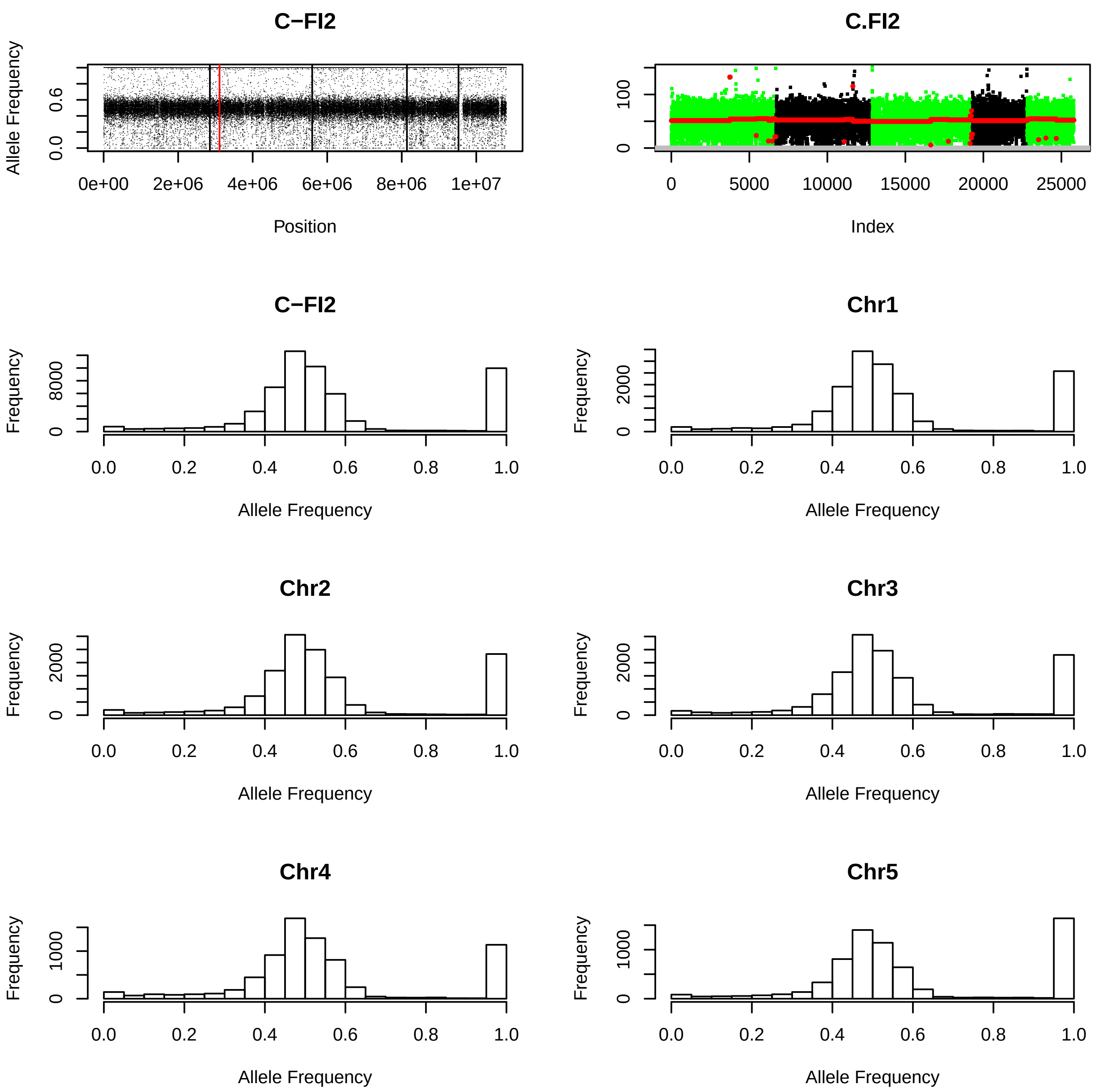

C−FI2
Allele Frequency
0.0
0.6
0e+00
2e+06
4e+06
6e+06
8e+06
1e+07
Position
C.FI2
0
100
0
5000
10000
15000
20000
25000
Index
C−FI2
Frequency
0
8000
0.0
0.2
0.4
0.6
0.8
1.0
Allele Frequency
Chr1
Frequency
0
2000
Allele Frequency
Chr2
Frequency
0
2000
Allele Frequency
Chr3
Frequency
0
2000
Allele Frequency
Chr4
Frequency
0
1000
Allele Frequency
Chr5
Frequency
0
1000
Allele Frequency

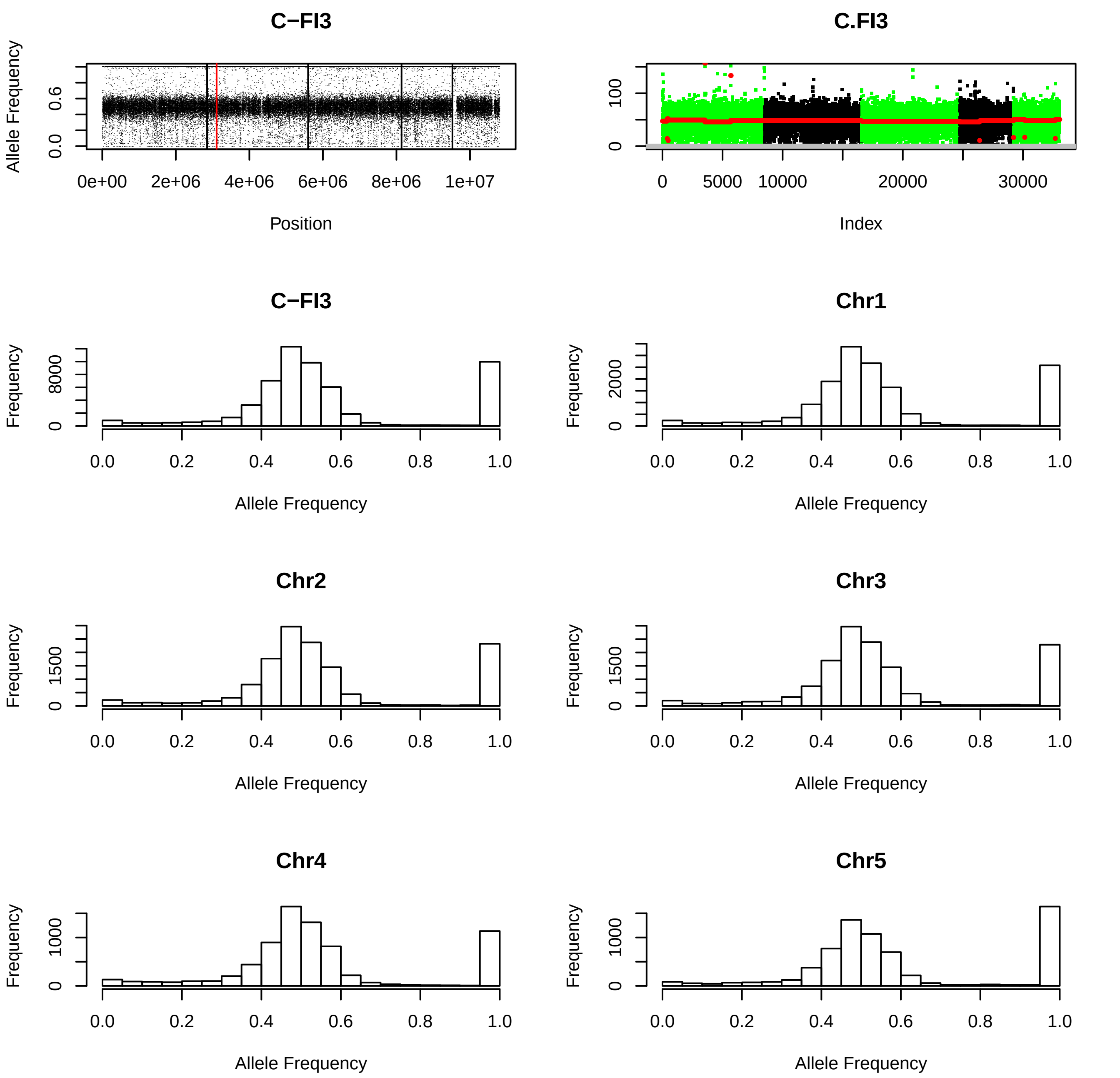

C−FI3
Allele Frequency
0.0
0.6
0e+00
2e+06
4e+06
6e+06
8e+06
1e+07
Position
C.FI3
0
100
0
5000
10000
20000
30000
Index
C−FI3
Frequency
0
8000
Allele Frequency
Chr1
Frequency
0
2000
Allele Frequency
Chr2
Frequency
0
1500
Allele Frequency
Chr3
Frequency
0
1500
Allele Frequency
Chr4
Frequency
0
1000
Allele Frequency
Chr5
Frequency
0
1000
0.0
0.2
0.4
0.6
0.8
1.0
Allele Frequency

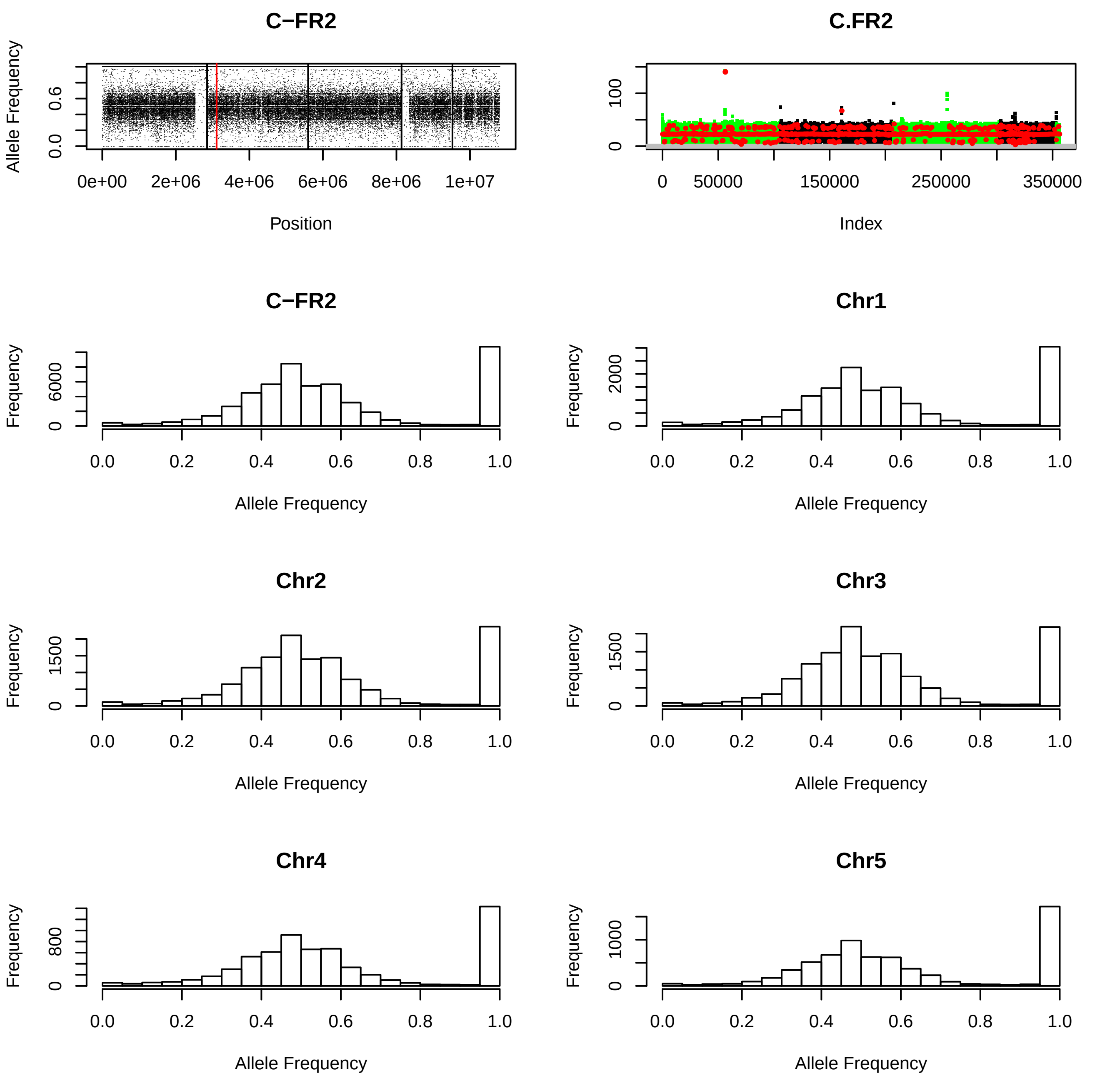
C−FR2
Allele Frequency
0.0
0.6
0e+00
2e+06
4e+06
6e+06
8e+06
1e+07
Position
C.FR2
0
100
0
50000
150000
250000
350000
Index
C−FR2
Frequency
0
6000
0.0
0.2
0.4
0.6
0.8
1.0
Allele Frequency
Chr1
Frequency
0
2000
0.0
0.2
0.4
0.6
0.8
1.0
Allele Frequency
Chr2
Frequency
0
1500
0.0
0.2
0.4
0.6
0.8
1.0
Allele Frequency
Chr3
Frequency
0
1500
0.0
0.2
0.4
0.6
0.8
1.0
Allele Frequency
Chr4
Frequency
0
800
0.0
0.2
0.4
0.6
0.8
1.0
Allele Frequency
Chr5
Frequency
0
1000
0.0
0.2
0.4
0.6
0.8
1.0
Allele Frequency

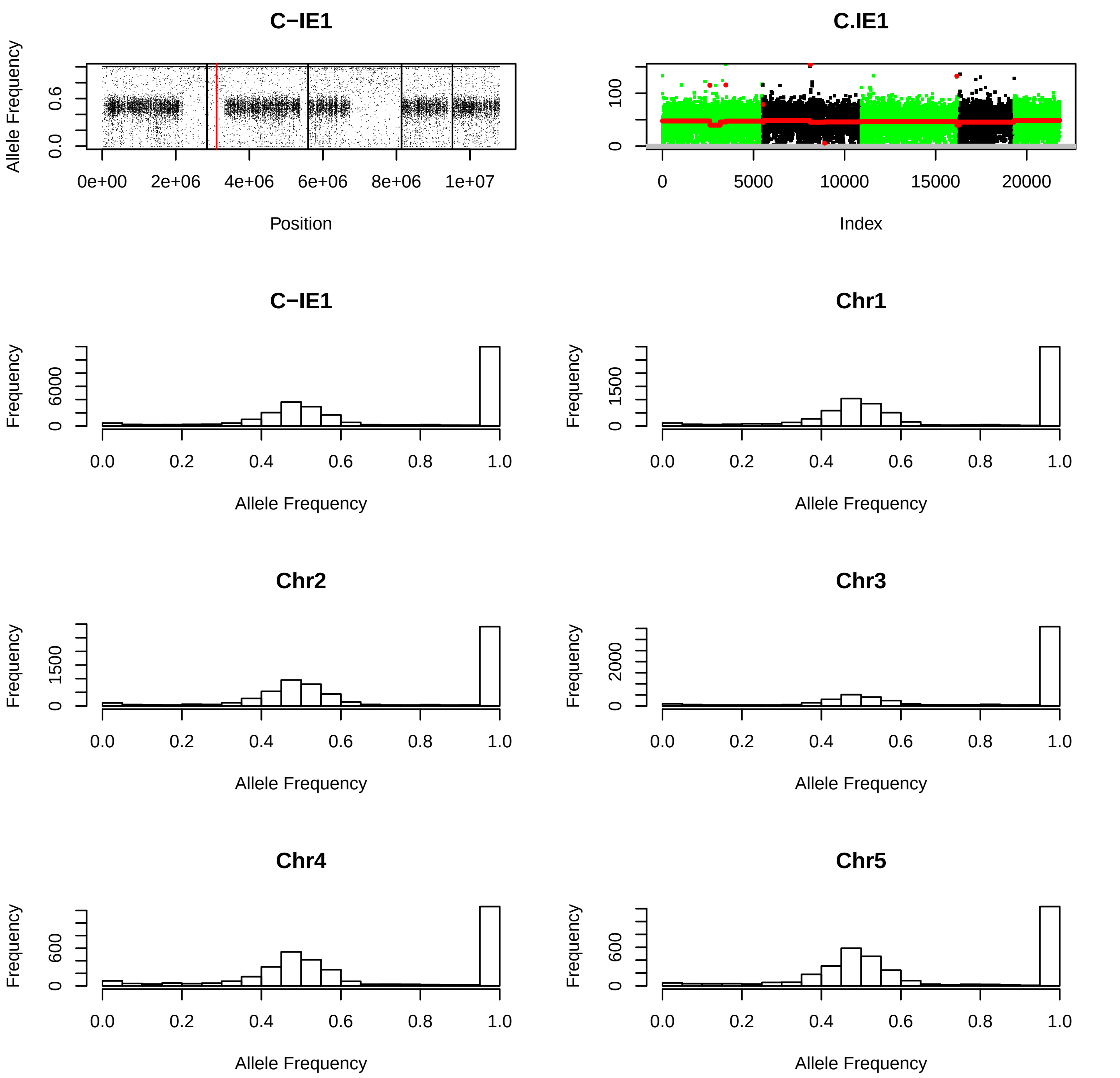
C−IE1
Allele Frequency
0.0
0.6
0e+00
2e+06
4e+06
6e+06
8e+06
1e+07
Position
C.IE1
0
100
0
5000
10000
15000
20000
Index
C−IE1
Frequency
0
6000
0.0
0.2
0.4
0.6
0.8
1.0
Allele Frequency
Chr1
Frequency
0
1500
Allele Frequency
Chr2
Frequency
0
1500
Allele Frequency
Chr3
Frequency
0
2000
Allele Frequency
Chr4
Frequency
0
600
Allele Frequency
Chr5
Frequency
0
600
Allele Frequency

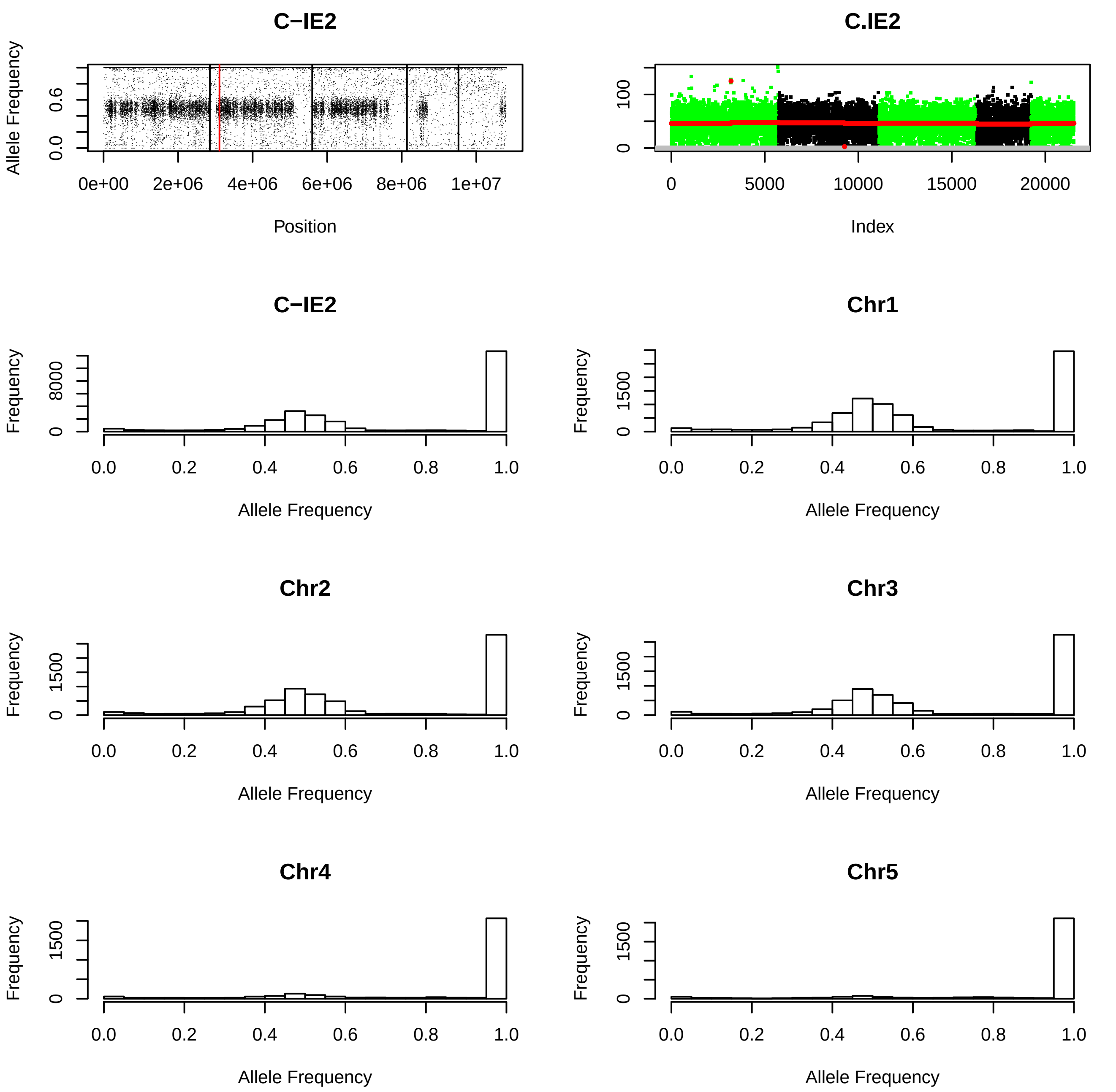
C−IE2
Allele Frequency
Position
C.IE2
Index
C−IE2
Frequency
Allele Frequency
Chr1
Chr2
Chr3
Chr4
Chr5

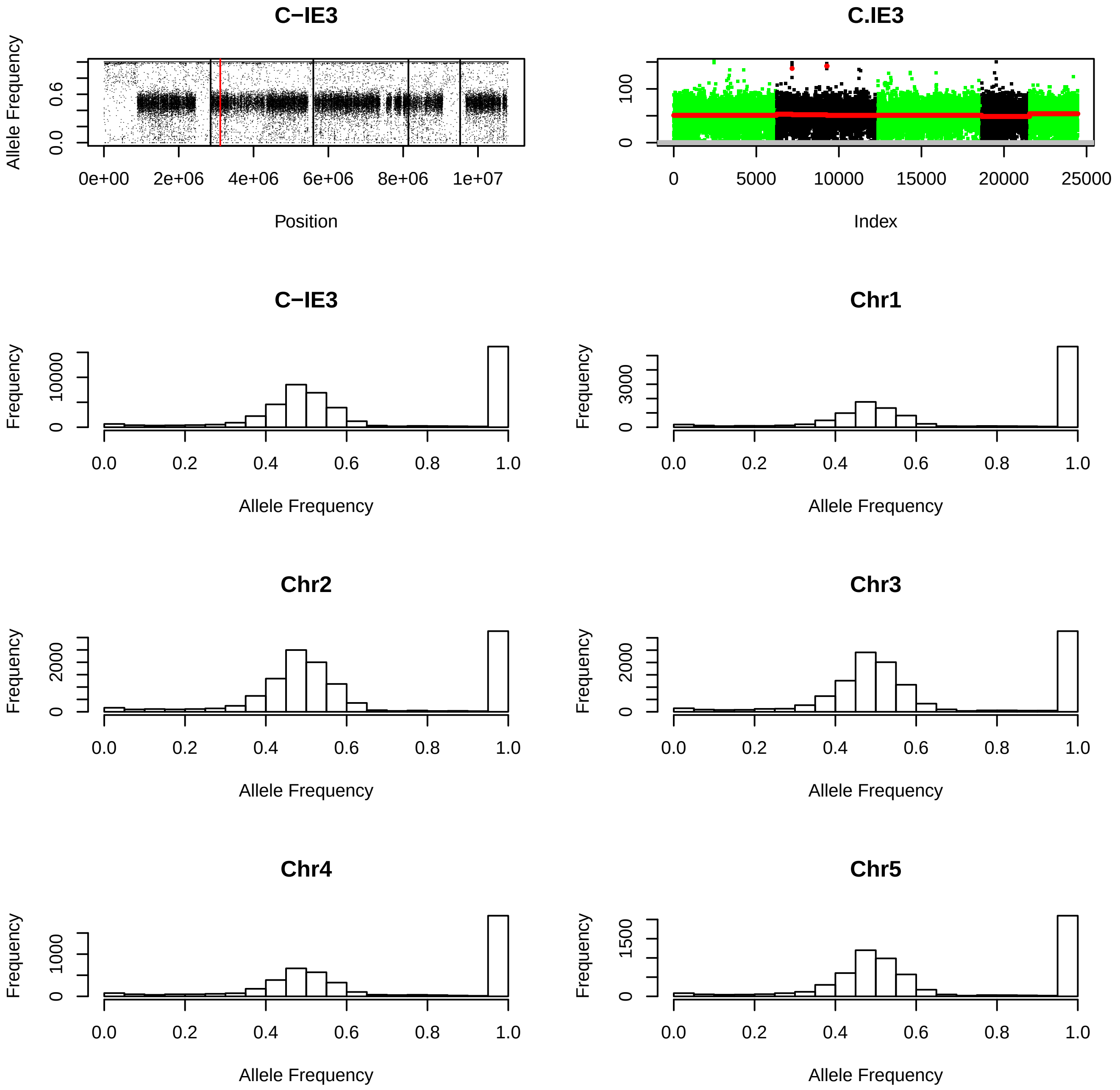
C−IE3
Allele Frequency
0.0
0.6
0e+00
2e+06
4e+06
6e+06
8e+06
1e+07
Position
C.IE3
0
100
0
5000
10000
15000
20000
25000
Index
C−IE3
Frequency
0
10000
0.0
0.2
0.4
0.6
0.8
1.0
Allele Frequency
Chr1
Frequency
0
3000
0.0
0.2
0.4
0.6
0.8
1.0
Allele Frequency
Chr2
Frequency
0
2000
0.0
0.2
0.4
0.6
0.8
1.0
Allele Frequency
Chr3
Frequency
0
2000
0.0
0.2
0.4
0.6
0.8
1.0
Allele Frequency
Chr4
Frequency
0
1000
0.0
0.2
0.4
0.6
0.8
1.0
Allele Frequency
Chr5
Frequency
0
1500
0.0
0.2
0.4
0.6
0.8
1.0
Allele Frequency

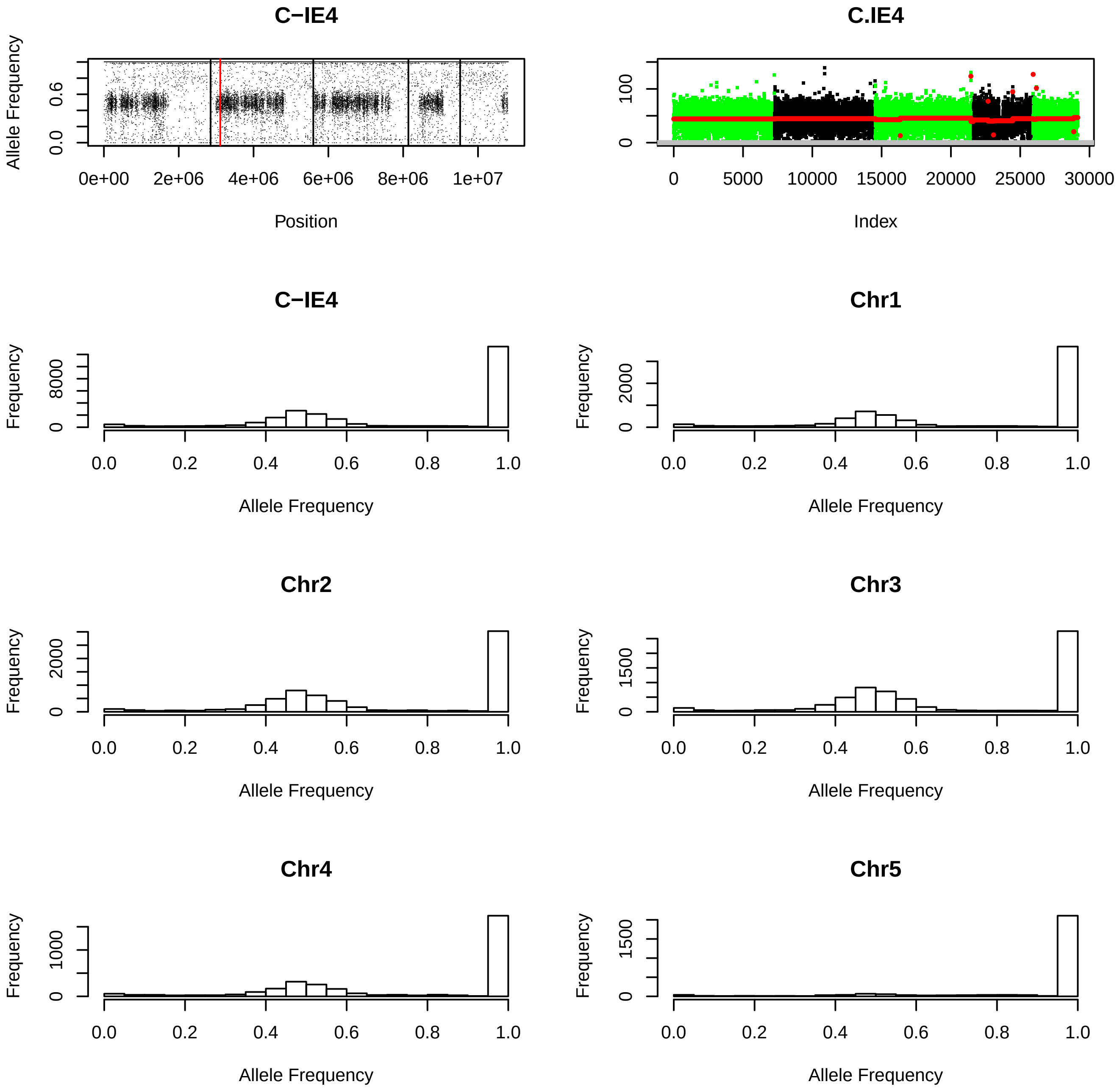
C−IE4
Allele Frequency
0.0
0.6
0e+00
2e+06
4e+06
6e+06
8e+06
1e+07
Position
C.IE4
0
100
0
5000
10000
15000
20000
25000
30000
Index
C−IE4
Frequency
0
8000
0.0
0.2
0.4
0.6
0.8
1.0
Allele Frequency
Chr1
Frequency
0
2000
0.0
0.2
0.4
0.6
0.8
1.0
Allele Frequency
Chr2
Frequency
0
2000
0.0
0.2
0.4
0.6
0.8
1.0
Allele Frequency
Chr3
Frequency
0
1500
0.0
0.2
0.4
0.6
0.8
1.0
Allele Frequency
Chr4
Frequency
0
1000
0.0
0.2
0.4
0.6
0.8
1.0
Allele Frequency
Chr5
Frequency
0
1500
0.0
0.2
0.4
0.6
0.8
1.0
Allele Frequency

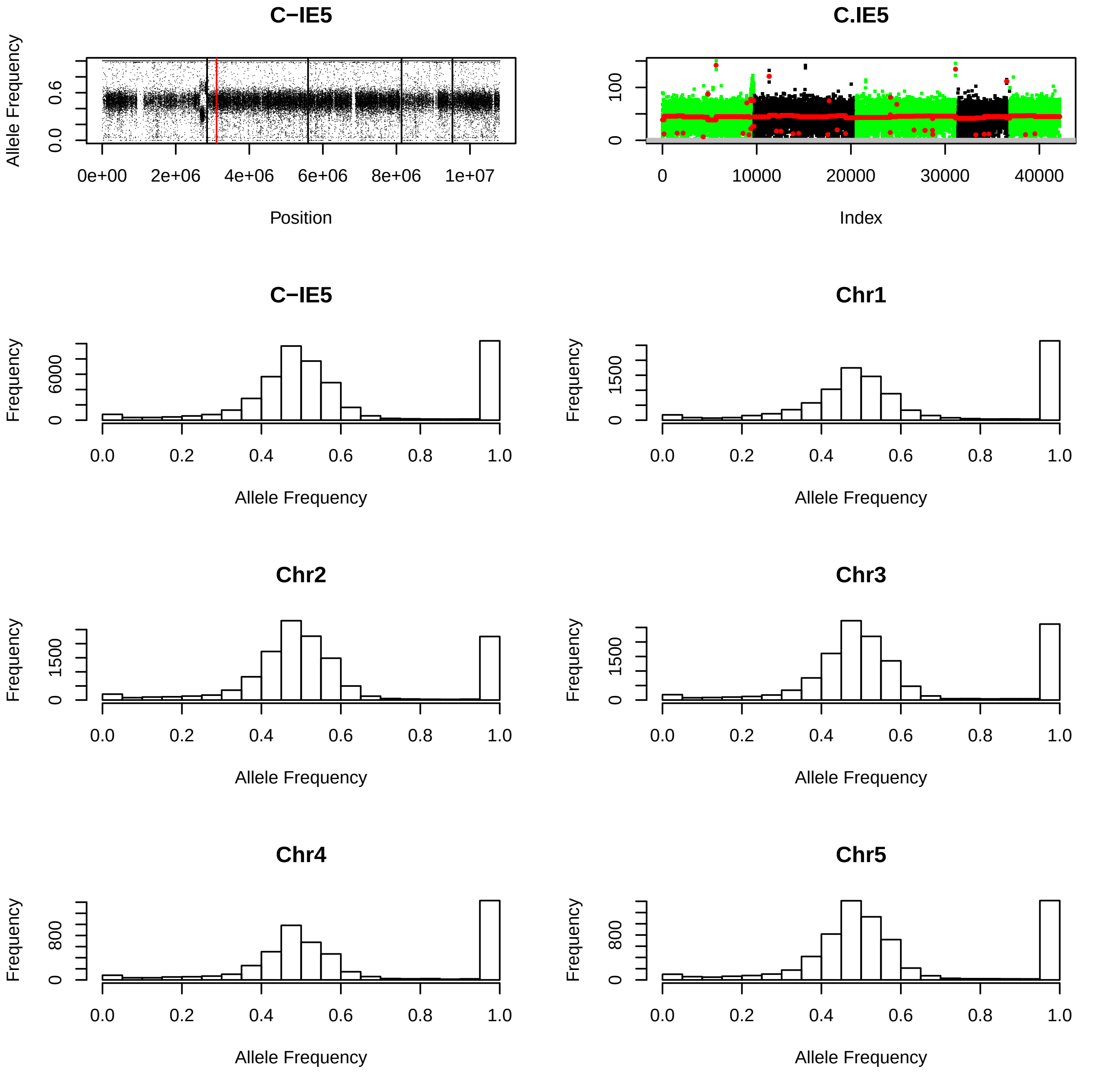

C−IE5
Allele Frequency
0.0
0.6
0e+00
2e+06
4e+06
6e+06
8e+06
1e+07
Position
C.IE5
0
100
0
10000
20000
30000
40000
Index
C−IE5
Frequency
0
6000
Allele Frequency
Chr1
0
1500
Chr2
Chr3
Chr4
800
Chr5
0.0
0.2
0.4
0.6
0.8
1.0

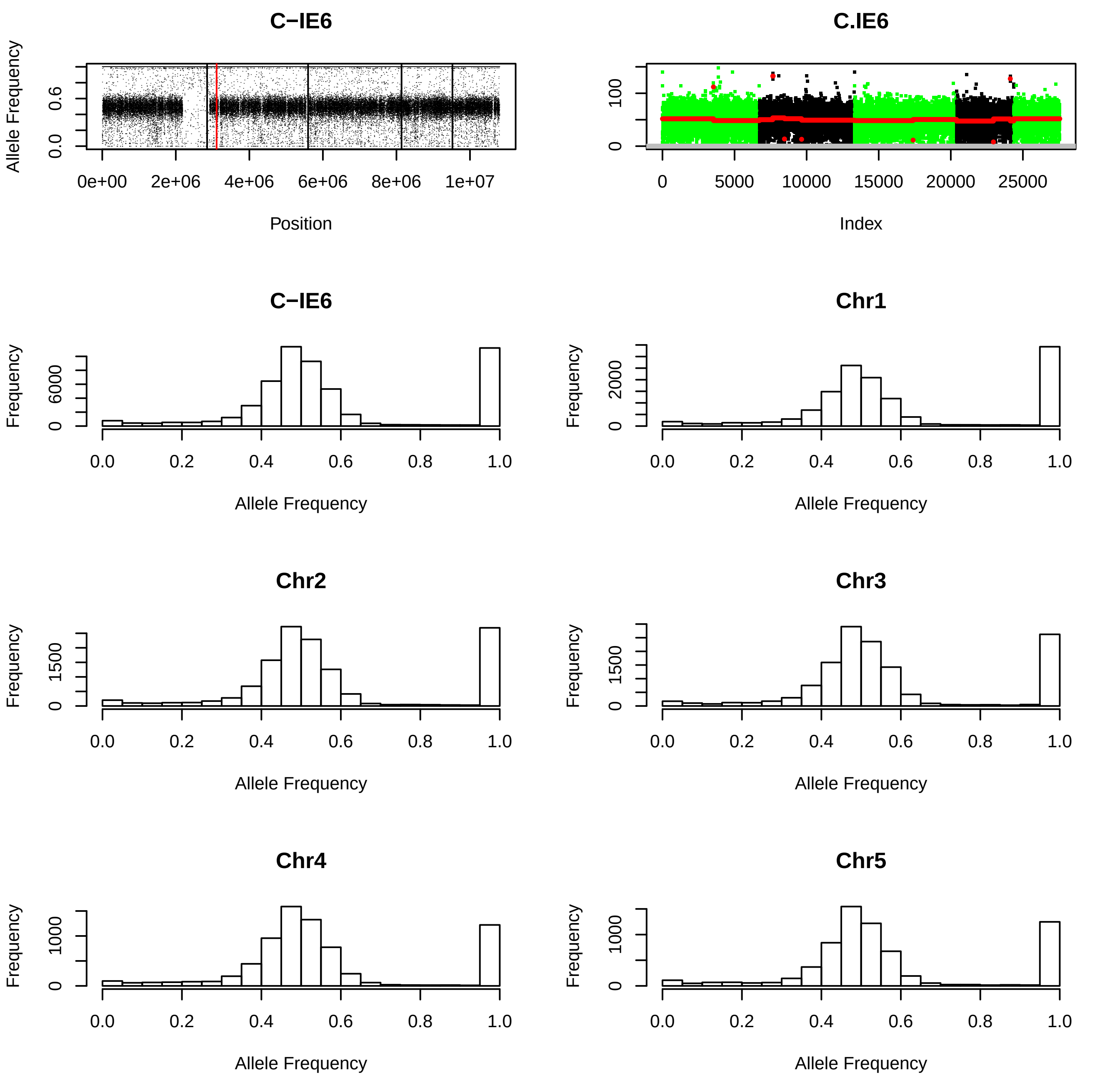
C−IE6
Allele Frequency
0.0
0.6
0e+00
2e+06
4e+06
6e+06
8e+06
1e+07
Position
C.IE6
0
100
0
5000
10000
15000
20000
25000
Index
C−IE6
Frequency
0
6000
0.0
0.2
0.4
0.6
0.8
1.0
Allele Frequency
Chr1
Frequency
0
2000
0.0
0.2
0.4
0.6
0.8
1.0
Allele Frequency
Chr2
Frequency
0
1500
0.0
0.2
0.4
0.6
0.8
1.0
Allele Frequency
Chr3
Frequency
0
1500
0.0
0.2
0.4
0.6
0.8
1.0
Allele Frequency
Chr4
Frequency
0
1000
0.0
0.2
0.4
0.6
0.8
1.0
Allele Frequency
Chr5
Frequency
0
1000
0.0
0.2
0.4
0.6
0.8
1.0
Allele Frequency

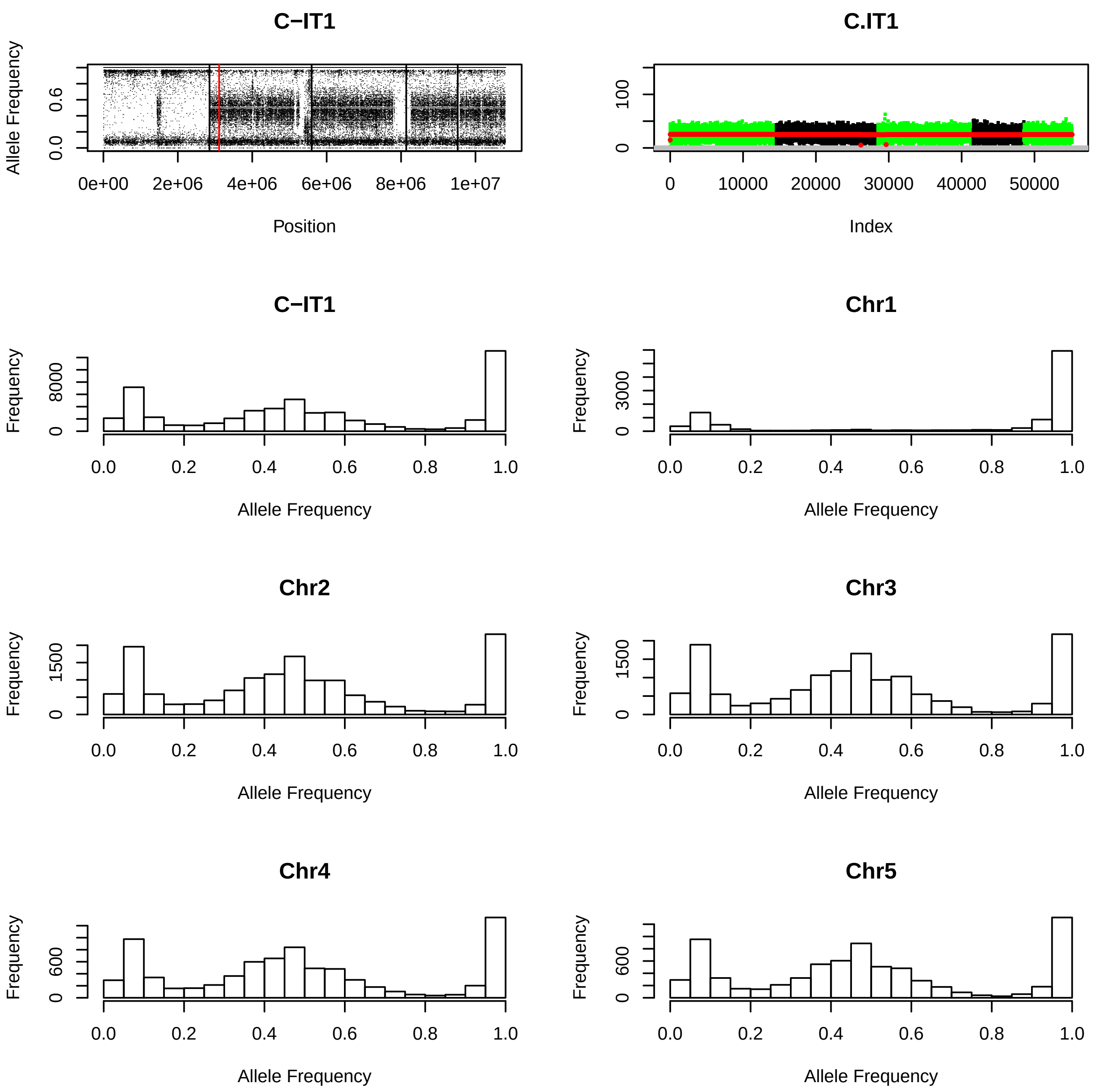
C−IT1
Allele Frequency
0.0
0.6
0e+00
2e+06
4e+06
6e+06
8e+06
1e+07
Position
C.IT1
0
100
0
10000
20000
30000
40000
50000
Index
C−IT1
Frequency
0
8000
Allele Frequency
Chr1
Frequency
0
3000
Allele Frequency
Chr2
Frequency
0
1500
Allele Frequency
Chr3
Frequency
0
1500
Allele Frequency
Chr4
Frequency
0
600
Allele Frequency
Chr5
Frequency
0
600
Allele Frequency
0.0
0.2
0.4
0.6
0.8
1.0

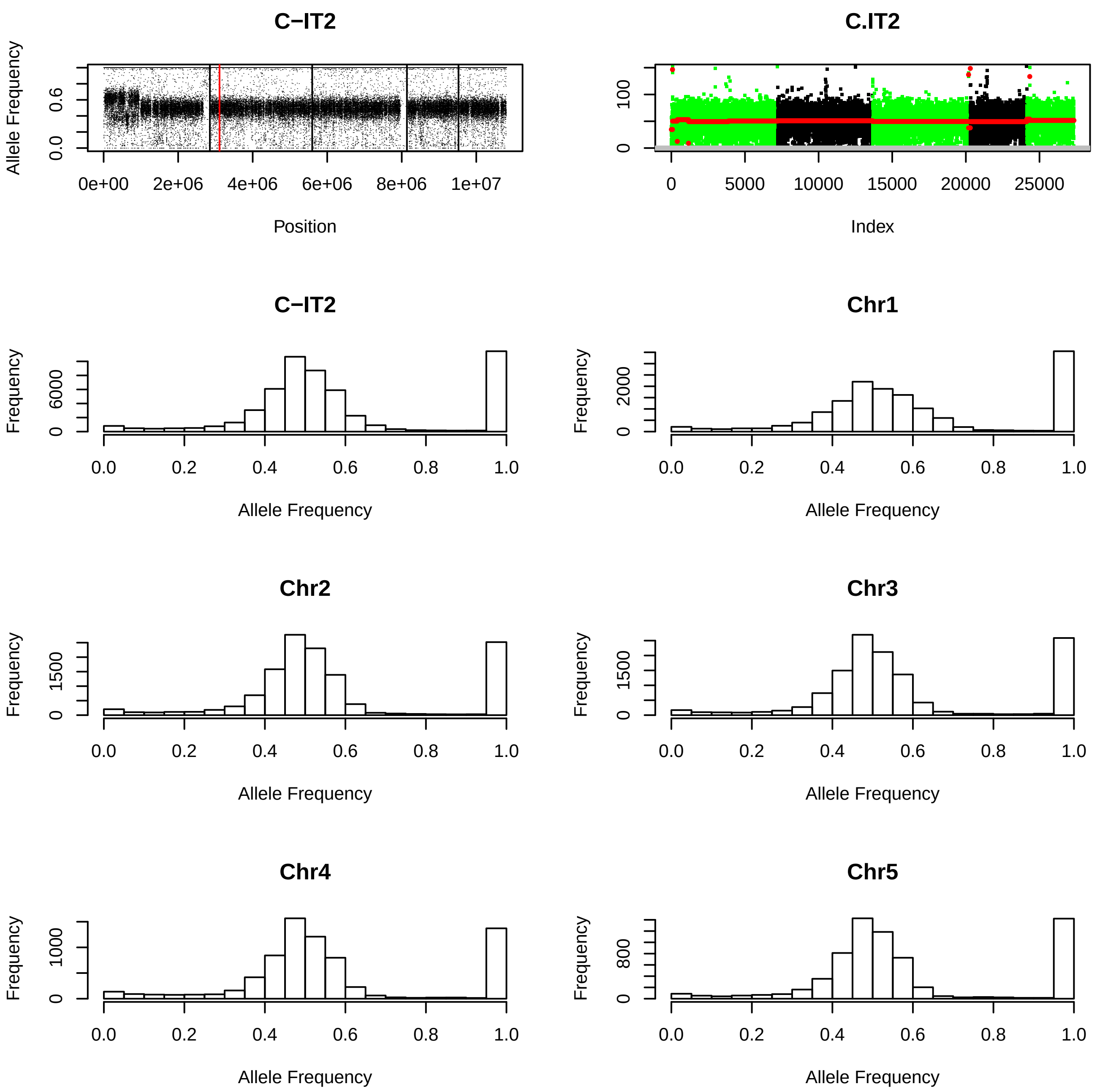
C−IT2
Allele Frequency
0.0
0.6
0e+00
2e+06
4e+06
6e+06
8e+06
1e+07
Position
C.IT2
0
100
0
5000
10000
15000
20000
25000
Index
C−IT2
Frequency
0
6000
0.0
0.2
0.4
0.6
0.8
1.0
Allele Frequency
Chr1
Frequency
0
2000
0.0
0.2
0.4
0.6
0.8
1.0
Allele Frequency
Chr2
Frequency
0
1500
0.0
0.2
0.4
0.6
0.8
1.0
Allele Frequency
Chr3
Frequency
0
1500
0.0
0.2
0.4
0.6
0.8
1.0
Allele Frequency
Chr4
Frequency
0
1000
0.0
0.2
0.4
0.6
0.8
1.0
Allele Frequency
Chr5
Frequency
0
800
0.0
0.2
0.4
0.6
0.8
1.0
Allele Frequency

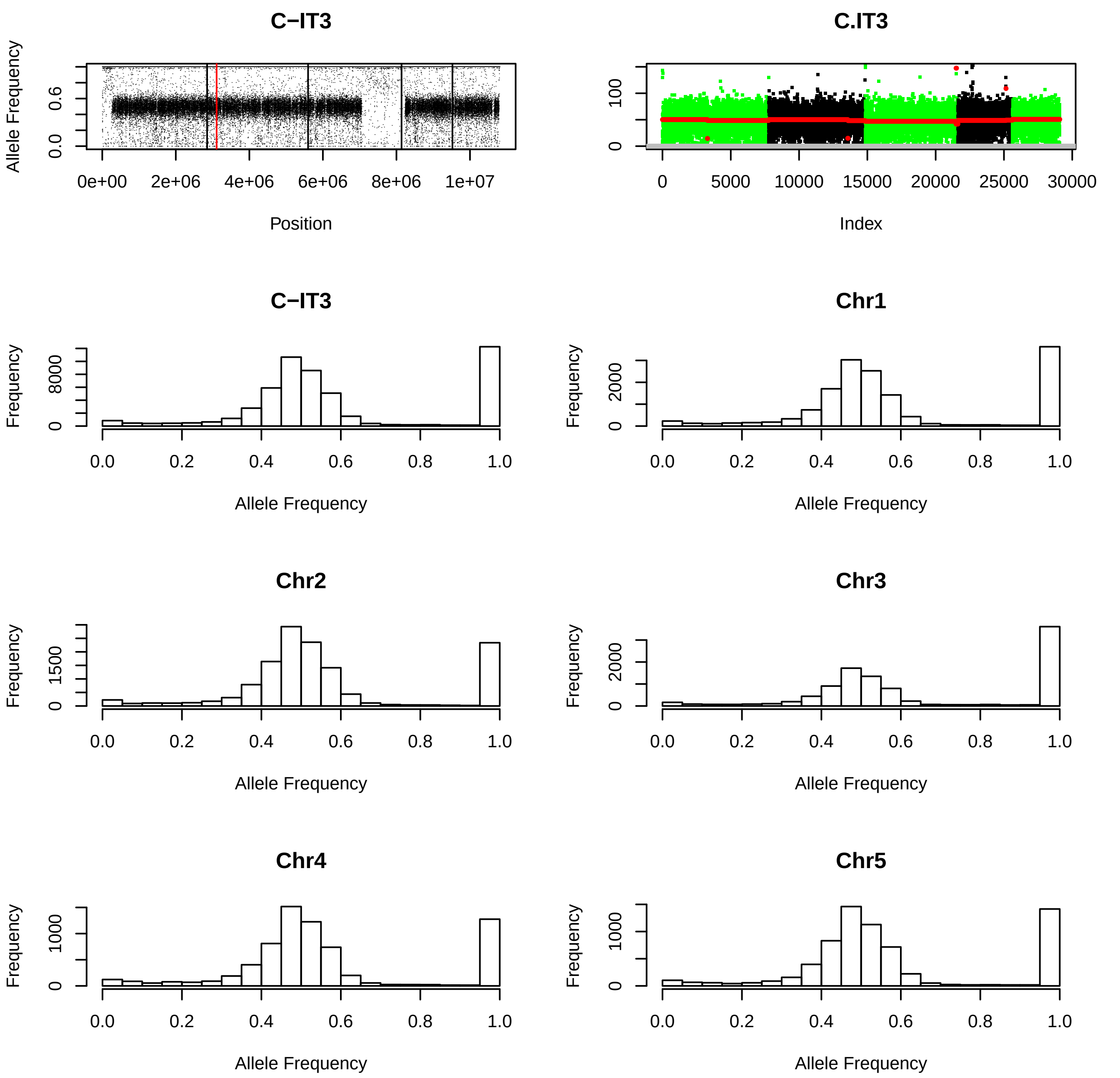
C−IT3
Allele Frequency
0.0
0.6
0e+00
2e+06
4e+06
6e+06
8e+06
1e+07
Position
C.IT3
0
100
0
5000
10000
15000
20000
25000
30000
Index
C−IT3
Frequency
0
8000
0.0
0.2
0.4
0.6
0.8
1.0
Allele Frequency
Chr1
Frequency
0
2000
0.0
0.2
0.4
0.6
0.8
1.0
Allele Frequency
Chr2
Frequency
0
1500
0.0
0.2
0.4
0.6
0.8
1.0
Allele Frequency
Chr3
Frequency
0
2000
0.0
0.2
0.4
0.6
0.8
1.0
Allele Frequency
Chr4
Frequency
0
1000
0.0
0.2
0.4
0.6
0.8
1.0
Allele Frequency
Chr5
Frequency
0
1000
0.0
0.2
0.4
0.6
0.8
1.0
Allele Frequency

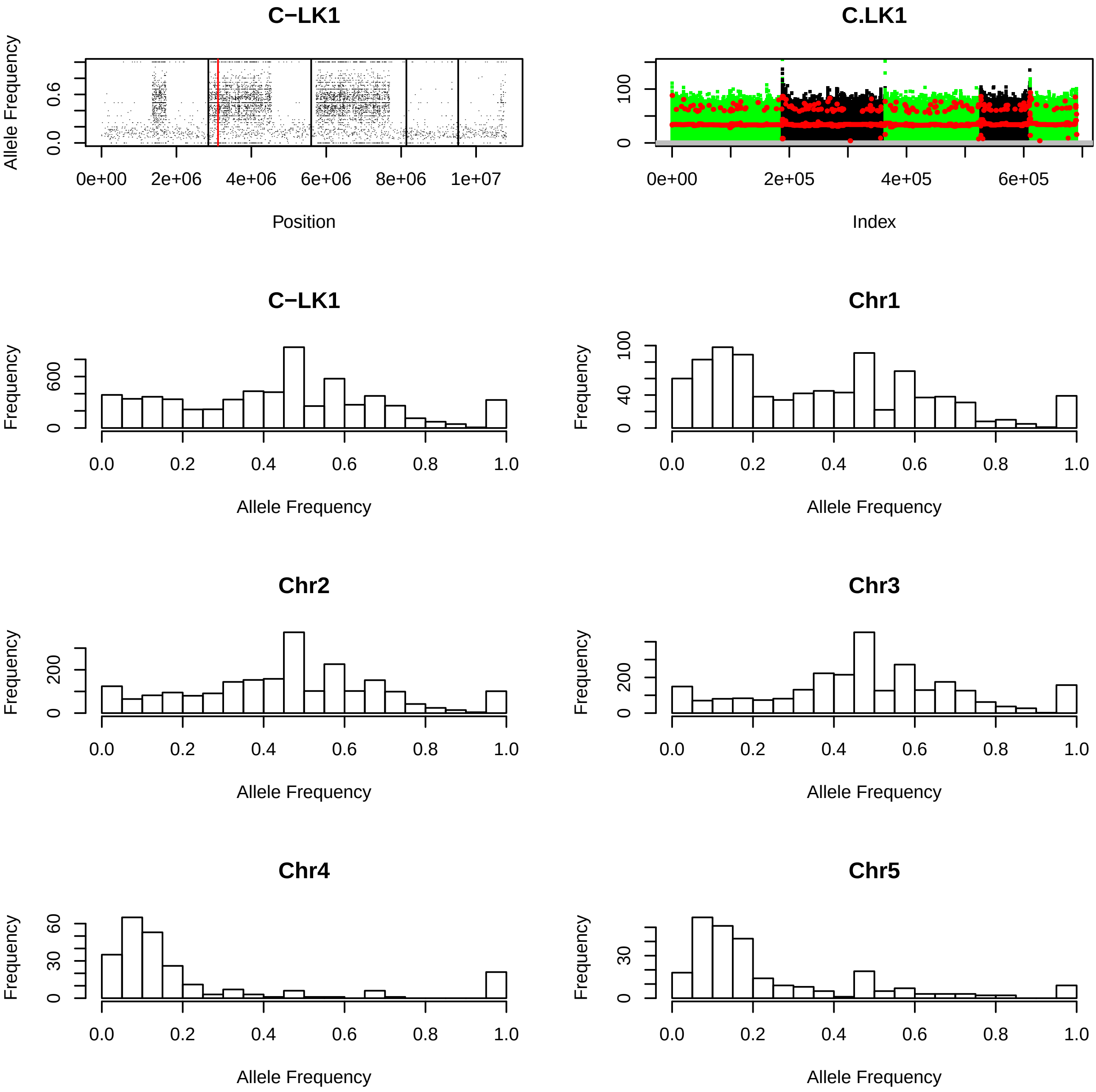
C−LK1
Allele Frequency
0.0
0.6
0e+00
2e+06
4e+06
6e+06
8e+06
1e+07
Position
C.LK1
0
100
0e+00
2e+05
4e+05
6e+05
Index
C−LK1
Frequency
0
600
0.0
0.2
0.4
0.6
0.8
1.0
Allele Frequency
Chr1
Frequency
0
40
100
Allele Frequency
Chr2
Frequency
0
200
Allele Frequency
Chr3
Frequency
0
200
Allele Frequency
Chr4
Frequency
0
30
60
Allele Frequency
Chr5
Frequency
0
30
Allele Frequency

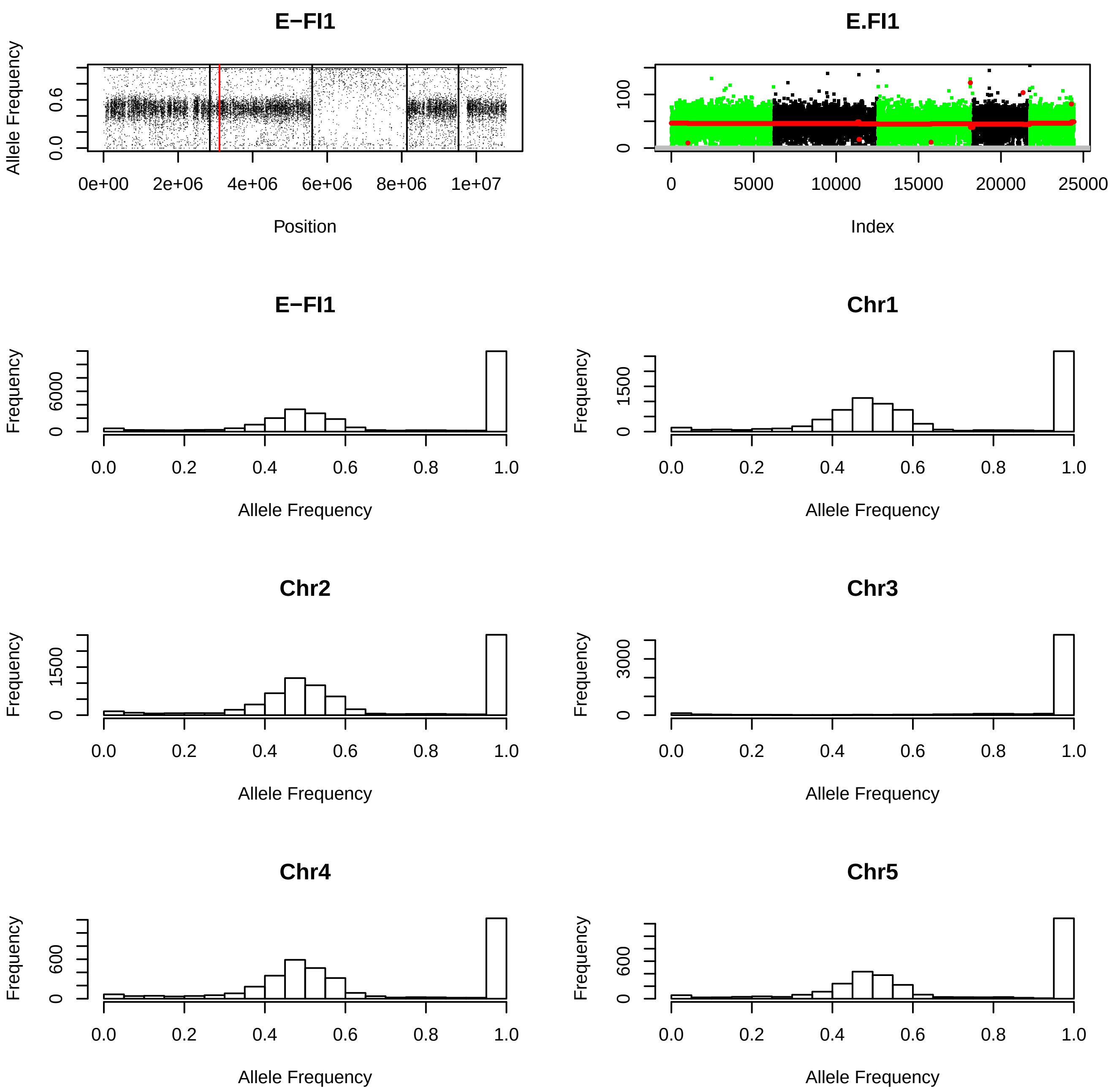
E−Fl1
Allele Frequency
0.0
0.6
0e+00
2e+06
4e+06
6e+06
8e+06
1e+07
Position
E.Fl1
0
100
0
5000
10000
15000
20000
25000
Index
E−Fl1
Frequency
0
6000
0.0
0.2
0.4
0.6
0.8
1.0
Allele Frequency
Chr1
Frequency
0
1500
Allele Frequency
Chr2
Frequency
0
1500
Allele Frequency
Chr3
Frequency
0
3000
Allele Frequency
Chr4
Frequency
0
600
Allele Frequency
Chr5
Frequency
0
600
Allele Frequency

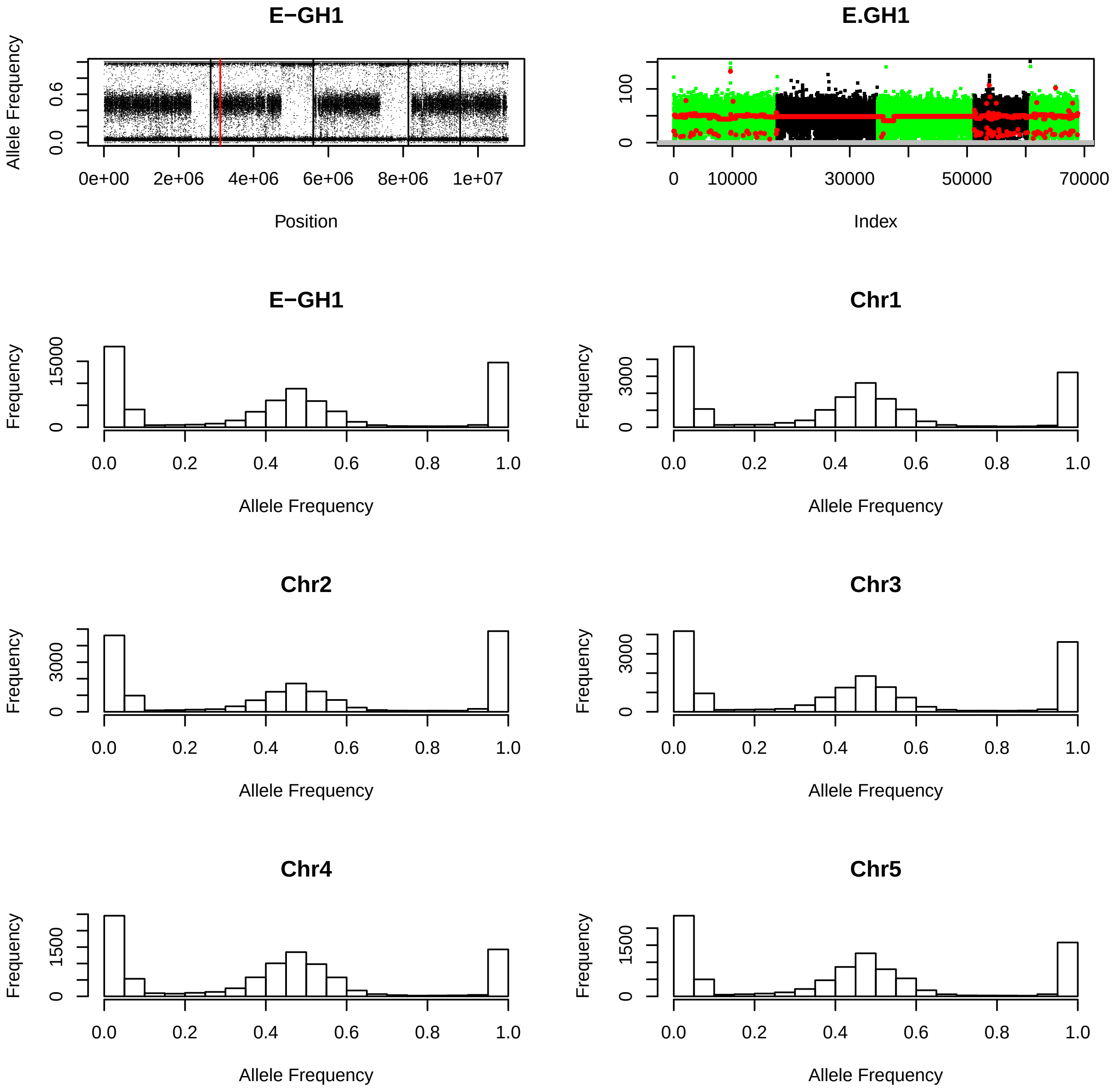
E−GH1
Allele Frequency
0.0
0.6
0e+00
2e+06
4e+06
6e+06
8e+06
1e+07
Position
E.GH1
0
100
0
10000
30000
50000
70000
Index
E−GH1
Frequency
0
15000
0.0
0.2
0.4
0.6
0.8
1.0
Allele Frequency
Chr1
Frequency
0
3000
Allele Frequency
Chr2
Frequency
0
3000
Allele Frequency
Chr3
Frequency
0
3000
Allele Frequency
Chr4
Frequency
0
1500
Allele Frequency
Chr5
Frequency
0
1500
Allele Frequency

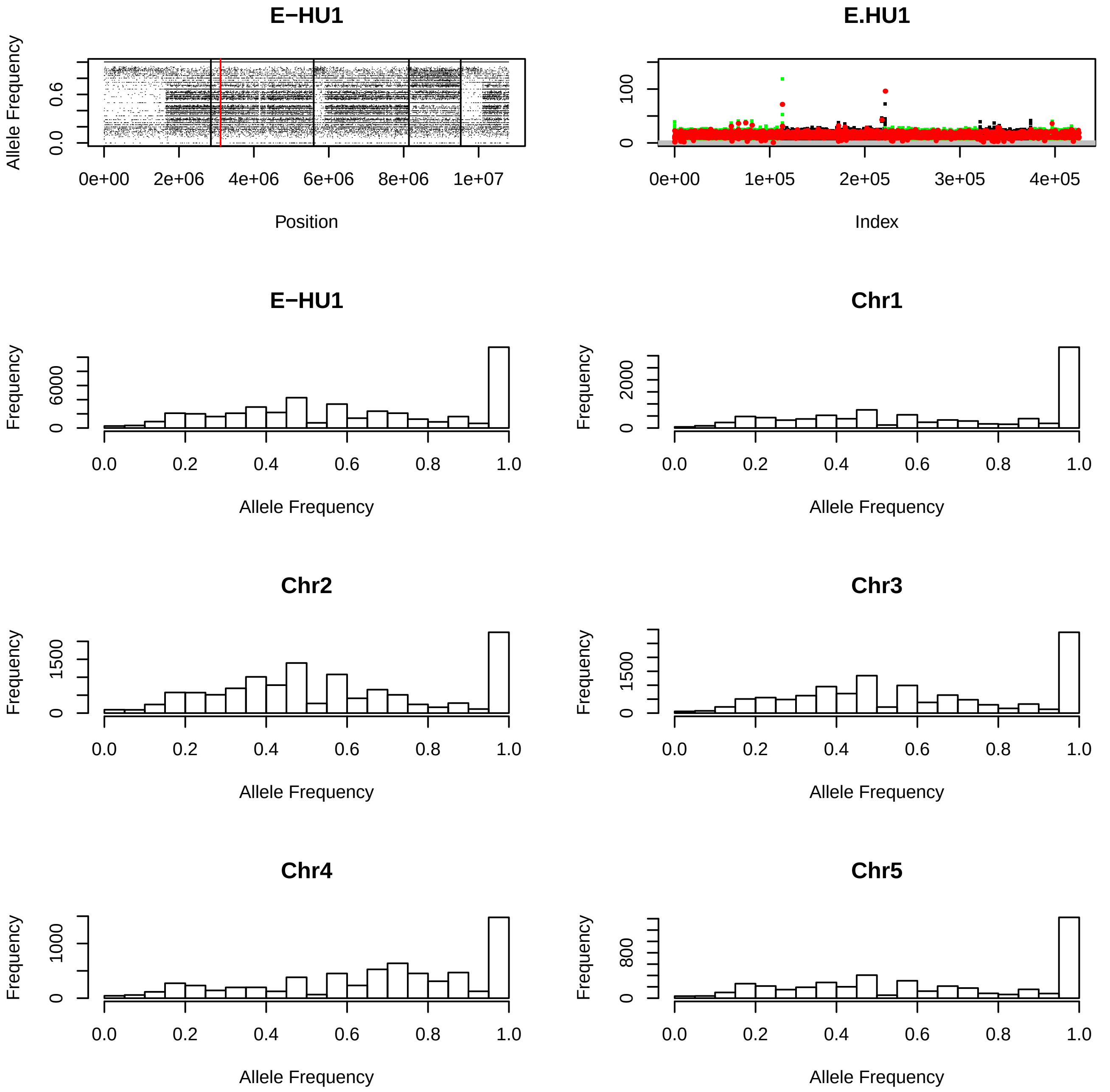
E−HU1
Allele Frequency
0.0
0.6
0e+00
2e+06
4e+06
6e+06
8e+06
1e+07
Position
E.HU1
0
100
0e+00
1e+05
2e+05
3e+05
4e+05
Index
E−HU1
Frequency
0
6000
0.0
0.2
0.4
0.6
0.8
1.0
Allele Frequency
Chr1
Frequency
0
2000
Allele Frequency
Chr2
Frequency
0
1500
Allele Frequency
Chr3
Frequency
0
1500
Allele Frequency
Chr4
Frequency
0
1000
Allele Frequency
Chr5
Frequency
0
800
Allele Frequency

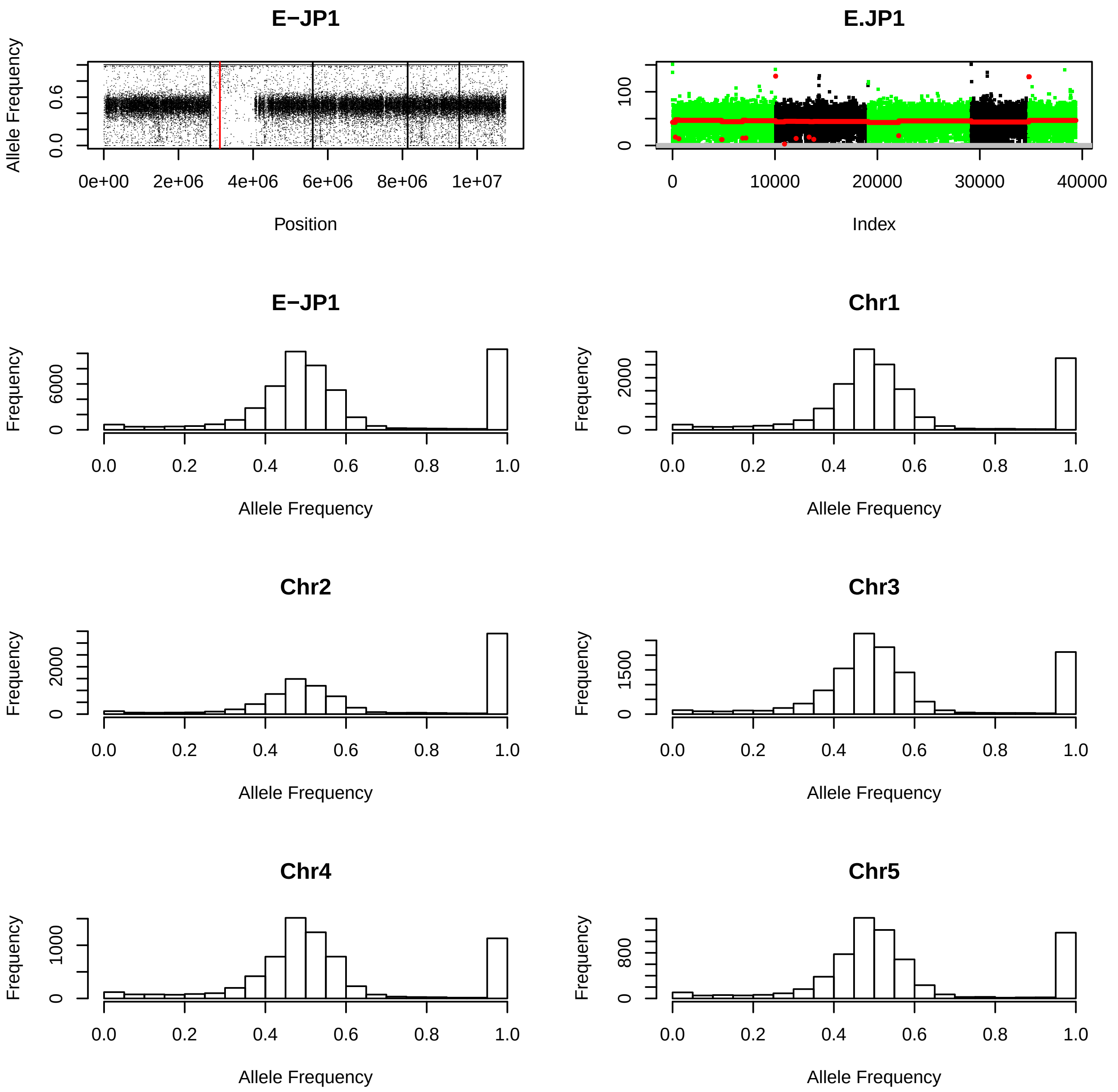
E−JP1
Allele Frequency
0.0
0.6
0e+00
2e+06
4e+06
6e+06
8e+06
1e+07
Position
E.JP1
0
100
0
10000
20000
30000
40000
Index
E−JP1
Frequency
0
6000
Allele Frequency
Chr1
Frequency
0
2000
Allele Frequency
Chr2
Frequency
0
2000
Allele Frequency
Chr3
Frequency
0
1500
Allele Frequency
Chr4
Frequency
0
1000
Allele Frequency
Chr5
Frequency
0
800
Allele Frequency
0.0
0.2
0.4
0.6
0.8
1.0

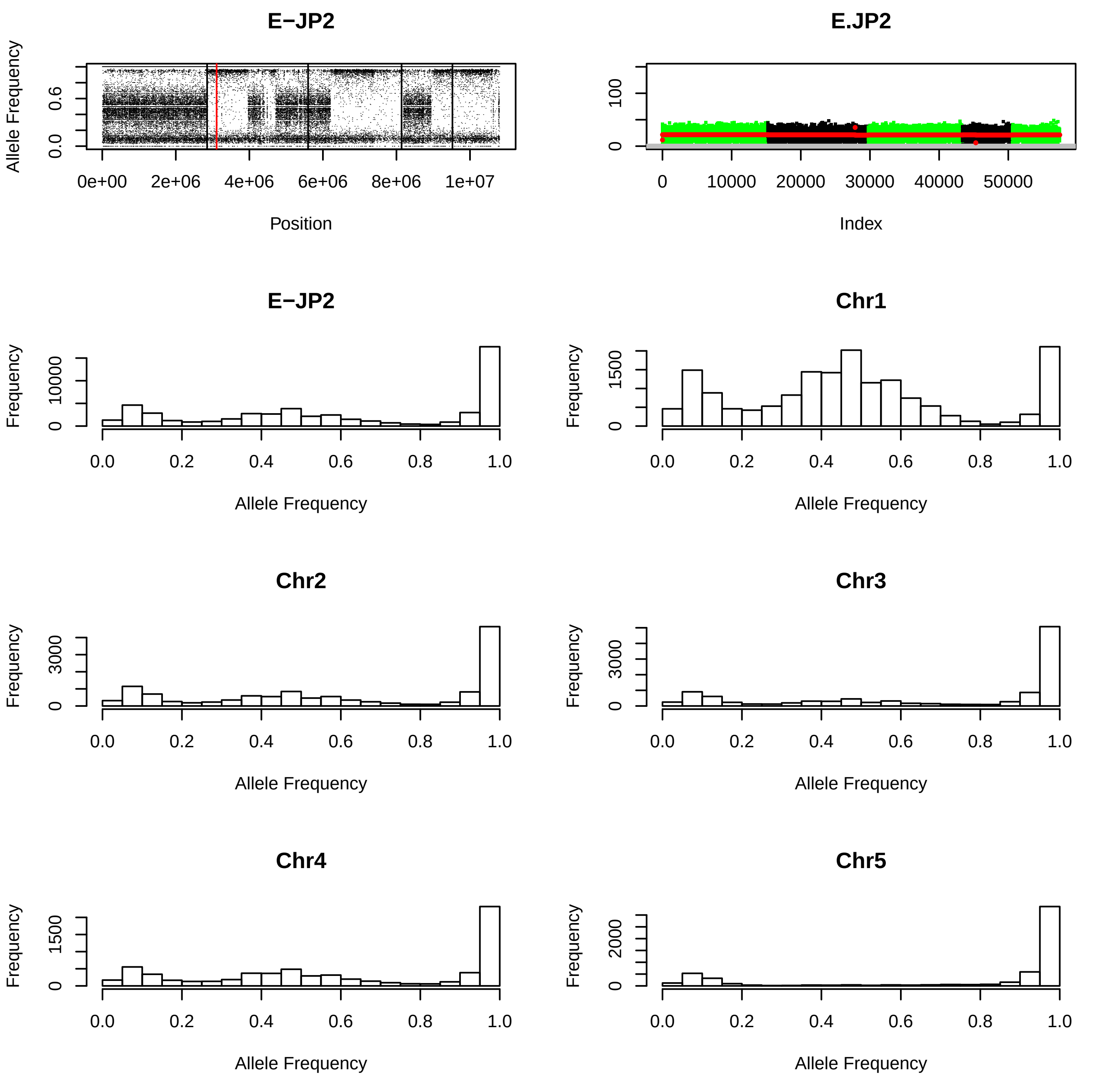
E−JP2
Allele Frequency
0.0
0.6
0e+00
2e+06
4e+06
6e+06
8e+06
1e+07
Position
E.JP2
0
100
0
10000
20000
30000
40000
50000
Index
E−JP2
Frequency
0
10000
0.0 0.2 0.4 0.6 0.8 1.0
Allele Frequency
Chr1
Frequency
0
1500
0.0 0.2 0.4 0.6 0.8 1.0
Allele Frequency
Chr2
Frequency
0
3000
0.0 0.2 0.4 0.6 0.8 1.0
Allele Frequency
Chr3
Frequency
0
3000
0.0 0.2 0.4 0.6 0.8 1.0
Allele Frequency
Chr4
Frequency
0
1500
0.0 0.2 0.4 0.6 0.8 1.0
Allele Frequency
Chr5
Frequency
0
2000
0.0 0.2 0.4 0.6 0.8 1.0
Allele Frequency

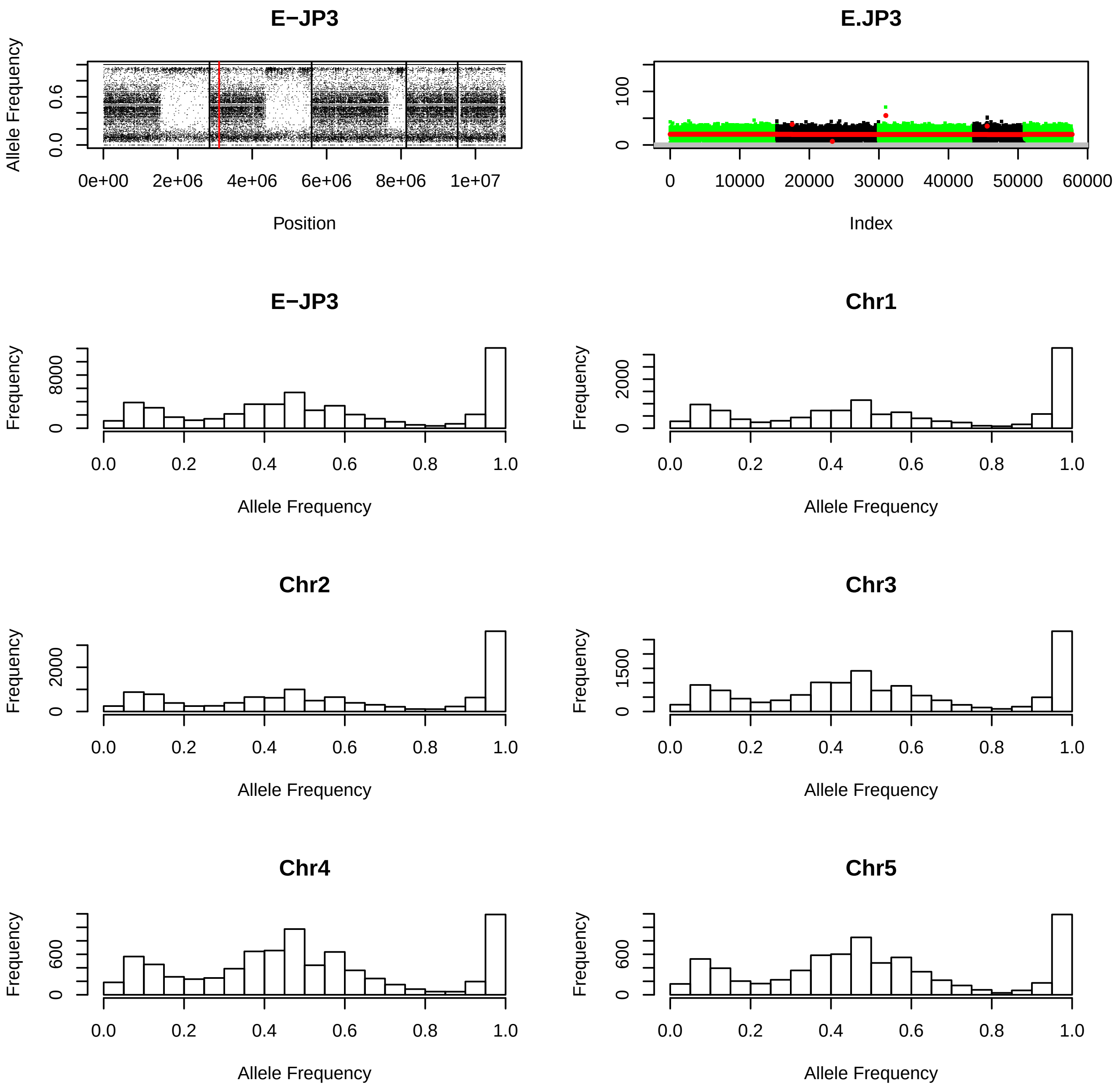
E−JP3
Allele Frequency
0.0
0.6
0e+00
2e+06
4e+06
6e+06
8e+06
1e+07
Position
E.JP3
0
100
0
10000
20000
30000
40000
50000
60000
Index
E−JP3
Chr1
Chr2
Chr3
Chr4
Chr5
Frequency
Allele Frequency
0.0
0.2
0.4
0.6
0.8
1.0
8000
2000
1500
600

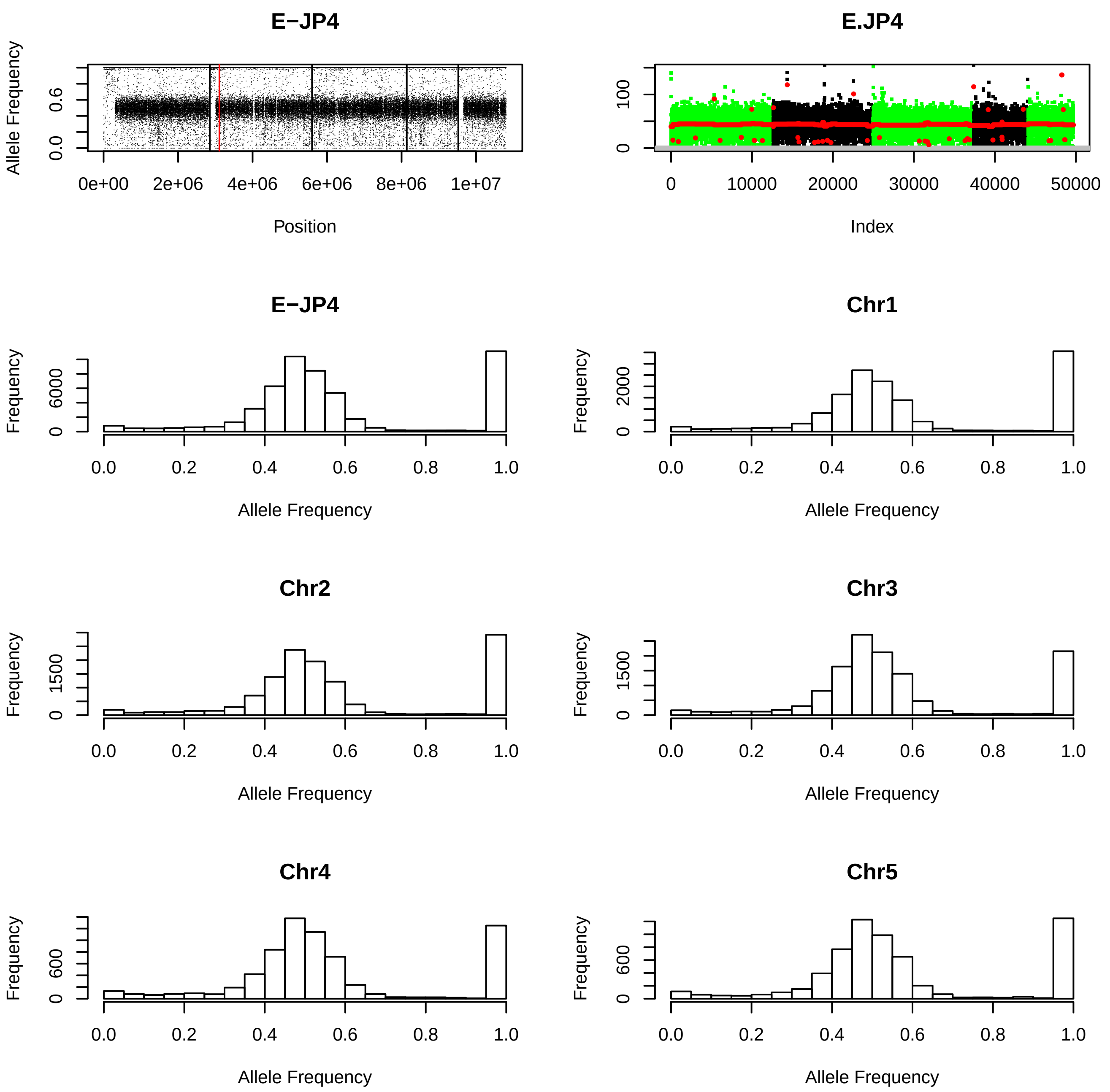
E−JP4
Allele Frequency
0.0
0.6
0e+00
2e+06
4e+06
6e+06
8e+06
1e+07
Position
E.JP4
0
100
0
10000
20000
30000
40000
50000
Index
E−JP4
Frequency
0
6000
0.0
0.2
0.4
0.6
0.8
1.0
Allele Frequency
Chr1
Frequency
0
2000
Allele Frequency
Chr2
Frequency
0
1500
Allele Frequency
Chr3
Frequency
0
1500
Allele Frequency
Chr4
Frequency
0
600
Allele Frequency
Chr5
Frequency
0
600
Allele Frequency

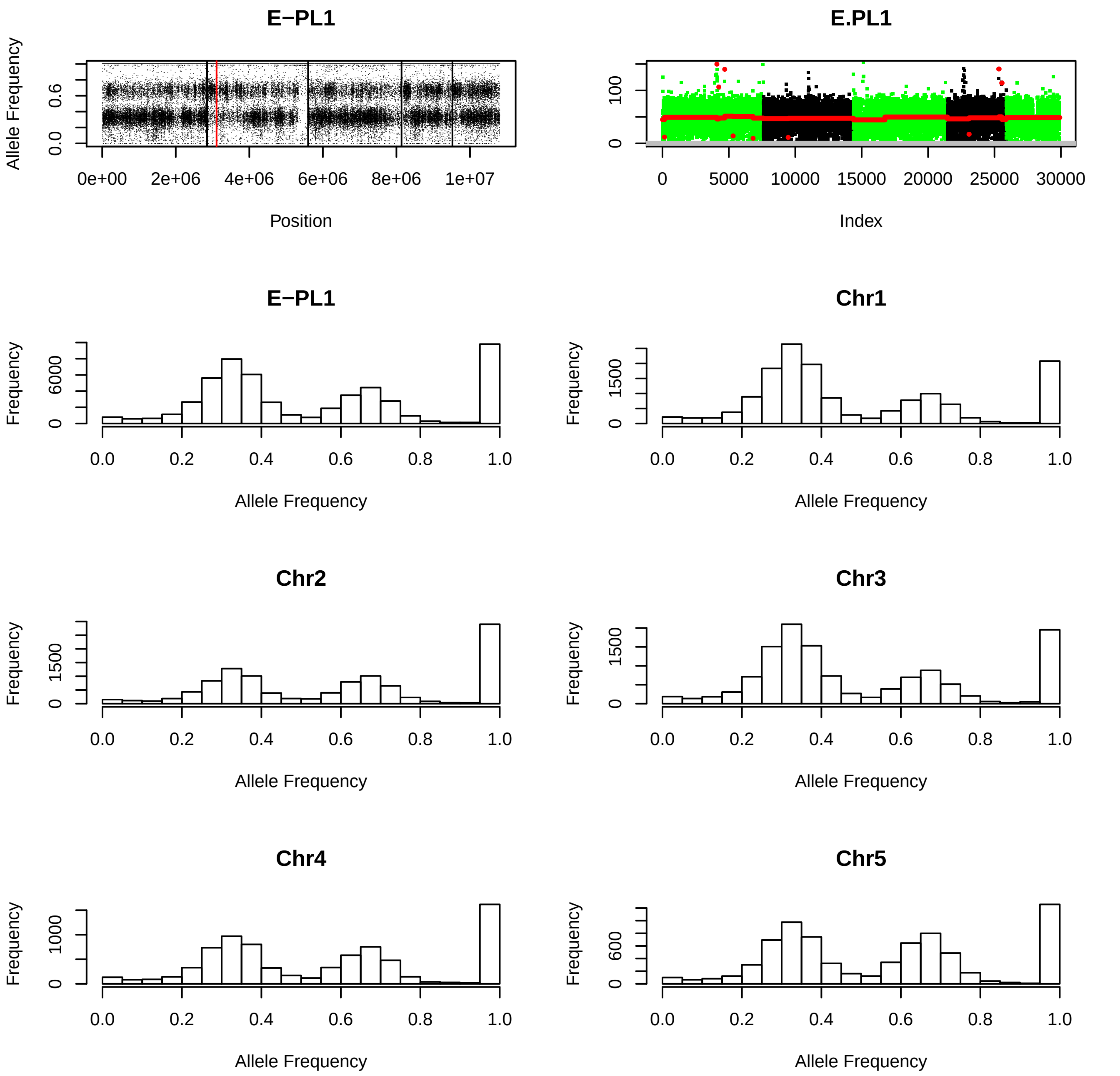
E−PL1
Allele Frequency
0.0
0.6
0e+00
2e+06
4e+06
6e+06
8e+06
1e+07
Position
E.PL1
0
100
0
5000
10000
15000
20000
25000
30000
Index
E−PL1
Frequency
0
6000
0.0
0.2
0.4
0.6
0.8
1.0
Allele Frequency
Chr1
Frequency
0
1500
Allele Frequency
Chr2
Frequency
0
1500
Allele Frequency
Chr3
Frequency
0
1500
Allele Frequency
Chr4
Frequency
0
1000
Allele Frequency
Chr5
Frequency
0
600
Allele Frequency

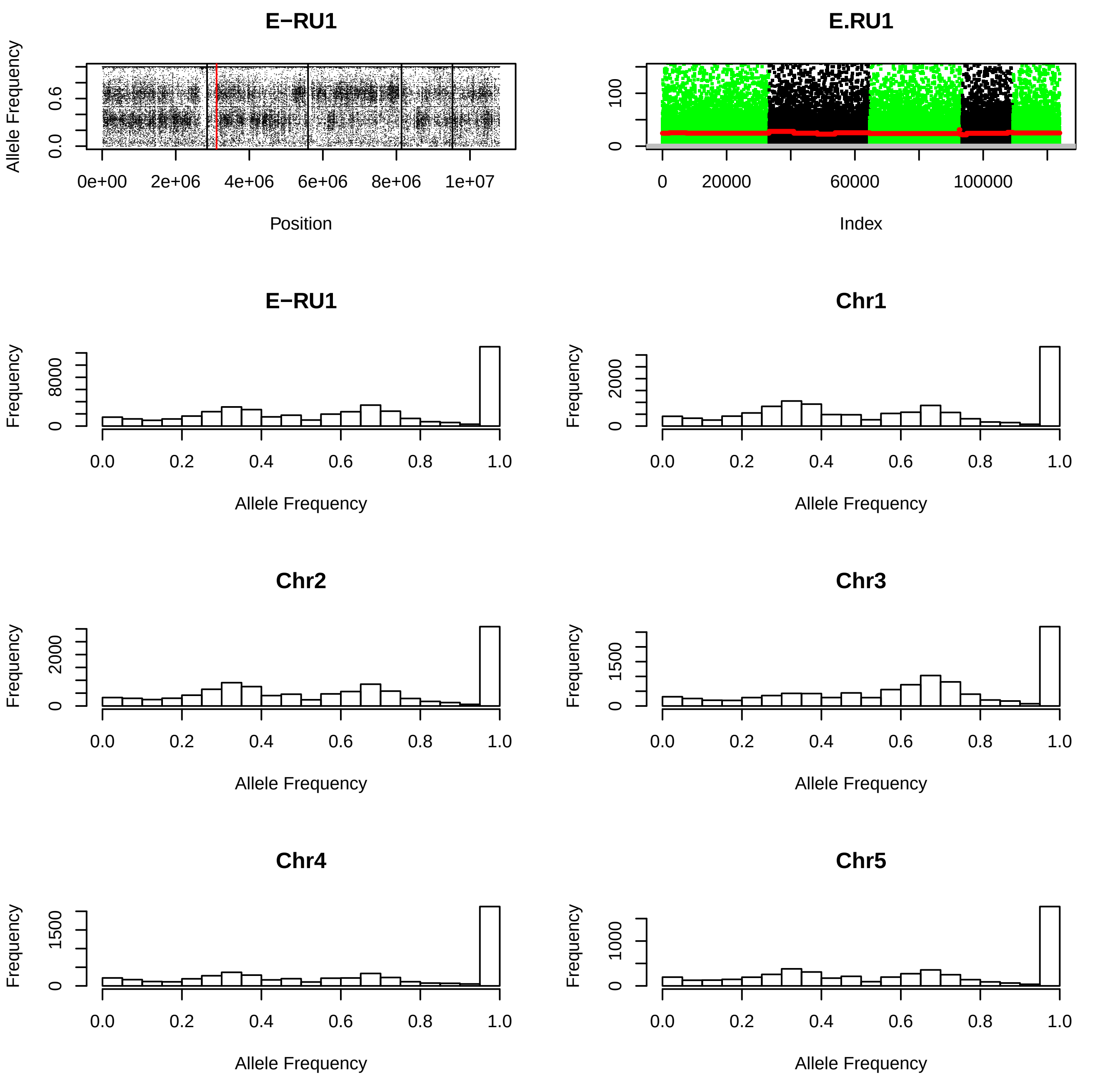
E−RU1
Allele Frequency
0.0
0.6
0e+00
2e+06
4e+06
6e+06
8e+06
1e+07
Position
E.RU1
0
100
0
20000
60000
100000
Index
E−RU1
Frequency
0
8000
0.0
0.2
0.4
0.6
0.8
1.0
Allele Frequency
Chr1
Frequency
0
2000
Allele Frequency
Chr2
Frequency
0
2000
Allele Frequency
Chr3
Frequency
0
1500
Allele Frequency
Chr4
Frequency
0
1500
Allele Frequency
Chr5
Frequency
0
1000
Allele Frequency

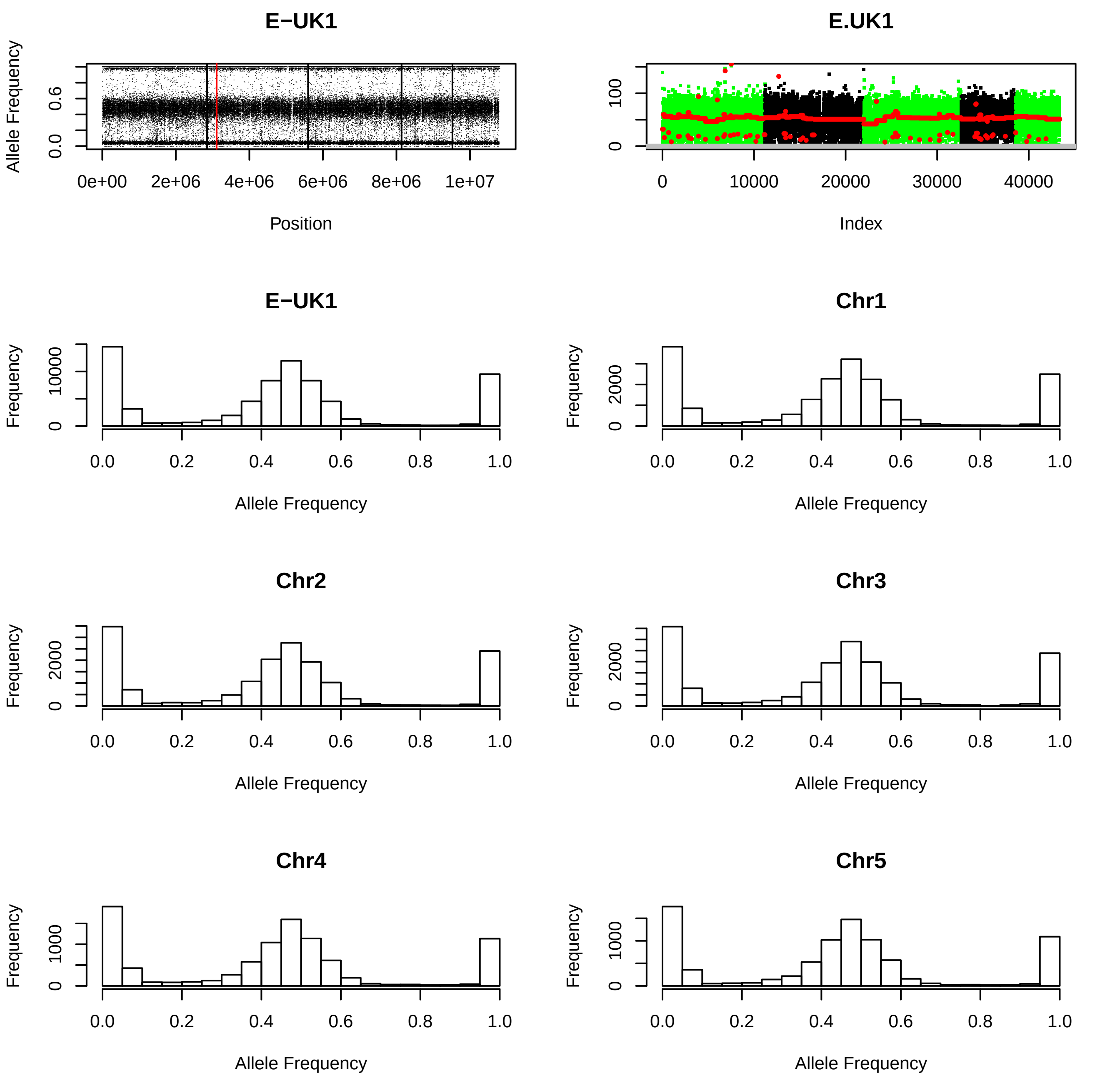
E−UK1
Allele Frequency
0.0
0.6
0e+00
2e+06
4e+06
6e+06
8e+06
1e+07
Position
E.UK1
0
100
0
10000
20000
30000
40000
Index
E−UK1
Frequency
0
10000
Allele Frequency
Chr1
Frequency
0
2000
Allele Frequency
Chr2
Frequency
0
2000
Allele Frequency
Chr3
Frequency
0
2000
Allele Frequency
Chr4
Frequency
0
1000
Allele Frequency
Chr5
Frequency
0
1000
Allele Frequency
0.0
0.2
0.4
0.6
0.8
1.0

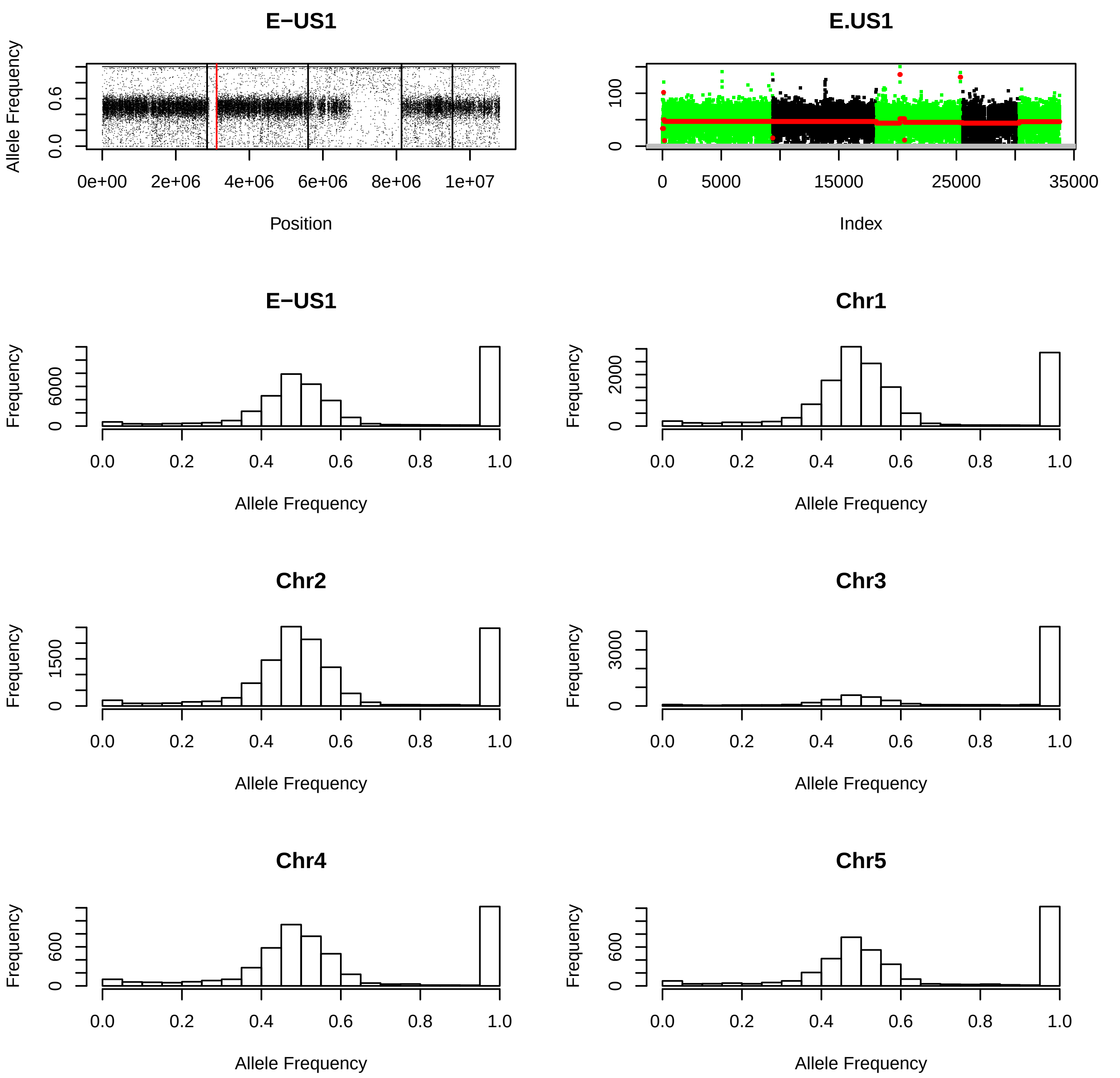
E−US1
Allele Frequency
0.0
0.6
0e+00
2e+06
4e+06
6e+06
8e+06
1e+07
Position
E.US1
0
100
0
5000
15000
25000
35000
Index
E−US1
Frequency
0
6000
Chr1
0
2000
Chr2
0
1500
Chr3
0
3000
Chr4
0
600
Chr5
0
600
0.0
0.2
0.4
0.6
0.8
1.0
Allele Frequency

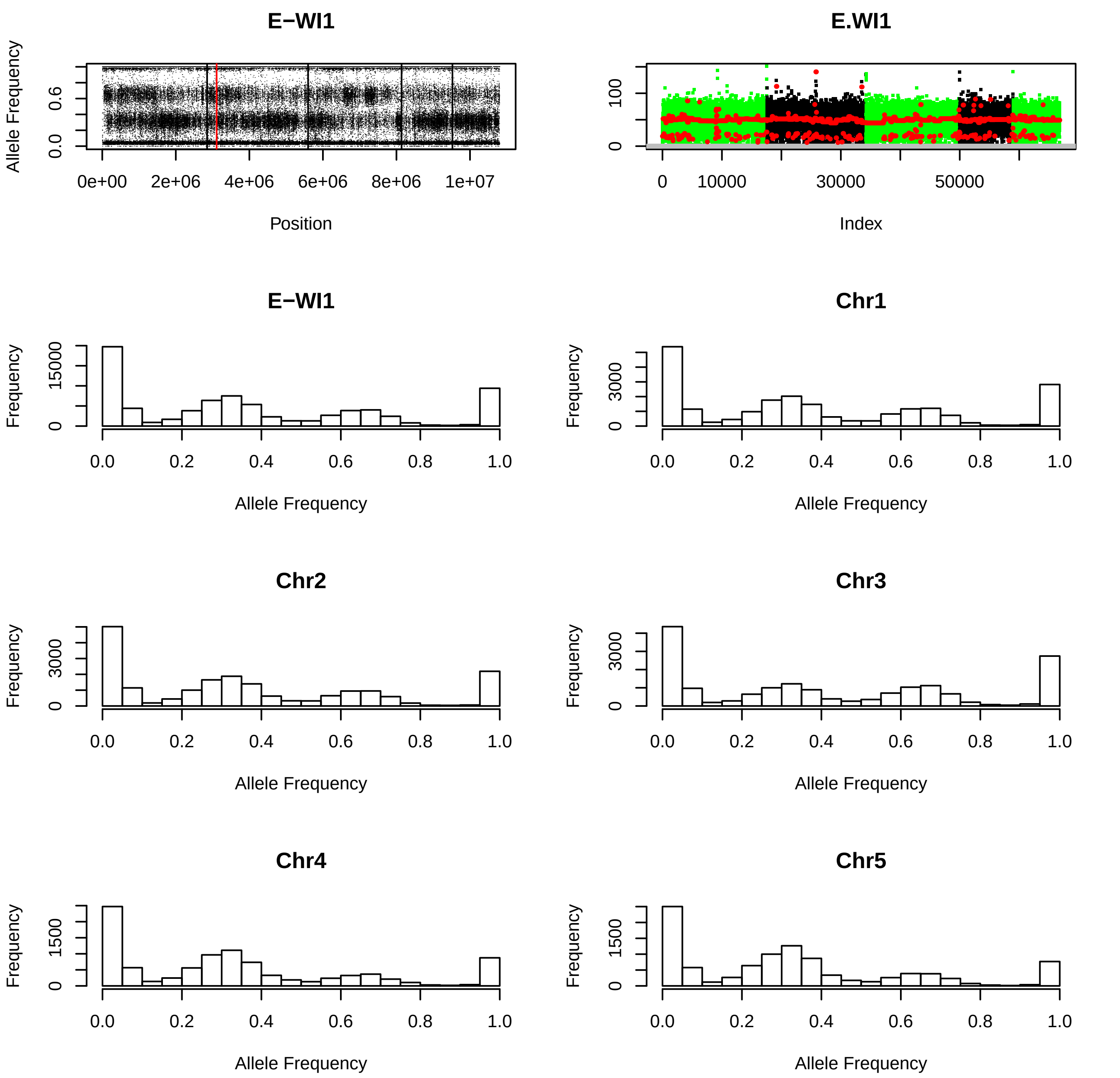
E−WI1
Allele Frequency
0.0
0.6
0e+00
2e+06
4e+06
6e+06
8e+06
1e+07
Position
E.WI1
0
100
0
10000
30000
50000
Index
E−WI1
Frequency
0
15000
0.0
0.2
0.4
0.6
0.8
1.0
Allele Frequency
Chr1
Frequency
0
3000
Allele Frequency
Chr2
Frequency
0
3000
Allele Frequency
Chr3
Frequency
0
3000
Allele Frequency
Chr4
Frequency
0
1500
Allele Frequency
Chr5
Frequency
0
1500
Allele Frequency
